# Systematic evaluation of machine learning models for postoperative surgical site infection prediction

**DOI:** 10.1371/journal.pone.0312968

**Published:** 2024-12-12

**Authors:** Anna M. van Boekel, Siri L. van der Meijden, Sesmu M. Arbous, Rob G. H. H. Nelissen, Karin E. Veldkamp, Emma B. Nieswaag, Kim F. T. Jochems, Jeroen Holtz, Annekee van IJlzinga Veenstra, Jeroen Reijman, Ype de Jong, Harry van Goor, Maryse A. Wiewel, Jan W. Schoones, Bart F. Geerts, Mark G. J. de Boer

**Affiliations:** 1 Department of Internal Medicine, Leiden University Medical Center, Leiden, The Netherlands; 2 Department of Intensive Care, Leiden University Medical Center, Leiden, The Netherlands; 3 Healthplus.ai R&D B.V., Amsterdam, The Netherlands; 4 Department of Orthopedic surgery, Leiden University Medical Center, Leiden, The Netherlands; 5 Department of Medical Microbiology and Infection Control, Leiden University Medical Center, Leiden, The Netherlands; 6 Department of Clinical Epidemiology, Leiden University Medical Center, Leiden, The Netherlands; 7 Department of Surgery, Radboud UMC, Nijmegen, The Netherlands; 8 Waleus Medical Library, Leiden University Medical Center, Leiden, The Netherlands; 9 Department of Infectious disease, Leiden University Medical Center, Leiden, The Netherlands; Mayo Clinic Rochester, UNITED STATES OF AMERICA

## Abstract

**Background:**

Surgical site infections (SSIs) lead to increased mortality and morbidity, as well as increased healthcare costs. Multiple models for the prediction of this serious surgical complication have been developed, with an increasing use of machine learning (ML) tools.

**Objective:**

The aim of this systematic review was to assess the performance as well as the methodological quality of validated ML models for the prediction of SSIs.

**Methods:**

A systematic search in PubMed, Embase and the Cochrane library was performed from inception until July 2023. Exclusion criteria were the absence of reported model validation, SSIs as part of a composite adverse outcome, and pediatric populations. ML performance measures were evaluated, and ML performances were compared to regression-based methods for studies that reported both methods. Risk of bias (ROB) of the studies was assessed using the Prediction model Risk of Bias Assessment Tool.

**Results:**

Of the 4,377 studies screened, 24 were included in this review, describing 85 ML models. Most models were only internally validated (81%). The C-statistic was the most used performance measure (reported in 96% of the studies) and only two studies reported calibration metrics. A total of 116 different predictors were described, of which age, steroid use, sex, diabetes, and smoking were most frequently (100% to 75%) incorporated. Thirteen studies compared ML models to regression-based models and showed a similar performance of both modelling methods. For all included studies, the overall ROB was high or unclear.

**Conclusions:**

A multitude of ML models for the prediction of SSIs are available, with large variability in performance. However, most models lacked external validation, performance was reported limitedly, and the risk of bias was high. In studies describing both ML models and regression-based models, one modelling method did not outperform the other.

## Introduction

Surgical site infections (SSIs) are known complications following surgery and belong to the most frequently occurring hospital-acquired infections. The incidence of SSIs ranges between 0.6% and 18% and depend on the type of surgical procedure and setting [1–4]. Surgical site infections lead to increased morbidity, mortality and hospital stay, resulting in a negative impact on the patient’s health-related quality of life [2]. Moreover, SSIs cause an increase in healthcare costs due to prolonged hospitalization, the need for extra diagnostic tests and interventions, and prolonged treatment. Recent meta-analyses showed an additional length of hospital stay between 2.1 to 54 days for patients with an SSI [2] with an estimated cost ranging from USD $10,443 to USD$ 25,546 per case [3]. Early detection and treatment are important for reducing these negative effects of SSIs.

Several risk factors for the development of SSIs have been identified such as sex, BMI, comorbidity American Society of Anesthesiologist (ASA) score, smoking, age and surgical approach [5, 6]. Several prognostic prediction models have been developed to identify which patients are at risk for developing an SSI. Besides traditional models, such as those using logistic regression [7], machine learning (ML) models are increasingly being developed and used for this purpose. ML comprises a wide spectrum of different algorithms that automatically learn from presented and new input data in a continuous iterative process, and variable selection for ML models is performed by these algorithms. This in contrast to traditional models, where variable selection and internal model settings are more dictated by humans [8–10]. ML models benefit not only from this iterative learning process, but also from using more and different types of input variables. The complex algorithmic structure can find non-linear relations between variables, which contrasts with traditional regression-based models [11]. The disadvantage of ML models is that the outcomes result in “black-box” predictions, where the used data for ML model output, the (relative) importance of these data and their possible mutual effects are less evident compared to regression-based models [12, 13].

To evaluate the statistical performance of prediction models, discriminative performance in terms of concordance statistics (C-statistic), also known as area under the receiver operating characteristic curve (ROC or area under the curve -AUC-), and calibration in terms of calibration plots with slope and intercept are most often assessed [14]. Discriminative performance is the ability of the model to distinguish between patients with and without the outcome, whereas calibration is the agreement between the predicted probability and the proportion of patients with the actual outcome. Prediction models are first internally validated, using for example cross-validation or bootstrapping. Thereafter, external validation should be performed either on other hospital datasets, prospectively in time, or both, to ensure generalizability [15].

ML models are increasingly being developed for many different purpuses in surgery [7]. Elfanagely et al [16] described 45 ML models used for the prediction of surgical outcomes and another review [17] summarized the outcomes of 212 articles with ML models developed for prediciting a broad spectrum of outcomes in vascular surgery. The ML models performed reasonably well, but there were concerns regarding the risk of bias. A recent systematic review and meta-analysis performed by Wu et al. showed that there are many different ML models for the prediction or detection of SSIs, but that the validation of these models is generally insufficient [18]. Wu et al. mainly focused on the methodological aspects of the models and made no distinction between the prediction of SSI or SSI detection for surveillance purposes. Moreover, a clear overview of the available models for different surgical specialties or SSI subtypes (superficial-, deep- or organ space SSI) is still missing. The number of models developed for SSI prediction is increasing, and since 2021 new models have been developed. The aim of this systematic review was therefore to describe the performance of all internally or externally validated ML models for the prediction of SSI, to describe the methodological quality of the studies studying ML models for prediction of SSI, and to give an overview of the available models per surgical specialty and SSI subtype.

## Methods

A systematic review of the published literature on the prediction of postoperative infections was conducted according to the Preferred Reporting items for Systematic Reviews and Meta-Analyses (PRISMA) statement (S1 Appendix). The protocol for this study was registered in PROSPERO (registration number 248953).

### Search strategy

The literature search was performed in MEDLINE, EMBASE and the Cochrane Library from inception to July 1, 2023. The complete search strings are shown in the Supplementary material (S2 Appendix).

### Inclusion and exclusion criteria

All original studies that developed and validated (internally or externally) ML models for the prediction of SSIs and studies that externally validated ML models that were previously developed were included. Models were considered to be an ML model if a non-regression-based approach for model development was used such as random forests, support vector machines and neural networks. As outcome, prediction of all types of SSIs within 30 days postoperative were included. Models that only predicted SSIs as part of a composite adverse outcome were excluded. Other exclusion criteria were pediatric populations (age <18 years old), no full text article available, and articles not written in English language.

### Screening and data extraction

Study selection was performed using the Covidence^®^ software program (www.covidence.org, Melbourne, Australia). After removal of duplicates, titles and abstracts were screened on full text inclusion criteria by two independent authors (AB, BG, or MW). Full text analysis of the remaining articles was performed by the same authors. All conflicts were resolved by a third reviewer (MB or SA).

The following data were obtained from the included articles: type of SSI predicted (either superficial, deep or organ space), surgical specialty, number of surgeries, patients or both, performance parameters of the model (sensitivity, specificity, accuracy, calibration and C-statistic), method of validation, variables used as predictors, and all types of developed and/or validated models (ML as well as regression-based models). A complete list of the extracted data is provided in S2 Table. Reviewers used a standardized data extraction form that was based on the CHARMS (CHecklist for critical Appraisal and data extraction for systematic Reviews of prediction Modelling Studies) [19]. Extracted data was double checked for inconsistencies by AB and BG and discrepancies were resolved by consensus.

### Descriptive analyses

Results were summarized using descriptive statistics. We did not perform a meta-analysis due to the heterogeneity in reported outcome measures and definitions. Analyses were performed using R (version 2023.06.1+524, R Core Team, Vienna, Austria).

### Risk of bias

The methodological quality of all included studies was assessed using the Prediction model study Risk of Bias Assessment Tool (PROBAST) [20, 21]. The PROBAST is designed to critically appraise prediction models and contains two main domains: the risk of bias, which consists of four subdomains (participant selection, predictor selection, outcome definition and analysis) and the applicability for the review. In total there are 20 signaling questions which can be scored as ‘yes’, ‘probably yes’, ‘probably no’, ‘no’, or ‘no information’ which combined lead to a low, uncertain, or high risk of bias and applicability.

## Results

**Fig 1:**
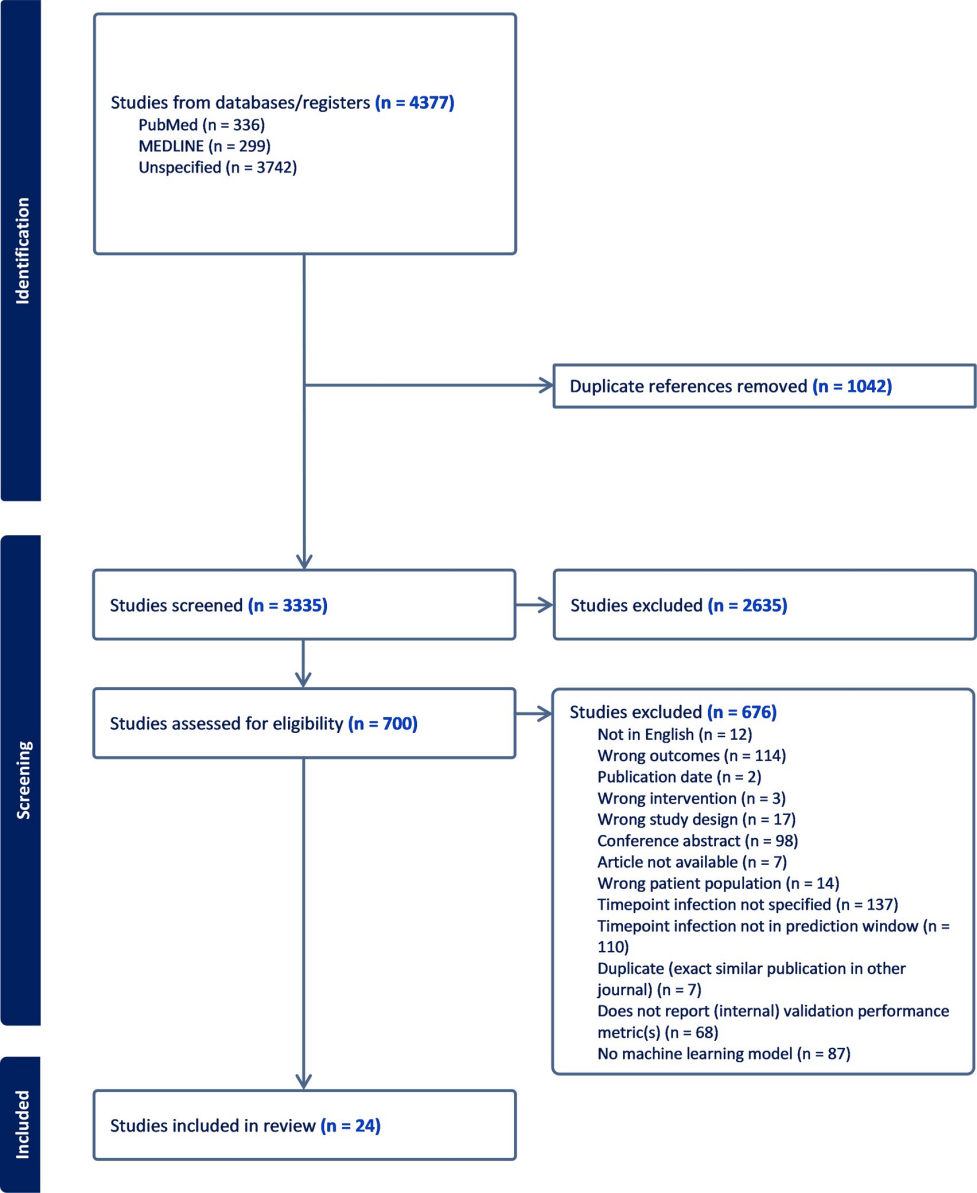
PRISMA figure.

A flowchart of the search is summarized in Fig 1. Of the 4,377 publications identified, 24 studies were included for further analysis. See S1 Table for the exclusion reasons of the excluded full text articles.

### Characteristics of included studies

The 24 included studies described a total of 85 different ML models. Sixty-nine models (81%) were internally validated and 16 (19%) were externally validated, including one model (the Predictive OpTimal Trees in Emergency Surgery Risk (POTTER) Calculator) that was externally validated in five separate studies. The most frequently predicted outcome was SSI in general (i.e., a combination of superficial-, deep- and organ space SSI or unspecified), 11 models predicted superficial SSI, nine models predicted deep SSI and 24 models predicted organ space SSI. Abdominal surgery was the surgical specialty for which most models were developed (47%), followed by general surgery (21%) and orthopedic surgery (8%). See Table 1 for an overview of all included studies.

**Table 1:** Overview of included studies.

Author & year	Number of ML models	Country	Specialty	Type of infection predicted	Sample size (n)	Type of validation
**Bertsimas** **2018 [22]**	2	USA	Emergency surgery	Superficial SSI, deep SSI, organ space SSI	Development 382,960 Validation unknown	Model development, internal and external validation
**Bonde** **2021 [23]**	4	USA	General surgery	Superficial SSI, deep SSI, organ space SSI	Training 4,694,488 Validation 173,622 Testing 13,771	Model development, internal and external validation
**Chang** **2020 [24]**	1	USA	Vascular surgery	SSI	72,435 split into 80–20 training ‐ validation cohort 370 institution cohort.	Internal
**El Hechi** **2021 [25]**	1	USA	Emergency surgery	Superficial SSI, deep SSI, organ space SSI	59,955	External
**Gowd** **2019 [26]**	5	USA	Orthopedic surgery	SSI	17,119 split into 80–20 training-validation cohort	Internal
**Grass** **2021 [27]**	2	USA	Abdominal surgery	SSI	ACS-NSQIP database: Model development 180,538 External validation 2,376 Mayo clinic database: 2,376 (10-fold cross validation)	ACS-NSQIP database: Model development, internal and external validation Mayo-clinic: internal
**Ke** **2017 [28]**	1	The Netherlands	Abdominal surgery	SSI	860	Internal
**Liu (W.C.)** **2022 [29]**	5	China	Neurosurgery	SSI	Training set 201 Test set 87	Internal
**Liu (X.)** **2022 [30]**	4	China	Abdominal surgery	Organ space SSI	297 split into training (81%), validation (9%) and testing (10%) set.	Internal
**Mamlook** **2023 [31]**	6	USA	General	SSI	2,882,526	Internal
**Maurer** **2020 [32]**	1	USA	Emergency surgery	Superficial SSI, deep SSI, organ space SSI	29,366	External
**Mazaki** **2021 [33]**	1	Japan	Abdominal surgery	Organ space SSI	256	Internal
**Merath** **2020 [34]**	1	USA	Abdominal surgery	Superficial SSI, deep SSI, organ space SSI	15,657	Internal
**Nudel** **2020 [35]**	2	USA	Abdominal surgery	Organ space SSI	436,807 split into training (218,403), validation (109,202) and testing (109,202)	Internal
**Ohno** **2022 [36]**	1	Japan	Abdominal surgery	SSI	730 development 100 validation	Internal
**Sanger** **2016 [37]**	4	The Netherlands	Abdominal Surgery	SSI	851	Internal
**Taylor** **2019 [38]**	4	USA	Urological surgery	SSI	7,557 split into 80% training and 20% testing set.	Internal
**Van Esbroeck 2014 [39]**	5	USA	General surgery	SSI	Training 607,558 Validation 363,875 Evaluation (external validation) 363,431	Model development, internal and external validation
**Van Kooten 2022 [40]**	6	The Netherlands	Abdominal surgery	Organ space SSI	6,427 split into 75% training and 25% testing set	Internal
**Velmahos** **2023 [41]**	2	USA	Abdominal surgery	Superficial SSI, organ space SSI	94,530 split into 75% training and 25% testing set	Internal
**Walczak** **2019 [42]**	3	USA	General surgery	SSI	646	Internal
**Weller** **2018 [43]**	16	USA	Abdominal surgery	SSI	9,598 split into 80% training and 20% testing set	Internal
**Ying** **2023 [44]**	2	China	Orthopedic	SSI	351	Model development, internal and external validation
**Zhang** **2023 [45]**	6	China	Cardiothoracic	SSI	1,223 split into training (858) and validation (365) set	Internal

ACS-NSQIP, American College of Surgeons National Surgical Quality Improvement Program; SSI, Surgical Site Infection; USA, United States of Ameri.

### Performance of ML models

The most common reported outcome for model performance was the C-statistic, which was reported in 96% of the studies. Other model performance parameters reported were sensitivity, specificity, negative predicting value and positive predicting value. Only two studies reported calibration metrics of which one study also reported the brier score [39, 44]. Of the internally validated models, the median C-statistic was 0.62 and ranged from 0.44 to 0.99, for the externally validated models the median C-statistic was 0.79 and ranged from 0.55 to 0.87. Sensitivity, specificity, negative predictive value (NPV) and positive predictive value (PPV) were reported in one externally validated model by Grass et al. and were 0.47, 0.8, 0.97 and 0.10 respectively. Of the internally validated models, sensitivity was reported for twenty (29%) models and varied between 0.24 to 0.90, specificity was reported for fifteen (22%) models and varied between 0.25 to 0.91, NPV was reported for four (6%) models and varied between 0.87 to 0.98 and PPV was reported for eleven (16%) models and varied between 0.06 to 0.90 respectively. Overall, the performance of the models varied widely and there was no clear difference between the different surgical specialties or type of SSI predicted (Tables 2–5).

**Table 2:** Performance of ML models predicting SSI in general per surgical specialism.

Author	Algorithm	Number of predictors	C-statistic	Sensitivity	Specificity	NPV	PPV	Validation
**Cardiothoracic surgery**								
**Zhang** **2023 [45]**	RF	-	0.83	-	-	-	-	Internal
	SVM	-	0.91	-	-	-	-	Internal
	XGB	-	0.99	-	-	-	-	Internal
	GBDT	-	0.99	-	-	-	-	Internal
	Adaboost	-	0.81	-	-	-	-	Internal
	NN	-	0.99	-	-	-	-	Internal
**Abdominal surgery**								
**Grass** **2021 [27]**	BPMI	-	0.74	0.47	0.8	0.97	0.10	External
	BPMI–Mayo clinic	-	0.78	0.56	0.8	0.98	0.11	Internal
**Ke** **2017 [28]**	Bilinear model	≥28	-	-	-	-	-	Internal
**Ohno** **2022 [36]**	SVM	25	0.73	-	-	-	-	Internal
**Sanger** **2016 [37]**	NB ‐ Baseline features	28	0.63	-	-	-	-	Internal
	NB ‐ Serial features	9	0.74	0.42–0.80*	0.64–0.91*	0.87–0.92*	0.33–0.53*	Internal
	NB ‐ Serial features simplified	5	0.73	0.42–0.75*	0.64–0.91*	0.87–0.92*	0.35–0.53*	Internal
	NB ‐ Combined features	37	0.75	-	-	-	-	Internal
**Weller** **2018 [43]**	RF	-	0.44^a^, 0.47^b^, 0.50^c^, 0.55^d^	-	-	-	-	Internal
	SVM	-	0.55^a^, 0.51^b^, 0.47^c^, 0.49^d^	-	-	-	-	Internal
	AdaBoost	-	0.44^a^, 0.47^b^, 0.51^c^, 0.51^d^	-	-	-	-	Internal
	NB	-	0.48^a^, 0.45^b^, 0.45^c^, 0.78^d^	-	-	-	-	Internal
**General surgery**								
**Van Esbroeck** **2014 [39]**	SVM–short description	-	0.79	-	-	-	-	External
	SVM–medium description	-	0.79	-	-	-	-	External
	SVM–large description	-	0.79	-	-	-	-	External
	SVM–CPT	-	0.74	-	-	-	-	External
	SVM–multivariate model	-	0.80	-	-	-	-	External
**Mamlook** **2023 [31]**	Naïve bayes	-	0.71	0.76	0.71	-	-	Internal
	Random forest	-	0.83	0.84	0.84	-	-	Internal
	Decision tree	-	0.81	0.82	0.81	-	-	Internal
	SVM	-	0.82	0.82	0.82	-	-	Internal
	ANN	-	0.82	0.81	0.81	-	-	Internal
	DNN	16	0.85	0.85	0.85	-	-	Internal
**Walczak** **2019 [42]**	ANN–all variables	11	-	0.69	0.60	-	-	Internal
	ANN without NSQIP compliance	10	-	0.90	0.50	-	-	Internal
	ANN without NSQIP compliance and sex	9	-	0.79	0.50	-	-	Internal
**Orthopedic surgery**								
**Gowd** **2019 [26]**	KNN	22	0.5	-	-	-	-	Internal
	RF	22	0.53	-	-	-	-	Internal
	NB	22	0.45	-	-	-	-	Internal
	Decision tree	22	0.5	-	-	-	-	Internal
	Gradient boosting trees	22	0.61	-	-	-	-	Internal
**Ying** **2023 [44]**	Extra trees classifier	-	0.87	-	-	-	-	External
	Random forest	-	0.82	-	-	-	-	External
**Neurosurgery**								
**Liu** **2022 [29]**	Decision tree	-	0.78	-	-	-	0.79	Internal
	Multilayer perception	-	0.76	-	-	-	0.66	Internal
	Random forest	-	0.89	-	-	-	0.86	Internal
	Gradient boosting machine	-	0.91	-	-	-	0.88	Internal
	Extreme gradient boosting machine	-	0.92	-	-	-	0.9	Internal
**Urological surgery**								
**Taylor** **2019 [38]**	GAM	-	∼ 0.61**	-	-	-	-	Internal
	LASSO logistic regression	-	0.62	-	-	-	-	Internal
	RF	-	∼ 0.61**	-	-	-	-	Internal
	NNET	-	∼ 0.58**	-	-	-	-	Internal
**Vascular surgery**								
**Chang** **2020 [24]**	DLPM	-	0.61	0.83	0.25	0.912	0.14	Internal

AdaBoost, Adaptive boosting; ANN, Artificial neural network; BPMI, Bayesian-probit regression model with multiple imputation; DLPM, Deep learning based risk model; GAM, Generalize additive models; GBDT, Gradient boosted decision trees; GLM, Generalized linear model; KNN, K-nearest neighbors; NB, Naïve Bayes; NNET, Feed-forward neural network with logistic activation function and no weight decay; OCT, optimal classification trees; POD, postoperative day; POTTER, Predictive OpTimal Trees in Emergency Surgery Risk; RF, Random forest; SVM, Support vector machine; XGB, Gradient Boosting machine

*Depending on cut-off value sensitivity and specificity

**No exact value given.

^a^preoperative

^b^POD0

^c^POD1

^d^POD2

**Table 3:** Performance of ML models predicting superficial SSI per surgical specialism.

Author	Algorithm	Number of predictors	C-statistic	Validation
**Abdominal surgery**				
**Merath** **2020 [34]**	Decision tree	-	0.76	Internal
**Velmahos** **2023 [41]**	Random forest	-	0.63	Internal
	XGB	-	0.64	Internal
**General surgery**				
**Bonde** **2021 [23]**	ANN model 1	21	0.82	External
	ANN model 2	34	0.82	External
	ANN model 3	56	0.83	External
**Emergency surgery**				
**Bertsimas** **2018 [22]**	OCT POTTER–without ASA	-	0.68	External
	OCT POTTER with ASA		0.68	External
**Bonde** **2021 [23]**	ANN model 1	21	0.75	External
	ANN model 2	21	0.75	External
	ANN model 3	21	0.76	External
	OCT POTTER	-	0.68	External
**El Hechi** **2021 [25]**	OCT POTTER	-	General 0.64 Laparotomy 0.55	External
**Maurer** **2020 [32]**	OCT POTTER	-	0.60–0.80**	External

ANN, Artificial neural network; ASA, American Society of Anesthesiology; OCT, optimal classification trees; POTTER, Predictive OpTimal Trees in Emergency Surgery Risk; XGB, Gradient Boosting machine.

**No exact value given.

**Table 4:** Performance of ML models predicting deep SSI per surgical specialism.

Author	Algorithm	Number of predictors	C-statistic	Validation
**Abdominal surgery**				
**Merath** **2020 [34]**	Decision tree	-	0.93	Internal
**General surgery**				
**Bonde** **2021 [23]**	ANN model 1	21	0.78	External
	ANN model 2	34	0.78	External
	ANN model 3	56	0.80	External
**Emergency surgery**				
**Bertsimas** **2018 [22]**	OCT POTTER–without ASA	-	0.74	External
	OCT POTTER with ASA		0.75	External
**Bonde** **2021 [23]**	ANN model 1	21	0.87	External
	ANN model 2	21	0.85	External
	ANN model 3	21	0.82	External
	OCT POTTER	-	0.75	External
**El Hechi** **2021 [25]**	OCT POTTER	-	General 0.70 Laparotomy 0.60	External
**Maurer** **2020 [32]**	OCT POTTER	-	0.60–0.80**	External

ANN, Artificial neural network; ASA, American Society of Anesthesiology; OCT, optimal classification trees; POTTER, Predictive OpTimal Trees in Emergency Surgery Risk.

**No exact value given.

**Table 5:** Performance of ML models predicting organ space SSI per surgical specialism.

Author	Algorithm	Number of predictors	C-statistic	Validation
**Abdominal surgery**				
**Liu2022 [30]**	GBDT	-	0.83	Internal
	KNN	-	0.71	Internal
	Random forest	-	0.87	Internal
	SVM	-	0.89	Internal
**Mazaki2021 [33]**	ANN	18	0.77	Internal
**Merath** **2020 [34]**	Decision tree	-	0.76	Internal
**Nudel2020 [35]**	XGB	33	0.70	Internal
	ANN	-	0.75	Internal
**Van Kooten2022 [40]**	AdaBoost	-	0.61	Internal
	Adalearner	-	0.62	Internal
	KNN	-	0.57	Internal
	NN	-	0.61	Internal
	Random forest	-	0.59	Internal
	SVM	-	0.59	Internal
**Velmahos2023 [41]**	Random forest	-	0.72	Internal
	XGB	-	0.72	Internal
**General surgery**				
**Bonde** **2021 [23]**	ANN model 1	21	0.85	External
	ANN model 2	34	0.85	External
	ANN model 3	56	0.87	External
**Emergency surgery**				
**Bertsimas** **2018 [22]**	OCT POTTER–without ASA	-	0.78	External
	OCT POTTER with ASA	-	0.79	External
**Bonde** **2021 [23]**	ANN model 1	21	0.78	External
	ANN model 2	21	0.81	External
	ANN model 3	21	0.86	External
	OCT POTTER	-	0.79	External
**El Hechi** **2021 [25]**	OCT POTTER	-	General 0.69 Laparotomy 0.62	External
**Maurer** **2020 [32]**	OCT POTTER	-	0.80**	External

AdaBoost, Adaptive boosting; ANN, Artificial neural network; ASA, American Society of Anesthesiology; GBDT, Gradient boosted decision trees; KNN, K-nearest neighbors; OCT, optimal classification trees; POTTER, Predictive OpTimal Trees in Emergency Surgery Risk; SVM, Support vector machine; XGB, Gradient Boosting machine.

**No exact value given.

### Predictors used in ML models

**Fig 2:**
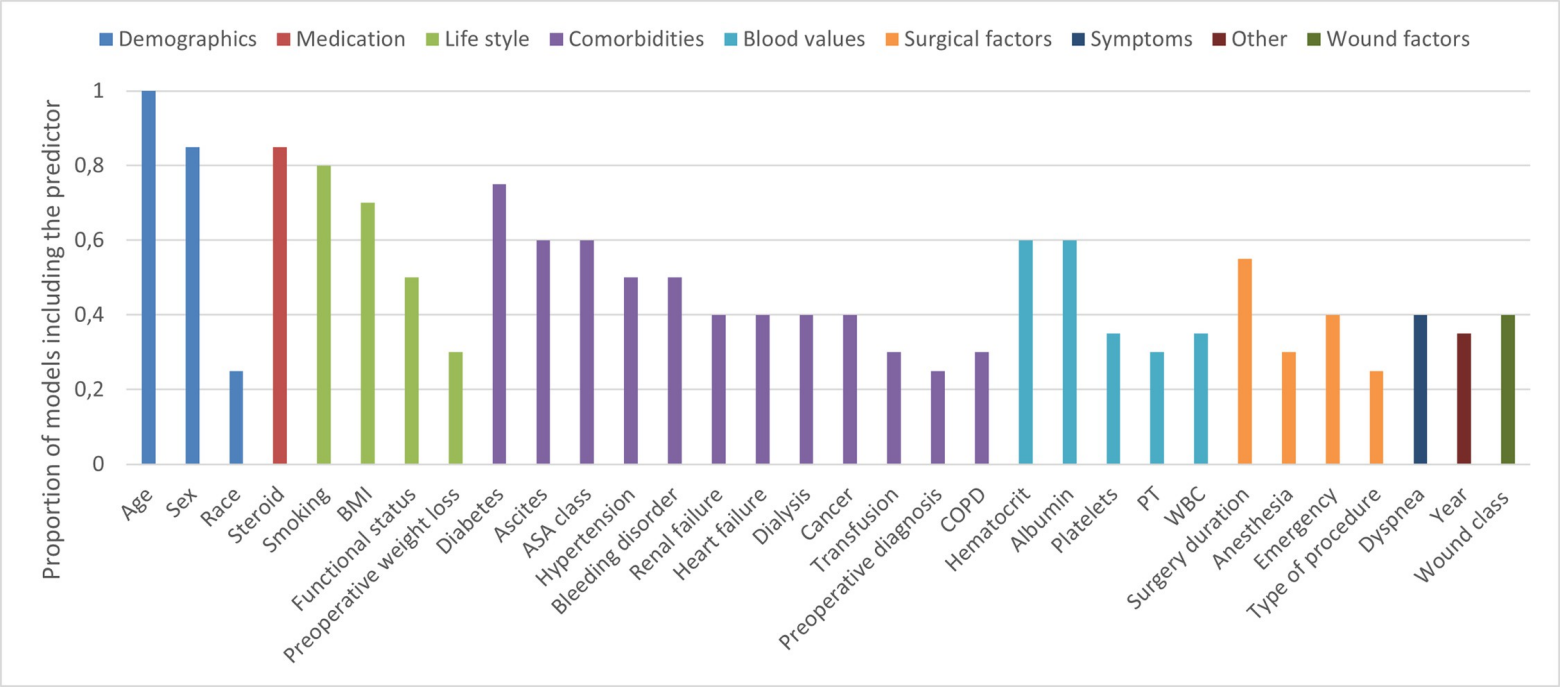
Predictors used in proportion of the ML models. All predictors used five times or more are included in the figure. ASA classification (American Society of Anesthesiologists); BMI, (Body Mass Index); COPD (Chronic Obstructive Pulmonary Disease); INR, (International Normalized Ratio); PT, (Prothrombin time); WBC, (White blood count).

Of the 85 included ML models, the number of predictors used in the model was reported for 20 models (24%), with mentioning of feature importance (determined by SHAP values) in 15 models (18%). In total 116 different predictors were used in these 20 models. The median number of included predictors per model was 22, ranging from 5–56. The most commonly included predictors were age (100%), oral corticosteroid use (85%), sex (85%), smoking (80%), and diabetes (75%) (Fig 2).

### Regression-based models

**Fig 3:**
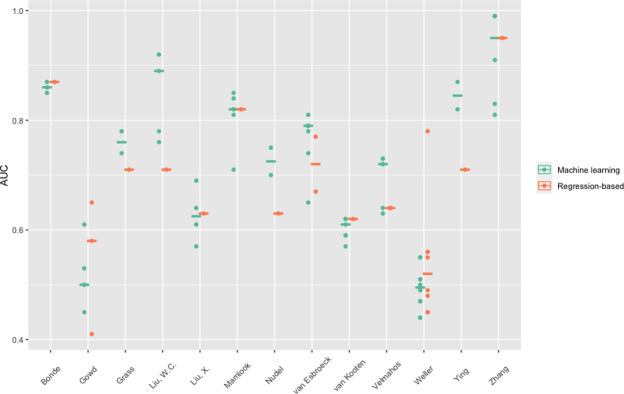
Area under the curve (AUC) for each article that presented both ML and regression-based models. Green dots represent the AUC of the ML models, orange dots represent the AUC of the regression-based models. The green and orange lines represent the median.

Of the 24 studies, thirteen studies (54%) also included regression-based models and compared the regression-based performance to the performance of their developed ML models (Fig 3 and S3 Table). The C-statistic for regression-based models varied between 0.41 to 0.95. For the prediction of SSIs, ML performed slightly better compared to regression-based models in four studies [27, 29, 35, 44], whereas regression-based models performed better in two studies [26, 41]. In the other studies reporting both regression-based and ML models, performances were similar [23, 30, 31, 39, 40, 43, 45]. See Fig 3 for an overview of the AUCs of the studies presenting both ML and regression-based models.

### Risk of bias

**Fig 4:**
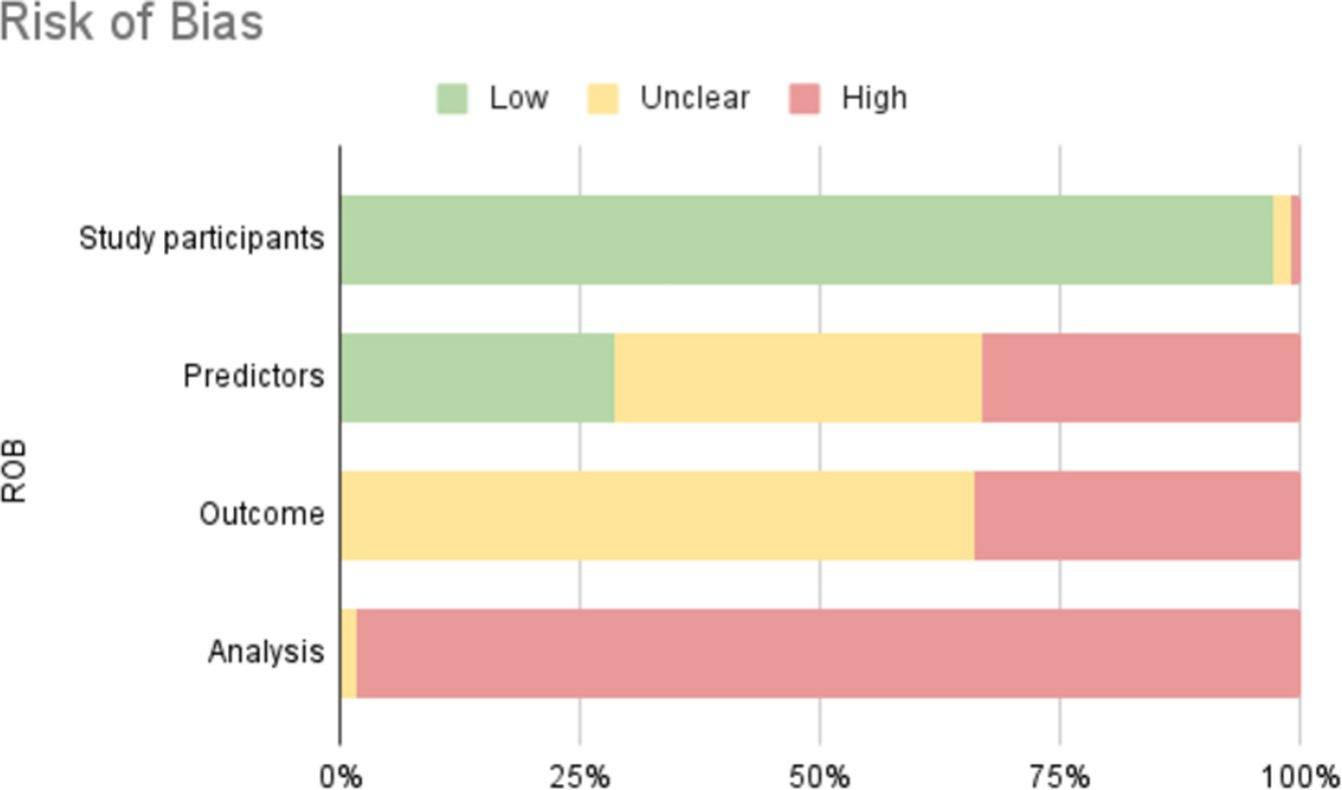
Summary of risk of bias assessment using the PROBAST. Green low risk of bias, yellow unclear risk of bias due to lack of information, red high risk of bias. ROB; Risk of bias.

The ROB was assessed for all models described in the 24 studies. ROB was low in the participants domain. ROB was high or unclear in the predictors domain and outcome domain, as studies often poorly reported the used predictors and whether predictors were selected independent of the outcome status. In the analysis domain, all studies had a high or unclear ROB, mostly caused by statistical issues such as poor reporting of performance measures, not taking competing risks into account and inappropriate methods to handle missing data. There were no concerns on applicability for all studies. See Fig 4 for an overview of ROB, and S4 Table for the complete ROB.

## Discussion

This systematic review showed that a multitude of 85 different validated ML prediction models for SSIs exists. Most models were developed and tested in patient populations that underwent abdominal surgery. Most of these models (81%) were only internally validated. The most frequently reported parameter for performance was the C-statistic, which varied widely between the different models, and only two studies reported calibration metrics. This corresponds with previous studies on the use of ML in other fields, that found that calibration is rarely reported and that only a minority of the models is externally validated [11, 46, 47]. However, for proper assessment of model performance, both discrimination and calibration are essential parameters for the interpretation of the predicted probabilities [14]. Without external validation of a prediction model, it is difficult to accurately estimate the actual performance of a model in different clinical practices. Furthermore, it is common that retraining or recalibration of an ML model is necessary to fit the unseen population [48]. Therefore, newly developed ML prediction models as well as already existing models need to be retrained, recalibrated, and again validated for new populations. Furthermore, their effect on patient care should then be evaluated and reported with impact studies.

Thirteen of the included studies described both regression-based and ML models and compared their performances in the same population. Both the regression-based models as well as the ML models showed large variability of performance, which is in accordance with previous literature on regression-based models for the prediction of SSIs [49–51]. When compared, the ML and regression-based models did not outperform each other. This is in accordance with previous studies that compared ML models with regression-based models, although some studies suggest that certain subtypes of ML (i.e. gradient boosting trees) perform better than regression-based models [52, 53]. ML models generally need larger datasets to use their full potential. It is possible that this condition was not met in all studies, as the median number of predictors was 22 and the sample size ranged from 256 to 5,881,881.

Model explainability is an important issue with ML prediction models. In general, ML models are considered to be more complex and less transparent with respect to which variables are selected for the prediction compared to regression-based models. Furthermore, in our study, transparency of ML models was further limited as only in the minority of the ML models (24%) the used predictors were reported. This contrasts with regression-based models which are usually presented with regression coefficients representing the strength of the relation between individual predictors and the outcome [54]. Despite being less transparent, ML models are able to utilize large and heterogenous number of datasets and types, can take into account more complex relationships of predictors, can be adapted to the local setting if the model has been validated or recalibrated to this population and can be incorporated in the electronic health care system, making them potentially more beneficial when implemented into clinical care [10].

The ROB was high or unclear for almost all studies, suggesting considerable methodological issues. ROB was scored using the PROBAST which is the most common used tool to estimate ROB of prediction studies. Although an high or unclear ROB for almost all studies is in agreement with previous reviews using the PROBAST [55–57], the PROBAST has been criticized because of poor inter-rater agreement [56, 57]. Moreover, it is not possible to distinguish domains with a high ROB based on one single signaling question answered with ‘no’ from domains with all signaling questions answered with ‘no’. Despite the limitations of the PROBAST, it remains a useful tool to assess methodological shortcomings in prediction studies. Therefore, caution for the interpretation of the findings from these ML models for SSI prediction is recommended. Recently, the new TRIPOD-AI guidelines have been published and new ML models being developedshould follow these guidelines in order to prevent bias [58].

### Strengths and limitations

The major strength of this systematic review is that it included all presently available validated ML models for the prediction of SSIs without restrictions on surgical specialty or SSI subtype. In addition, we described the comparison of regression-based models with ML models where possible. As both types of models were compared to each other within the same population, bias was minimalized.

#### Some limitations exist

Differences in the quality and the heterogeneity of the data prevented the conduction of a sound meta-analysis comparing the different ML models. Furthermore, this review is limited to SSIs as outcome, although other postoperative infections such as pneumonia and bloodstream infections are also clinically relevant.

## Conclusions

This systematic review showed that many ML models for the prediction of SSIs exist, and that their performance generally is equal to regression-based models. Machine learning techniques are still developing and are seen as a promising tool to improve medical care. However, there are multiple methodological issues with the currently available models and there is still a substantial gap between the existing models and their practical and safe implementation in clinical settings. The recently published TRIPOD-AI guidelines should be used to reduce methodological flaws. To create clinically relevant prediction models for future use, more collaboration between clinicians and data scientists, as well as post-implementation studies are needed.

## Supporting information

S1 Appendix Prisma checklist. (DOCX)

S2 Appendix Search strategy. (DOCX)

S1 Table Excluded full text articles. (DOCX)

S2 Table Extracted parameters from the data. (DOCX)

S3 Table Studies with both ML and regression-based models. (DOCX)

S4 Table Risk of bias assessment with the use of the PROBAST score. (DOCX)
