# Supplementary material for: Systematic evaluation of machine learning models for postoperative surgical site infection prediction
Source: PLoS One. 2024 Dec 12;19(12):e0312968. doi: 10.1371/journal.pone.0312968 (PMC11637340; doi:10.1371/journal.pone.0312968)
Supplement: S2 Appendix — (DOCX) [file pone.0312968.s002.docx]

# S2 Appendix. Search strategy

((("Prediction"[tw] OR "predict"[tw] OR "predictive"[tw] OR "Predictive Value of Tests"[Mesh] OR "Forecasting"[mesh] OR "Forecast*"[tw] OR "Clinical Decision Rules"[Mesh] OR "Clinical Decision Rule"[tw] OR "Clinical Decision Rules"[tw]) AND ("Decision Support Techniques"[Mesh] OR "Decision Support Systems, Clinical"[Mesh] "Decision support"[tw] OR "Clinical Decision Rules"[Mesh] OR "Decision Rule"[tw] "Decision rules"[tw] OR "Risk stratification"[tw] OR "Risk Assessment"[Mesh] OR "Risk assessment"[tw] OR "Risk Management"[Mesh] OR "Risk evaluation"[tw] OR "Stratification"[tw] OR "Artificial Intelligence"[Mesh] OR "Artificial Intelligence"[tw] OR "Machine Learning"[mesh] OR "Machine learning"[tw] OR "Computational Intelligence"[tw] OR "Computer Reasoning"[tw] OR "Machine Intelligence"[tw] OR "Computer Simulation"[Mesh] OR "Computer model"[tw] OR "Computer models"[tw] OR "Computer model*"[tw] OR "Models, Statistical"[mesh] OR "Statistical Model"[tw] OR "Statistical Models"[tw] OR "Statistical Model*"[tw] OR "Binomial Models"[tw] OR "Binomial Model"[tw] OR "Binomial Model*"[tw] OR "Polynomial Models"[tw] OR "Polynomial Model"[tw] OR "Polynomial Model*"[tw] OR "Computer Simulation"[tw] OR "Computerized Models"[tw] OR "Computerised Models"[tw] OR "Computerized Model"[tw] OR "Computerised Model"[tw] OR "Computerized Model*"[tw] OR "Computerised Model*"[tw] OR "Neural Networks, Computer"[mesh] OR "Computational Neural Networks"[tw] OR "Computational Neural Network"[tw] OR "Computer Neural Networks"[tw] OR "Computer Neural Network"[tw] OR "Connectionist Models"[tw] OR "Connectionist Model"[tw] OR "Connectionist Model*"[tw] OR "Neural Network Models"[tw] OR "Neural Network Model"[tw] OR "Neural Network Model*"[tw] OR "Patient-Specific Modeling"[tw] OR "Patient-Specific Model"[tw] OR "Patient-Specific Models"[tw] OR "Patient-Specific Computational Modeling"[tw] OR "Patient-Specific Computational Models"[tw] OR "Patient-Specific Computational Model"[tw] OR "Models, Theoretical"[Mesh] OR "Mathematical model"[tw] OR "Mathematical models"[tw] OR "Mathematical model*"[tw] OR "Theoretical model"[tw] OR "Theoretical models"[tw] OR "Theoretical model*"[tw] OR "Mathematical Computing"[Mesh] OR "Mathematic Computing"[tw] OR "Mathematical Computing"[tw] OR "Statistical Computing"[tw] OR "Computer Based Statistical Programs"[tw] OR "Computer Based Statistical Program"[tw] OR "Computer Based Statistical Program*"[tw] OR "Computer Heuristics"[mesh] OR "Computer Heuristics"[tw] OR "Software"[mesh] OR "Computer Programs"[tw] OR "Computer Program"[tw] OR "Computer Software"[tw] OR "Decision Making, Computer-Assisted"[mesh] OR "Computer-Assisted Decision Making"[tw] OR "Computer-Assisted Medical Decision Making"[tw] OR "Computer-Assisted Clinical Decision Making"[tw] OR "Decision Model"[tw] OR "Decision Models"[tw] OR "Decision Modeling"[tw] OR "Decision Support Model"[tw] OR "Decision Support Models"[tw] OR "Decision Support Modeling"[tw] OR "Classification model"[tw] OR "Classification models"[tw] OR "Classification model*"[tw] OR "model"[ti] OR "models"[ti] OR "model*"[ti] OR "Algorithms"[Mesh] OR "Algorithms"[tw] OR "Algorithm"[tw] OR "Algorithms"[tw]) AND ("postoperative infection"[tw] OR "postoperative infections"[tw] OR "postoperative infection*"[tw] OR "post operative infection"[tw] OR "post operative infections"[tw] OR "post operative infect*"[tw] OR "postoperative wound infection"[tw] OR "postoperative wound infections"[tw] OR "postoperative wound infection*"[tw] OR "post operative wound infection"[tw] OR "post operative wound infections"[tw] OR "post operative wound infect*"[tw] OR "Surgical Wound Infection"[Mesh] OR "Surgical Wound Infection"[tw] OR "Surgical Wound Infections"[tw] OR "Surgical Site Infection"[tw] OR "Surgical Site Infections"[tw] OR (("Postoperative Complications"[Mesh] OR "Postoperative Period"[Mesh] OR "Postoperative Care"[Mesh] OR "postoperative"[tw] OR "post operative"[tw] OR "postoperativ*"[tw] OR "post operativ*"[tw] OR "postsurgery"[tw] OR "post surgery"[tw] OR "postsurgical"[tw] OR "post surgical"[tw] OR "postsurgical*"[tw] OR "post surgical*"[tw] OR "after surgery"[tw] OR "after operation"[tw] OR "after procedure"[tw] OR "post procedures"[tw] OR "post procedure"[tw] OR "post procedural"[tw] OR "postprocedure"[tw] OR "postprocedures"[tw] OR "postprocedural"[tw]) AND ("Infections"[Mesh] OR "Infections"[tw] OR "Infection"[tw] OR "Infect*"[tw] OR "pneumonia"[tw] OR "pneumon*"[tw] OR "Anastomotic Leak"[Mesh] OR "Anastomotic Leak"[tw] OR "Anastomotic Leaks"[tw] OR "Anastomotic Leaking"[tw] OR "sepsis"[tw] OR "Bacteremia"[tw] OR "Candidemia"[tw] OR "Endotoxemia"[tw] OR "Fungemia"[tw] OR "Septicemia"[tw] OR "Parasitemia"[tw] OR "Septic Shock"[tw] OR "Viremia"[tw] OR "Bacteraemia"[tw] OR "Candidaemia"[tw] OR "Endotoxaemia"[tw] OR "Fungaemia"[tw] OR "Septicaemia"[tw] OR "Parasitaemia"[tw] OR "Viraemia"[tw] OR "Bacteremi*"[tw] OR "Candidemi*"[tw] OR "Endotoxemi*"[tw] OR "Fungemi*"[tw] OR "Septicemi*"[tw] OR "Parasitemi*"[tw] OR "Viremi*"[tw] OR "Bacteraemi*"[tw] OR "Candidaemi*"[tw] OR "Endotoxaemi*"[tw] OR "Fungaemi*"[tw] OR "Septicaemi*"[tw] OR "Parasitaemi*"[tw] OR "Viraemi*"[tw] OR "Inflammation"[Mesh] OR "Inflammation"[tw] OR "Acute-Phase Reaction"[tw] OR "Foreign-Body Reaction"[tw] OR "Implant Capsular Contracture"[tw] OR "Seroma"[tw] OR "Serositis"[tw] OR "Suppuration"[tw] OR "Abscess"[tw] OR "Cellulitis"[tw] OR "Empyema"[tw] OR "Systemic Inflammatory Response Syndrome"[tw] OR "Cytokine Release Syndrome"[tw])))) OR (("prediction model"[tw] OR "prediction models"[tw] OR "prediction model*"[tw] OR "predictive model"[tw] OR "predictive models"[tw] OR "predictive model*"[tw]) AND ("postoperative infection"[tw] OR "postoperative infections"[tw] OR "postoperative infection*"[tw] OR "post operative infection"[tw] OR "post operative infections"[tw] OR "post operative infect*"[tw] OR "postoperative wound infection"[tw] OR "postoperative wound infections"[tw] OR "postoperative wound infection*"[tw] OR "post operative wound infection"[tw] OR "post operative wound infections"[tw] OR "post operative wound infect*"[tw] OR "Surgical Wound Infection"[Mesh] OR "Surgical Wound Infection"[tw] OR "Surgical Wound Infections"[tw] OR "Surgical Site Infection"[tw] OR "Surgical Site Infections"[tw] OR (("Postoperative Complications"[Mesh] OR "Postoperative Period"[Mesh] OR "Postoperative Care"[Mesh] OR "postoperative"[tw] OR "post operative"[tw] OR "postoperativ*"[tw] OR "post operativ*"[tw] OR "postsurgery"[tw] OR "post surgery"[tw] OR "postsurgical"[tw] OR "post surgical"[tw] OR "postsurgical*"[tw] OR "post surgical*"[tw] OR "after surgery"[tw] OR "after operation"[tw] OR "after procedure"[tw] OR "post procedures"[tw] OR "post procedure"[tw] OR "post procedural"[tw] OR "postprocedure"[tw] OR "postprocedures"[tw] OR "postprocedural"[tw]) AND ("Infections"[Mesh] OR "Infections"[tw] OR "Infection"[tw] OR "Infect*"[tw] OR "pneumonia"[tw] OR "pneumon*"[tw] OR "Anastomotic Leak"[Mesh] OR "Anastomotic Leak"[tw] OR "Anastomotic Leaks"[tw] OR "Anastomotic Leaking"[tw] OR "sepsis"[tw] OR "Bacteremia"[tw] OR "Candidemia"[tw] OR "Endotoxemia"[tw] OR "Fungemia"[tw] OR "Septicemia"[tw] OR "Parasitemia"[tw] OR "Septic Shock"[tw] OR "Viremia"[tw] OR "Bacteraemia"[tw] OR "Candidaemia"[tw] OR "Endotoxaemia"[tw] OR "Fungaemia"[tw] OR "Septicaemia"[tw] OR "Parasitaemia"[tw] OR "Viraemia"[tw] OR "Bacteremi*"[tw] OR "Candidemi*"[tw] OR "Endotoxemi*"[tw] OR "Fungemi*"[tw] OR "Septicemi*"[tw] OR "Parasitemi*"[tw] OR "Viremi*"[tw] OR "Bacteraemi*"[tw] OR "Candidaemi*"[tw] OR "Endotoxaemi*"[tw] OR "Fungaemi*"[tw] OR "Septicaemi*"[tw] OR "Parasitaemi*"[tw] OR "Viraemi*"[tw] OR "Inflammation"[Mesh] OR "Inflammation"[tw] OR "Acute-Phase Reaction"[tw] OR "Foreign-Body Reaction"[tw] OR "Implant Capsular Contracture"[tw] OR "Seroma"[tw] OR "Serositis"[tw] OR "Suppuration"[tw] OR "Abscess"[tw] OR "Cellulitis"[tw] OR "Empyema"[tw] OR "Systemic Inflammatory Response Syndrome"[tw] OR "Cytokine Release Syndrome"[tw])))))
