# Supplementary material for: Systematic evaluation of machine learning models for postoperative surgical site infection prediction
Source: PLoS One. 2024 Dec 12;19(12):e0312968. doi: 10.1371/journal.pone.0312968 (PMC11637340; doi:10.1371/journal.pone.0312968)
Supplement: S1 Table — (DOCX) [file pone.0312968.s003.docx]

**Table S1. Excluded full text articles**

|  | Reference | Reason of exclusion |
| --- | --- | --- |
| 1. | Abd El Aziz, M.A., Perry, W.R., Grass, F. et al. Predicting primary postoperative pulmonary complications in patients undergoing minimally invasive surgery for colorectal cancer. Updates Surg 72, 977–983 (2020). <https://doi.org/10.1007/s13304-020-00892-6> | Wrong outcomes |
| 2. | Nicholas B. Abt, Rosh K. Sethi, Sidharth V. Puram, Mark A. Varvares, Preoperative laboratory data are associated with complications and surgical site infection in composite head and neck surgical resections,  American Journal of Otolaryngology, Volume 39, Issue 3, 2018  <https://doi.org/10.1016/j.amjoto.2018.01.017>. | Does not report (internal) validation performance metric(s) |
| 3. | Arsal Acarbaş, Value of N-Terminal Pro-Brain Natriuretic Peptide in Predicting Perioperative Complications Following Spine Surgery, World Neurosurgery, Volume 133, 2020  <https://doi.org/10.1016/j.wneu.2019.10.012>. | Does not report (internal) validation performance metric(s) |
| 4. | Ai J; Hu Y; Zhou FF; Liao YX; Yang T, Machine learning-assisted ensemble analysis for the prediction of urinary tract infection in elderly patients with ovarian cancer after cytoreductive surgery. World J Clin Oncol Dec 2022;13(12):967-979  <http://dx.doi.org/10.5306/wjco.v13.i12.967> | Timepoint infection not specified |
| 5. | Al Khatib, H. S.; Alramadhan, M.; Murphy, J.; Tsao, K.; Chang, M. L. Using artificial neural networks to predict intra-abdominal abscess risk post-appendectomy. Open Forum Infectious Diseases October 2019;6 (Supplement 2)():S842-S843. 2019 October  <https://doi.org/10.1093/ofid/ofz360.2116> | Conference abstract |
| 6. | Al Lawati Y; Alkaaki A; Ramírez García Luna JL; Skothos E; Mueller C; Spicer J; Mulder D; Ferri L; Cools-Lartigue J, The Predictive Value of Inflammatory Biomarkers in Esophageal Anastomotic Leaks. Ann Thorac Surg Jan 2021;(): Netherlands 2021 Jan  <https://dx.doi.org/10.1016/j.athoracsur.2020.12.033> | Timepoint infection not in prediction window |
| 7. | Alavi K; Sturrock PR; Sweeney WB; Maykel JA; Cervera-Servin JA; Tseng J; Cook EF, A simple risk score for predicting surgical site infections in inflammatory bowel disease. Dis Colon Rectum Nov 2010;53(11):1480-6 United States 2010 Nov  <https://dx.doi.org/10.1007/DCR.0b013e3181f1f0fd> | Timepoint infection not in prediction window |
| 8. | Alghamdi T; Abdel-Fattah M; Zautner A; Lorf T, Preoperative model for end-stage liver disease score as a predictor for posthemihepatectomy complications. Eur J Gastroenterol Hepatol Jun 2014;26(6):668-75. 2014 Jun  <https://dx.doi.org/10.1097/MEG.0000000000000035> | Wrong outcomes |
| 9. | Alkaz, A. V.; Cherkasov, A. U.; Vrabie, O.; Tkachenko, A. N., Prediction and prophylaxis of infectious complications of arthroscopic surgery for treatment of knee osteoarthritis. Osteoporosis International 2017;28 (Supplement 1)():S485 2017  <https://dx.doi.org/10.1007/s00198-017-3950-2> | Conference abstract |
| 10. | Allareddy, V.; Lee, M. K.; Vaid, N. R.; Yadav, S., Use of Neural Network model to examine post-operative infections following orthognathic surgeries in the United States, Seminars in Orthodontics Jun 2021;27(2):130-137, 2021 Jun  <https://dx.doi.org/10.1053/j.sodo.2021.05.009> | Timepoint infection not specified |
| 11. | Almeida AB; Faria G; Moreira H; Pinto-de-Sousa J; Correia-da-Silva P; Maia JC, Elevated serum C-reactive protein as a predictive factor for anastomotic leakage in colorectal surgery. Int J Surg 2012;10(2):87-91, United States 2012.  <https://dx.doi.org/10.1016/j.ijsu.2011.12.006> | Timepoint infection not specified |
| 12. | Amier Y; Zhang Y; Zhang J; Yao W; Wang S; Wei C; Yu X, Analysis of Preoperative Risk Factors for Postoperative Urosepsis After Mini-Percutaneous Nephrolithotomy in Patients with Large Kidney Stones. J Endourol Mar 2022;36(3):292-297. United States 2022 Mar.  <https://dx.doi.org/10.1089/end.2021.0406> | Timepoint infection not specified |
| 13. | Amroun K; Scholer V; Djerada Z; Renard Y; Bouche O; Rhaiem R; Kianmanesh R, Inflammatory biomarkers to predict postoperative infectious complications after cytoreductive surgery and HIPEC for peritoneal carcinomatosis. Eur J Surg Oncol Feb 2022;48(2):455-461, England 2022 Feb.  <https://dx.doi.org/10.1016/j.ejso.2021.09.015> | Timepoint infection not in prediction window |
| 14. | Anderson, B.; Resnick, E.; Waite, K.; Sanders, T.; Tafra, L.; Jackson, R. S. Evaluation of SSI risk prediction model for breast reconstruction outcomes, Annals of Surgical Oncology 2020;27 (Supplement 2)():S495, 2020  <https://dx.doi.org/10.1245/s10434-020-08630-3> | Conference abstract |
| 15. | Anderson, J. E.; Rose, J.; Noorbakhsh, A.; Talamini, M. A.; Finlayson, S. R.; Bickler, S. W.; Chang, D. C., An efficient risk adjustment model to predict inpatient adverse events after surgery, World J Surg Aug 2014;38(8):1954-60, 2014 Aug  <https://dx.doi.org/10.1007/s00268-014-2490-6> | Wrong outcomes |
| 16. | Andrade IN; Moraes Neto FR; Andrade TG, Use of EuroSCORE as a predictor of morbidity after cardiac surgery. Rev Bras Cir Cardiovasc Jan-Mar 2014;29(1):9-15, 2014 Jan-Mar.  <https://dx.doi.org/10.5935/1678-9741.20140005> | Timepoint infection not specified |
| 17. | Anteby, R.; Zager, Y.; Horesh, N.; Cordoba, M.; Russell, B.; Gutman, M.; Nevler, A.; Jacoby, H., The utility of naples prognostic score for predicting postoperative complications in Crohn's disease patients undergoing bowel resection, Diseases of the Colon and Rectum May 2021;64(5)():210-211, 2021 May.  <https://dx.doi.org/10.1097/DCR.0000000000002029> | Wrong outcomes |
| 18. | Arezzo A; Migliore M; Chiaro P; Arolfo S; Filippini C; Di Cuonzo D; Cirocchi R; Morino M, The REAL (REctal Anastomotic Leak) score for prediction of anastomotic leak after rectal cancer surgery. Tech Coloproctol Jul 2019;23(7):649-663, Italy 2019 Jul.  <https://dx.doi.org/10.1007/s10151-019-02028-4> | Timepoint infection not specified |
| 19. | Armstrong BN; Renson A; Zhao LC; Bjurlin MA, Development of novel prognostic models for predicting complications of urethroplasty. World J Urol Mar 2019;37(3):553-559. Germany 2019 Mar.  <https://dx.doi.org/10.1007/s00345-018-2413-5> | Wrong outcomes |
| 20. | Arshad, W.; Longbotham, D.; Kaur, G., Is P POSSUM better than anaesthetic/surgical assessment in risk stratification of the major surgical patient? Colorectal Disease July 2014;16():123, 2014 July.  <https://dx.doi.org/10.1111/codi.12644-1> | Conference abstract |
| 21. | Arvind, V.; Kim, J. S.; Oermann, E. K.; Kaji, D.; Cho, S. K., Predicting Surgical Complications in Adult Patients Undergoing Anterior Cervical Discectomy and Fusion Using Machine Learning, Neurospine Dec 2018;15(4):329-337, 2018 Dec.  <https://dx.doi.org/10.14245/ns.1836248.124> | Wrong outcomes |
| 22. | Asensio Vegas, A.; Monge Jodra, V.; Soriano, C.; López, R.; Gil, A.; Lizán García, M., Surgical wound infection: the risk factors and a predictive model, Med Clin (Barc) Apr 10 1993;100(14):521-5, 1993 Apr 10. | Not in English |
| 23. | Ashuvanth, S.; Anandhi, A.; Sureshkumar, S., Validation of ventral hernia risk score in predicting surgical site infections, Hernia Jan 20 2022;(): 2022 Jan 20.  <https://dx.doi.org/10.1007/s10029-021-02537-y> | Timepoint infection not in prediction window |
| 24. | Ausania F; Guzman Suarez S; Alvarez Garcia H; Senra del Rio P; Casal Nuñez E, Gallbladder perforation: morbidity, mortality and preoperative risk prediction. Surg Endosc Apr 2015;29(4):955-60, Germany 2015 Apr.  <https://dx.doi.org/10.1007/s00464-014-3765-6> | Wrong outcomes |
| 25. | Azimi K; Honaker MD; Chalil Madathil S; Khasawneh MT, Post-Operative Infection Prediction and Risk Factor Analysis in Colorectal Surgery Using Data Mining Techniques: A Pilot Study. Surg Infect (Larchmt) Nov 2020;21(9):784-792, United States 2020 Nov.  <https://dx.doi.org/10.1089/sur.2019.138> | Timepoint infection not specified |
| 26. | Aziz F; Bohr T; Lehman EB, Wound Disruption after Lower Extremity Bypass Surgery is a Predictor of Subsequent Development of Wound Infection. Ann Vasc Surg Aug 2017;43():176-187, Netherlands 2017 Aug.  <https://dx.doi.org/10.1016/j.avsg.2016.10.065> | Does not report (internal) validation performance metric(s) |
| 27. | Barbour JR; Iorio ML; Oh C; Tung TH; O'Neill PJ, Predictive Value of Nutritional Markers for Wound Healing Complications in Bariatric Patients Undergoing Panniculectomy. Ann Plast Surg Oct 2015;75(4):435-8, United States 2015 Oct.  <https://dx.doi.org/10.1097/SAP.0000000000000188> | Does not report (internal) validation performance metric(s) |
| 28. | Baron RB; Neifert SN; Ranson WA; Schupper AJ; Gal JS; Cho SK; Caridi JM, A Comparison of the Elixhauser and Charlson Comorbidity Indices: Predicting In-Hospital Complications Following Anterior Lumbar Interbody Fusions. World Neurosurg Dec 2020;144():e353-e360, United States 2020 Dec.  <https://dx.doi.org/10.1016/j.wneu.2020.08.138> | Timepoint infection not specified |
| 29. | Barry, T. M.; DeSantis, A. J.; Janjua, H. M.; Cousin-Peterson, E.; Saad, A.; Kuo, P., Predicting Post-Operative Clostridium Difficile Infection, Gastroenterology May 2020;158 (6 Supplement 1)():S-1482, 2020 May.  <https://dx.doi.org/10.1016/S0016-5085> | Conference abstract |
| 30. | Bekelis K; Bakhoum SF; Desai A; Mackenzie TA; Roberts DW, Outcome prediction in intracranial tumor surgery: the National Surgical Quality Improvement Program 2005-2010., J Neurooncol May 2013;113(1):57-64, 2013 May  <https://dx.doi.org/10.1007/s11060-013-1089-3> | Wrong outcomes |
| 31. | Bekelis K; Kalakoti P; Nanda A; Missios S, A Predictive Model of Unfavorable Outcomes After Benign Intracranial Tumor Resection. World Neurosurg Jul 2015;84(1):82-9, United States 2015 Jul.  <https://dx.doi.org/10.1016/j.wneu.2015.02.032> | Timepoint infection not specified |
| 32. | Bennett KM; Levinson H; Scarborough JE; Shortell CK, Validated prediction model for severe groin wound infection after lower extremity revascularization procedures. J Vasc Surg Feb 2016;63(2):414-9, United States 2016 Feb  <https://dx.doi.org/10.1016/j.jvs.2015.08.094> | Timepoint infection not specified |
| 33. | Beowsomboon, C.; Rajborirug, S.; Pruekrattananapa, Y.; Sangsuwan, T.; Jamulitrat, S., Poor predictive performance of the newnational healthcare safety network (NHSN) surgical site infection risk adjustment model for craniotomy,Antimicrobial Resistance and Infection Control. Conference: 8th International Congress of the Asia Pacific Society of Infection Control, APSIC 2017;6(Supplement 2): 2017  <https://dx.doi.org/10.1186/s13756-017-0176-1> | Conference abstract |
| 34. | Berdugina, V.; Berdugin, A., Acute pneumonia at endoprosthesis of large joints: Questions of forecasting, Osteoporosis International December 2020;31 (SUPPL 1)():S541-S542, 2020 December.  <https://dx.doi.org/10.1007/s00198-020-05696-3> | Conference abstract |
| 35. | Bermudez-Yera, G. D. J.; Naranjo-Ugalde, A. M.; Rabassa-LopezCallejas, M. A.; Lagomasino-Hidalgo, A. L.; Chaljub-Bravo, E.; Barreto-Fiu, E. E., Predictive model for postoperative mediastinitis after cardiovascular surgery, Cirugia Cardiovascular November - December 2019;26(6):277-282, 2019 November – December.  <https://dx.doi.org/10.1016/j.circv.2019.09.003> | Not in English |
| 36. | Bernardi K; Adrales GL; Hope WW; Keith J; Kuhlens H; Martindale RG; Melin AA; Orenstein SB; Roth JS; Shah SK; Tsuda S; Liang MK, Abdominal Wall Reconstruction Risk Stratification Tools: A Systematic Review of the Literature. Plast Reconstr Surg Sep 2018;142(3 Suppl):9S-20S, United States 2018 Sep.  <https://dx.doi.org/10.1097/PRS.0000000000004833> | Wrong intervention |
| 37. | Berríos-Torres SI; Mu Y; Edwards JR; Horan TC; Fridkin SK, Improved risk adjustment in public reporting: coronary artery bypass graft surgical site infections. Infect Control Hosp Epidemiol May 2012;33(5):463-9, United States 2012 May.  <https://dx.doi.org/10.1086/665313> | Timepoint infection not in prediction window |
| 38. | Betts KS; Kisely S; Alati R, Predicting common maternal postpartum complications: leveraging health administrative data and machine learning. BJOG May 2019;126(6):702-709, England 2019 May.  <https://dx.doi.org/10.1111/1471-0528.15607> | Timepoint infection not in prediction window |
| 39. | Bibby, B. A.; Collins, B. J.; Ayliffe, G. A., A mathematical model for assessing risk of postoperative wound infection, J Hosp Infect Jul 1986;8(1):31-8, 1986 Jul.  <https://dx.doi.org/10.1016/0195-6701(86)90102-7> | Timepoint infection not in prediction window |
| 40. | Bihorac A; Ozrazgat-Baslanti T; Ebadi A; Motaei A; Madkour M; Pardalos PM; Lipori G; Hogan WR; Efron PA; Moore F; Moldawer LL; Wang DZ; Hobson CE; Rashidi P; Li X; Momcilovic P, MySurgeryRisk: Development and Validation of a Machine-learning Risk Algorithm for Major Complications and Death After Surgery. Ann Surg Apr 2019;269(4):652-662, 2019 Apr.  <https://dx.doi.org/10.1097/SLA.0000000000002706> | Timepoint infection not specified |
| 41. | Birnie DH; Wang J; Alings M; Philippon F; Parkash R; Manlucu J; Angaran P; Rinne C; Coutu B; Low RA; Essebag V; Morillo C; Redfearn D; Toal S; Becker G; Degrâce M; Thibault B; Crystal E; Tung S; LeMaitre J; Sultan O; Bennett M; Bashir J; Ayala-Paredes F; Gervais P; Rioux L; Hemels MEW; Bouwels LHR; Exner DV; Dorian P; Connolly SJ; Longtin Y; Krahn AD, Risk Factors for Infections Involving Cardiac Implanted Electronic Devices. J Am Coll Cardiol Dec 2019;74(23):2845-2854, United States 2019 Dec.  <https://dx.doi.org/10.1016/j.jacc.2019.09.060> | Timepoint infection not in prediction window |
| 42. | Biscione FM; Couto RC; Pedrosa TM, Performance, revision, and extension of the National Nosocomial Infections Surveillance system's risk index in Brazilian hospitals. Infect Control Hosp Epidemiol Feb 2012;33(2):124-34, United States 2012 Feb.  <https://dx.doi.org/10.1086/663702> | Does not report (internal) validation performance metric(s) |
| 43. | Blair, B. M.; Lehman, E. B.; Jafri, S. M.; Kaag, M. G.; Raman, J. D., Predicted versus observed 30-day perioperative outcomes using the ACS NSQIP surgical risk calculator in patients undergoing partial nephrectomy for renal cell carcinoma, Int Urol Nephrol Jul 2018;50(7):1249-1256, 2018 Jul.  <https://dx.doi.org/10.1007/s11255-018-1898-6> | Wrong outcomes |
| 44. | Blatnik JA; Krpata DM; Novitsky YW; Rosen MJ, Does a history of wound infection predict postoperative surgical site infection after ventral hernia repair? Am J Surg Mar 2012;203(3):370-4; discussion 374, United States 2012 Mar.  <https://dx.doi.org/10.1016/j.amjsurg.2011.12.001> | Does not report (internal) validation performance metric(s) |
| 45. | Boaro A; Wells M; Chi J; Lu Y; Smith TR; Groff MW; Zaidi H, A National Surgical Quality Improvement Program Analysis of Postoperative Major and Minor Complications in Patients with Spinal Metastatic Disease. World Neurosurg Aug 2020;140():e203-e211, United States 2020 Aug.  <https://dx.doi.org/10.1016/j.wneu.2020.04.225> | Wrong outcomes |
| 46. | Bolat D; Topcu YK; Aydogdu O; Minareci S; Dincel C, Neutrophil to Lymphocyte Ratio as a predictor of early penile prosthesis implant infection. Int Urol Nephrol Jun 2017;49(6):947-953, Netherlands 2017 Jun.  <https://dx.doi.org/10.1007/s11255-017-1569-z> | Timepoint infection not in prediction window |
| 47. | Bona D; Micheletto G; Bonitta G; Panizzo V; Cavalli M; Rausa E; Cirri S; Aiolfi A, Does C-reactive Protein Have a Predictive Role in the Early Diagnosis of Postoperative Complications After Bariatric Surgery? Systematic Review and Bayesian Meta-analysis. Obes Surg Nov 2019;29(11):3448-3456, United States 2019 Nov.  <https://dx.doi.org/10.1007/s11695-019-04013-0> | Timepoint infection not specified |
| 48. | Boubekki, A.; Nordhaugmyhre, J.; Luppino, L. T.; yvind Mikalsen, K.; Revhaug, A.; Jenssen, R., Clinically relevant features for predicting the severity of surgical site infections, IEEE Journal of Biomedical and Health Informatics. 2021;(): 2021.  <https://dx.doi.org/10.1109/JBHI.2021.3121038> | Does not report (internal) validation performance metric(s) |
| 49. | Boyd, S. S.; O'Sullivan, D. M.; Lasala, C., Evaluating Postoperative Morbidity in Patients Undergoing Pelvic Reconstructive Surgery Using the American College of Surgeons National Surgical Quality Improvement Program Surgical Risk Calculator. Female Pelvic Med Reconstr Surg Jun 2020;26(6):364-369, 2020 Jun.  <https://dx.doi.org/10.1097/spv.0000000000000715> | Wrong outcomes |
| 50. | Brennan, M.; Puri, S.; Ozrazgat-Baslanti, T.; Momcilovic, P.; Wang, D. Z.; Bihorac, A., Improving preoperative risk assessment: A pilot study comparing physician clinical judgement with computer algorithms, Anesthesia and Analgesia April 2018;126 (4 Supplement 1)():746,  2018 April | Conference abstract |
| 51. | Broda A; Sanford Z; Turcotte J; Patton C, Development of a Risk Prediction Model With Improved Clinical Utility in Elective Cervical and Lumbar Spine Surgery. Spine (Phila Pa 1976) May 2020;45(9):E542-E551, United States 2020 May.  <https://dx.doi.org/10.1097/BRS.0000000000003317> | Wrong outcomes |
| 52. | Brooks-Brunn, J. A., Validation of a predictive model for postoperative pulmonary complications. Heart & Lung May-Jun 1998;27(3):151-158, 1998 May-Jun.  <https://dx.doi.org/10.1016/s0147-9563(98)90002-4> | Wrong outcomes |
| 53. | Bucher, B. T.; Skarda, D. E.; Finlayson, S. R. G.; Chapman, W. W.; Gundlapalli, A. V.; Ferraro, J. P., Bayesian Networks for Detection of Postoperative Health Care-Associated Infections Using Electronic Health Care Record Data, Journal of the American College of Surgeons October 2019;229 (4 Supplement 2)():e27, 2019 October.  <https://dx.doi.org/10.1016/j.jamcollsurg.2019.08.823> | Conference abstract |
| 54. | Bunn C; Kulshrestha S; Boyda J; Balasubramanian N; Birch S; Karabayir I; Baker M; Luchette F; Modave F; Akbilgic O, Application of machine learning to the prediction of postoperative sepsis after appendectomy. Surgery Mar 2021;169(3):671-677, 2021 Mar.  <https://dx.doi.org/10.1016/j.surg.2020.07.045> | Wrong patient population |
| 55. | Bures, C.; Klatte, T.; Gilhofer, M.; Behnke, M.; Breier, A. C.; Neuhold, N.; Hermann, M., A prospective study on surgical site infections in thyroid surgery. Langenbeck's Archives of Surgery October 2013;398 (7)():1009, 2013 October.  <https://dx.doi.org/10.1007/s00423-013-1109-y> | Conference abstract |
| 56. | Bures C; Klatte T; Gilhofer M; Behnke M; Breier AC; Neuhold N; Hermann M, A prospective study on surgical-site infections in thyroid operation. Surgery Apr 2014;155(4):675-81, United States 2014 Apr.  <https://dx.doi.org/10.1016/j.surg.2013.12.002> | Timepoint infection not in prediction window |
| 57. | Burgess JR; Smith B; Britt R; Weireter L; Polk T, Predicting Postoperative Complications for Acute Care Surgery Patients Using the ACS NSQIP Surgical Risk Calculator. Am Surg Jul 2017;83(7):733-738, United States 2017 Jul. | Timepoint infection not in prediction window |
| 58. | Burke, J.; Rattan, R.; Sedighim, S.; Kim, M., A Simple Risk Score to Predict Clavien-Dindo Grade IV and V Complications After Non-elective Cholecystectomy, Journal of Gastrointestinal Surgery 2020;(): 2020.  <https://dx.doi.org/10.1007/s11605-020-04514-9> | Wrong outcomes |
| 59. | Bustamante-Munguira J; Herrera-Gómez F; Ruiz-Álvarez M; Figuerola-Tejerina A; Hernández-Aceituno A, A New Surgical Site Infection Risk Score: Infection Risk Index in Cardiac Surgery. J Clin Med Apr 2019;8(4): 2019 Apr.  <https://dx.doi.org/10.3390/jcm8040480> | Does not report (internal) validation performance metric(s) |
| 60. | Byrne, M. E.; Abu-Rustum, N. R.; Usiak, S. C.; Frame, J. W.; Aslam, A.; Ogden, S.; Leitao, M. M.; Sonoda, Y.; Zivanovic, O., Risk prediction model for surgical site infections in patients undergoing open gynecologic cancer surgery following the implementation of a reduction bundle at a comprehensive cancer center. Gynecologic Oncology Jun 2018;149():215-215, 2018 Jun.  <https://dx.doi.org/10.1016/j.ygyno.2018.04.489> | Conference abstract |
| 61. | Cadillo-Chavez, R.; de Echegaray, S.; Santiago-Delpin, E. A.; Rodriguez-Trinidad, A. T.; Camacho-Carrazo, B.; Alfaro, T.; Saavedra-Pozo, M.; Carrasquillo, L.; Gonzalez-Caraballo, Z. A.; Morales-Otero, L. A., Assessing the Risk of Infection and Rejection in Hispanic Renal Transplant Recipients by Means of an Adenosine Triphosphate Release Assay. Transplantation Proceedings April 2006;38(3):918-920, 2006 April.  <https://dx.doi.org/10.1016/j.transproceed.2006.02.051> | Does not report (internal) validation performance metric(s) |
| 62. | Cagigas Fernández, C.; Palazuelos, C.; Cristobal Poch, L.; Gomez Ruiz, M., A probabilistic model for the prediction of intra-abdominal infection after colorectal surgery. Int J Colorectal Dis Nov 2021;36(11):2481-2488, 2021 Nov.  <https://dx.doi.org/10.1007/s00384-021-03955-1> | Timepoint infection not specified |
| 63. | Calderón-Parra J; Sánchez-Chica E; Asensio-Vegas Á; Fernández-Lozano I; Toquero-Ramos J; Castro-Urda V; Royuela-Vicente A; Ramos-Martínez A, Proposal for a Novel Score to Determine the Risk of Cardiac Implantable Electronic Device Infection. Rev Esp Cardiol (Engl Ed) Oct 2019;72(10):806-812, Spain 2019 Oct.  <https://dx.doi.org/10.1016/j.rec.2018.09.003> | Timepoint infection not in prediction window |
| 64. | Carranza-Lira S; Serrano-Estrada FD; López-Muñoz E; Hernández-Jiménez LM; Chavarría-Olarte ME, Glycosylated hemoglobin level in patients with and without surgical site infection after hysterectomy. Cir Cir 2020;88(3):344-348, Mexico 2020.  <https://dx.doi.org/10.24875/CIRU.19001411> | Not in English |
| 65. | Castro, M.; Espitia, E. L.; Tarazona, N.; Dominguez, L. C., Evaluation of surgical apagar scale in the prediction of postoperative complications and mortality: A prospective analysis in a fourth level center in bogota. Journal of Surgical Research. Conference: 8th Annual Academic Surgical Congress of the Association for Academic Surgery, AAS and the Society of University Surgeons, SUS. New Orleans, LA United States. Conference Publication: 2013;179(2): 2013. | Conference abstract |
| 66. | Cevenini G; Barbini E; Scolletta S; Biagioli B; Giomarelli P; Barbini P, A comparative analysis of predictive models of morbidity in intensive care unit after cardiac surgery - part II: an illustrative example. BMC Med Inform Decis Mak Nov 2007;7():36, 2007 Nov.  <https://dx.doi.org/10.1186/1472-6947-7-36> | Wrong outcomes |
| 67. | Chalmers BP; Kapadia M; Chiu YF; Miller AO; Henry MW; Lyman S; Carli AV, Accuracy of Predictive Algorithms in Total Hip and Knee Arthroplasty Acute Periprosthetic Joint Infections Treated With Debridement, Antibiotics, and Implant Retention (DAIR). J Arthroplasty Feb 2021;(): United States 2021 Feb.  <https://dx.doi.org/10.1016/j.arth.2021.02.039> | Timepoint infection not in prediction window |
| 68. | Chaochankit W; Samphao S; Mahattanobon S; Sungworawongpana C, Clinical Predictive Score for Cholecystectomy Wound Infection: WEBAC Score. J Gastrointest Surg Jun 2023;(): United States 2023 Jun.  <https://dx.doi.org/10.1007/s11605-023-05750-5> | Does not report (internal) validation performance metric(s) |
| 69. | Chapman G; Holton J; Chapman A, A threshold for concern? C-reactive protein levels following operatively managed neck of femur fractures can detect infectious complications with a simple formula. Clin Biochem Feb 2016;49(3):219-24, United States 2016 Feb.  <https://dx.doi.org/10.1016/j.clinbiochem.2015.10.018> | Timepoint infection not in prediction window |
| 70. | Chen C; Wen T; Zhao Q, The Change of Laboratory Tests Could Be Predictive Factors for Infection after McKeown Esophagogastrectomy. Biomed Res Int 2019;2019():9718705, 2019.  <https://dx.doi.org/10.1155/2019/9718705> | Does not report (internal) validation performance metric(s) |
| 71. | Chen C; Yang D; Gao S; Zhang Y; Chen L; Wang B; Mo Z; Yang Y; Hei Z; Zhou S, Development and performance assessment of novel machine learning models to predict pneumonia after liver transplantation. Respir Res Mar 2021;22(1):94, 2021 Mar.  <https://dx.doi.org/10.1186/s12931-021-01690-3> | Timepoint infection not in prediction window |
| 72. | Chen KA; Joisa CU; Stem JM; Guillem JG; Gomez SM; Kapadia MR, Improved Prediction of Surgical-Site Infection After Colorectal Surgery Using Machine Learning, Dis Colon Rectum Mar 2023;66(3):458-466, United States 2023 Mar.  <https://dx.doi.org/10.1097/DCR.0000000000002559> | Timepoint infection not specified |
| 73. | Chen L; Liu C; Ye Z; Huang S; Liang T; Li H; Chen J; Chen W; Guo H; Chen T; Yao Y; Jiang J; Sun X; Yi M; Liao S; Yu C; Wu S; Fan B; Zhan X, Predicting Surgical Site Infection Risk after Spinal Tuberculosis Surgery: Development and Validation of a Nomogram. Surg Infect (Larchmt) Aug 2022;23(6):564-575, United States 2022 Aug.  <https://dx.doi.org/10.1089/sur.2022.042> | Timepoint infection not specified |
| 74. | Chen Y; Lian BQ; Peng L; Ding CY; Lin YX; Yu LH; Wang DL; Kang DZ, Neutrophil to lymphocyte ratio is a prognosis factor for post-operative pneumonia in aneurysmal subarachnoid hemorrhage patients. Chin Med J (Engl) Dec 2020;134(6):682-689, 2020 Dec.  <https://dx.doi.org/10.1097/CM9.0000000000001304> | Does not report (internal) validation performance metric(s) |
| 75. | Cheney, C. P.; Crispin, A.; Mansmann, U., The value of clinical variables beyond administrative variables for predicting adverse outcomes in patients undergoing surgery for colorectal cancer. Diseases of the Colon and Rectum June 2020;63 (6)():e327, 2020 June.  <https://dx.doi.org/10.1097/DCR.0000000000001712> | Conference abstract |
| 76. | Cheng, S.; He, B.; Zeng, X., Prediction of anastomotic leakage after anterior rectal resection , Pakistan Journal of Medical Sciences May-June 2019;35(3):830-835, 2019 May-June.  <https://dx.doi.org/10.12669/pjms.35.3.252> | Timepoint infection not specified |
| 77. | Choi JY; Kim JK; Kim KI; Lee YK; Koo KH; Kim CH, How does the multidimensional frailty score compare with grip strength for predicting outcomes after hip fracture surgery in older patients? A retrospective cohort study. BMC Geriatr Apr 2021;21(1):234, 2021 Apr.  <https://dx.doi.org/10.1186/s12877-021-02150-9> | Wrong outcomes |
| 78. | Chrastil J; Anderson MB; Stevens V; Anand R; Peters CL; Pelt CE, Is Hemoglobin A1c or Perioperative Hyperglycemia Predictive of Periprosthetic Joint Infection or Death Following Primary Total Joint Arthroplasty? J Arthroplasty Jul 2015;30(7):1197-202, United States 2015 Jul.  <https://dx.doi.org/10.1016/j.arth.2015.01.040> | Does not report (internal) validation performance metric(s) |
| 79. | Clements AC; Tong EN; Morton AP; Whitby M, Risk stratification for surgical site infections in Australia: evaluation of the US National Nosocomial Infection Surveillance risk index. J Hosp Infect Jun 2007;66(2):148-55, England 2007 Jun.  <https://dx.doi.org/10.1016/j.jhin.2007.02.019> | Timepoint infection not specified |
| 80. | Colavita, P. D.; Coakley, K. M.; Heniford, D. W.; Bradley, J. F.; Prasad, T.; Lincourt, A. E.; Carbonell, A. M.; Cobb, W. S.; Augenstein, V. A.; Heniford, B. T., External validation of a clinical prediction tool for wound infection in open ventral hernia repair (OVHR), Hernia May 2017;21 (2 Supplement 1)():S199, 2017 May.  <https://dx.doi.org/10.1007/s10029-017-1595-x> | Conference abstract |
| 81. | Colborn KL; Bronsert M; Hammermeister K; Henderson WG; Singh AB; Meguid RA, Identification of urinary tract infections using electronic health record data. Am J Infect Control Apr 2019;47(4):371-375, 2019 Apr.  <https://dx.doi.org/10.1016/j.ajic.2018.10.009> | Wrong outcomes |
| 82. | Cole RR; Robbins KT; Cohen JI; Wolf PF, A predictive model for wound sepsis in oncologic surgery of the head and neck. Otolaryngol Head Neck Surg Feb 1987;96(2):165-71, England 1987 Feb.  <https://dx.doi.org/10.1177/019459988709600209> | Timepoint infection not specified |
| 83. | Copeland-Halperin LR; Emery E; Collins D; Liu C; Dort J, Dogma without Data: A Clinical Decision-Making Tool for Postoperative Blood Cultures. Am Surg Aug 2018;84(8):1339-1344, United States 2018 Aug. | Timepoint infection not in prediction window |
| 84. | Crawford, J.; Tagney, J.; Albarran, J. W., The Brompton and Harefield surgical site infectionrisk stratification score: Does it predict increasedrisk of surgical site infections in a cardiac surgerypopulation? European Journal of Cardiovascular Nursing May 2017;16 (SUPPL 1)():S52, 2017 May.  <https://dx.doi.org/10.1177/1474515117700580> | Conference abstract |
| 85. | da Silva DA; Ten Caten CS; Dos Santos RP; Fogliatto FS; Hsuan J, Predicting the occurrence of surgical site infections using text mining and machine learning. PLoS One 2019;14(12):e0226272, 2019.  <https://dx.doi.org/10.1371/journal.pone.0226272> | Wrong patient population |
| 86. | Dang, J. T.; Tran, C.; Delisle, M.; Switzer, N.; Laffin, M.; Madsen, K.; Birch, D.; Karmali, S., Surgical site infections following laparoscopic bariatric surgery: Development of the bariwound tool using the mbsaqip database. Surgical Endoscopy April 2019;33 (Supplement 1)():S26, 2019 April.  <https://dx.doi.org/10.1007/s00464-019-06703-3> | Wrong outcomes |
| 87. | Dang JT; Tran C; Switzer N; Delisle M; Laffin M; Madsen K; Birch DW; Karmali S, Predicting surgical site infections following laparoscopic bariatric surgery: development of the BariWound tool using the MBSAQIP database. Surg Endosc Apr 2020;34(4):1802-1811, Germany 2020 Apr.  <https://dx.doi.org/10.1007/s00464-019-06932-6> | Duplicate (exact similar publication in other journal) |
| 88. | de Campos-Lobato LF; Wells B; Wick E; Pronty K; Kiran R; Remzi F; Vogel JD, Predicting organ space surgical site infection with a nomogram. J Gastrointest Surg Nov 2009;13(11):1986-92, United States 2009 Nov.  <https://dx.doi.org/10.1007/s11605-009-0968-6> | Timepoint infection not in prediction window |
| 89. | de Godoy MF; Takakura IT; Correa PR; Machado MN; Miranda RC; Brandi AC, Preoperative nonlinear behavior in heart rate variability predicts morbidity and mortality after coronary artery bypass graft surgery. Med Sci Monit Mar 2009;15(3):CR117-22, United States 2009 Mar. | Wrong outcomes |
| 90. | De Miguel, M.; Cunha, M.; Salvans, S.; Alonso, S.; Jimenez, M.; Pascual, M.; Pera, M., Can albumin predict the risk of anastomotic leakage in patients undergoing colonic or intestinal resection? Colorectal Disease September 2019;21 (Supplement 3)():47, 2019 September. | Conference abstract |
| 91. | de Oliveira AC; Ciosak SI; Ferraz EM; Grinbaum RS, Surgical site infection in patients submitted to digestive surgery: risk prediction and the NNIS risk index. Am J Infect Control May 2006;34(4):201-7, United States 2006 May.  <https://dx.doi.org/10.1016/j.ajic.2005.12.011> | Wrong patient population |
| 92. | De Souza, F. H. B.; Couto, B. R. G. M.; Da Conceicao, F. L. A.; Da Silva, G. H. S.; Dias, I. G.; Rigueira, R. V. M.; Pimenta, G. M.; Martins, M. B.; Mendes, J. C. O.; Januario, G. B.; Oliveira, R. T.; De Vasconcelos, L. F.; De Araujo, L. L.; Rodrigues, A. C. R.; E. Silva C.M.O; De Souza, E. V.; Melo, J. F.; De Sa, M. C. A.; Silva, W. M.; Brant, B. A.; Carneiro, F. C. B.; Ferreira, M. L. F. P.; De Abreu Oliveira, N. T.; De Almeida Costa, N.; De Carvalho Tonaco, V., Technology and Medicine: Prediction of Surgical Site Infection in Clean Surgeries using Artificial Neural Networks. Open Forum Infectious Diseases October 2020;7 (SUPPL 1)():S487, 2020 October.  <https://dx.doi.org/10.1093/ofid/ofaa439.1095> | Conference abstract |
| 93. | De Souza, F. H. B.; Couto, B. R. G. M.; Da Conceicao, F. L. A.; Da Silva, G. H. S.; Dias, I. G.; Rigueira, R. V. M.; Pimenta, G. M.; Martins, M. B.; Mendes, J. C. O.; Januario, G. B.; Oliveira, R. T.; De Vasconcelos, L. F.; De Araujo, L. L.; De Freitas, C. M.; De Carvalho, J. M.; Alves, L. T.; Melo, L. G. C.; Freitas, S. F.; Guerra, S. A.; Rodrigues, A. C. R.; E. Silva C.M.O; De Souza, E. V.; Melo, J. F.; De Sa, M. C. A.; Silva, W. M., Risk prediction for surgical site infection in patients subject to knee arthroplasty surgery, Open Forum Infectious Diseases October 2020;7 (SUPPL 1)():S484-S485, 2020 October.  <https://dx.doi.org/10.1093/ofid/ofaa439.1089> | Conference abstract |
| 94. | De Souza, F. H. B.; Couto, B. R. G. M.; Da Conceicao, F. L. A.; Da Silva, G. H. S.; Dias, I. G.; Rigueira, R. V. M.; Pimenta, G. M.; Martins, M. B.; Mendes, J. C. O.; Januario, G. B.; Oliveira, R. T.; De Vasconcelos, L. F.; De Araujo, L. L.; Rodrigues, A. C. R.; Oliveira, E. Silva C. M.; De Souza, E. V.; Melo, J. F.; Assuncao De Sa, M. C.; Silva, W. M.; Barbosa, A. V. A.; Talim, A. T.; Alcasar, L. D.; Avelar, L. M.; Neto, M. B. P. O.; Santos, P. A. P.; Porto, V. S., Prediction of surgical site infection risk in patients undergoing bariatric surgery, Open Forum Infectious Diseases October 2020;7 (SUPPL 1)():S482-S483, 2020 October.  <https://dx.doi.org/10.1093/ofid/ofaa439.1084> | Conference abstract |
| 95. | De Souza, F. H. B.; Marinho Couto, B. R. G.; Da Conceicao, F. L. A.; Da Silva, G. H. S.; Dias, I. G.; Rigueira, R. V. M.; Pimenta, G. M.; Martins, M. B.; Mendes, J. C. O.; Januario, G. B.; Oliveira, R. T.; De Vasconcelos, L. F.; De Araujo, L. L.; Rodrigues, A. C. R.; E. Silva C.M.O; De Souza, E. V.; Melo, J. F.; De Sa, M. C. A.; Silva, W. M.; Barnabe, F. A. P.; Cunha, B. S.; Lemos, J. F.; Da Fonseca, L. R.; Junior, L. C. V.; De Pinho, V. B., Prediction of surgical site infections in colon surgery in belo horizonte hospitals. Open Forum Infectious Diseases October 2020;7 (SUPPL 1)():S483, 2020 October.  <https://dx.doi.org/10.1093/ofid/ofaa439.1085> | Conference abstract |
| 96. | De Souza, F. H. B.; Marinho Couto, B. R. G.; Da Conceicao, F. L. A.; Da Silva, G. H. S.; Dias, I. G.; Rigueira, R. V. M.; Pimenta, G. M.; Martins, M. B.; Mendes, J. C. O.; Januario, G. B.; Oliveira, R. T.; De Vasconcelos, L. F.; De Araujo, L. L.; Rodrigues, A. C. R.; E. Silva C.M.O; De Souza, E. V.; Melo, J. F.; De Sa, M. C. A.; Silva, W. M.; Lopes, A. L. A.; Filho, D. J. M.; Gatti, J. C.; Mol, L. M. O.; Santana, M. P. D.; Souto, M. V.; Vieira, P., Prediction of occurrence for surgical site infection in infected surgeries, Open Forum Infectious Diseases October 2020;7 (SUPPL 1)():S482, 2020 October.  <https://dx.doi.org/10.1093/ofid/ofaa439.1083> | Conference abstract |
| 97. | De Souza, F. H. B.; Marinho Couto, B. R. G.; Da Conceicao, F. L. A.; Da Silva, G. H. S.; Dias, I. G.; Rigueira, R. V. M.; Pimenta, G. M.; Martins, M. B.; Mendes, J. C. O.; Januario, G. B.; Oliveira, R. T.; De Vasconcelos, L. F.; De Araujo, L. L.; Rodrigues, A. C. R.; E. Silva C.M.O; De Souza, E. V.; Melo, J. F.; Assuncao De Sa, M. C.; Silva, W. M.; De Souza Alves, B. I.; Resende, D. M.; Borges, G. P. A.; Santos, G. R. M.; Santos Xavier, J. P. S.; Lacerda, L. D. P.; Silva, M. J. S., Occurrence's prediction of surgical site infection in limb amputation surgery, Open Forum Infectious Diseases October 2020;7 (SUPPL 1)():S481, 2020 October.  <https://dx.doi.org/10.1093/ofid/ofaa439.1081> | Conference abstract |
| 98. | De Souza, F. H. B.; Marinho Couto, B. R. G.; Da Conceicao, F. L. A.; Da Silva, G. H. S.; Dias, I. G.; Rigueira, R. V. M.; Pimenta, G. M.; Martins, M. B.; Mendes, J. C. O.; Januario, G. B.; Oliveira, R. T.; De Vasconcelos, L. F.; De Araujo, L. L.; Rodrigues, A. C. R.; E. Silva C.M.O; De Souza, E. V.; Melo, J. F.; Assuncao De Sa, M. C.; Silva, W. M.; Araujo, A. L. P.; Magalhaes, B. B.; Caetano, C. V.; Dias Guerra, C. M.; Braga, G. L., Artificial neural networks to predict surgical site infection in aorta artery aneurysm correction, Open Forum Infectious Diseases October 2020;7 (SUPPL 1)():S476-S477, 2020 October.  <https://dx.doi.org/10.1093/ofid/ofaa439.1068> | Conference abstract |
| 99. | De Souza, F. H. B.; Marinho Couto, B. R. G.; Da Conceicao, F. L. A.; Silvestre da Silva, G. H.; Dias, I. G.; Rigueira, R. V. M.; Pimenta, G. M.; Martins, M. B.; Mendes, J. C. O.; Januario, G. B.; Oliveira, R. T.; De Vasconcelos, L. F.; De Araujo, L. L.; Rodrigues, A. C. R.; E. Silva C.M.O; De Souza, E. V.; Melo, J. F.; Assuncao De Sa, M. C.; Silva, W. M.; Bastos, B. B.; Teles, D. G.; Barrancos, J. V.; De Souza Junqueira, J. T.; Garcia, L. V.; Brito, M. T. A., Artificial neural networks to predict infection in the surgical site in patients over 70 years old, Open Forum Infectious Diseases October 2020;7 (SUPPL 1)():S476, 2020 October.  <https://dx.doi.org/10.1093/ofid/ofaa439.1067> | Conference abstract |
| 100. | Del Toro MD; Peñas C; Conde-Albarracín A; Palomino J; Brun F; Sánchez S; Rodríguez-Baño J, Development and validation of baseline, perioperative and at-discharge predictive models for postsurgical prosthetic joint infection. Clin Microbiol Infect Feb 2019;25(2):196-202, England 2019 Feb.  <https://dx.doi.org/10.1016/j.cmi.2018.04.023> | Timepoint infection not in prediction window |
| 101. | Delgado-Rodríguez M; Palma S; Gómez-Ortega A; Martínez-Gallego G; Medina-Cuadros M, Indices of surgical site infection risk and prediction of other adverse outcomes during hospitalization. Infect Control Hosp Epidemiol Aug 2006;27(8):825-8, United States 2006 Aug.  <https://dx.doi.org/10.1086/506402> | Timepoint infection not in prediction window |
| 102. | DeLuzio MR; Keshava HB; Wang Z; Boffa DJ; Detterbeck FC; Kim AW, A model for predicting prolonged length of stay in patients undergoing anatomical lung resection: a National Surgical Quality Improvement Program (NSQIP) database study. Interact Cardiovasc Thorac Surg Aug 2016;23(2):208-15, England 2016 Aug.  <https://dx.doi.org/10.1093/icvts/ivw090> | Wrong outcomes |
| 103. | den Dulk, M.; Witvliet, M. J.; Kortram, K.; Neijenhuis, P. A.; de Hingh, I. H.; Engel, A. F.; van de Velde, C. J.; de Brauw, L. M.; Putter, H.; Brouwers, M. A.; Steup, W. H., The DULK (Dutch leakage) and modified DULK score compared: actively seek the leak. Colorectal Dis Sep 2013;15(9):e528-33, 2013 Sep.  <https://dx.doi.org/10.1111/codi.12379> | Timepoint infection not specified |
| 104. | Dente CJ; Bradley M; Schobel S; Gaucher B; Buchman T; Kirk AD; Elster E. Towards precision medicine: Accurate predictive modeling of infectious complications in combat casualties. J Trauma Acute Care Surg Oct 2017;83(4):609-616, United States 2017 Oct.  <https://dx.doi.org/10.1097/TA.0000000000001596> | Timepoint infection not specified |
| 105. | Diaz-Perez, D. E.; Laso-Garcia, I.; Sanchez-Guerrero, C.; Dominguez-Gutierrez, A.; Duque-Ruiz, G.; Ruiz-Hernandez, M.; Arias-Funez, F.; Burgos-Revilla, F. J., Sepsis after ureterorenoscopy: Development of a predictive model, European Urology, Supplements 2019;18 (7)():e2761, 2019.  <https://dx.doi.org/10.1016/S1569-9056> | Conference abstract |
| 106. | Dib F; Parenti LR; Boutten A; Hajage D; Marmuse JP, Diagnostic Performance of C-Reactive Protein in Detecting Post-Operative Infectious Complications After Laparoscopic Sleeve Gastrectomy. Obes Surg Dec 2017;27(12):3124-3132, United States 2017 Dec.  <https://dx.doi.org/10.1007/s11695-017-2744-0> | Wrong outcomes |
| 107. | Dietch, Z.; Guidry, C.; Davies, S.; Shah, P. M.; Sawyer, R., Machine learning and cloud computing enable enhanced prediction of surgical site infection, Surgical Infections May 2016;17 (Supplement 1)():S29, 2016 May.  <https://dx.doi.org/10.1089/sur.2016.29005.abstracts> | Conference abstract |
| 108. | Dietz N; Sharma M; Alhourani A; Ugiliweneza B; Wang D; Drazin D; Boakye M, Evaluation of Predictive Models for Complications following Spinal Surgery. J Neurol Surg A Cent Eur Neurosurg Nov 2020;81(6):535-545, Germany 2020 Nov.  <https://dx.doi.org/10.1055/s-0040-1709709> | Timepoint infection not in prediction window |
| 109. | Ding Z; Wang X; Jiang S; Liu J, Risk factors for postoperative pulmonary infection in patients with non-small cell lung cancer: analysis based on regression models and construction of a nomogram prediction model. Am J Transl Res 2023;15(5):3375-3384, United States 2023. | Timepoint infection not in prediction window |
| 110. | Dong, C.; Yu, Z.; Liu, W.; Liu, H. X.; Tang, Y. K.; Ma, X. J., Establishment and validation of a clinical prediction model for infection risk at the placement sites of skin and soft tissue expanders. Zhonghua Shao Shang Za Zhi Sep 20 2021;37(9):846-852, 2021 Sep 20. | Not in English |
| 111. | Dong Z; Liu G; Tu L; Su X; Yu Y, Establishment of a prediction model of postoperative infection complications in patients with gastric cancer and its impact on prognosis. J Gastrointest Oncol Jun 2023;14(3):1250-1258, China 2023 Jun.  <https://dx.doi.org/10.21037/jgo-23-231> | Timepoint infection not specified |
| 112. | Donkervoort, S. C.; Kortram, K.; Dijksman, L. M.; Boermeester, M. A.; van Ramshorst, B.; Boerma, D., Anticipation of complications after laparoscopic cholecystectomy: prediction of individual outcome, Surg Endosc Dec 2016;30(12):5388-5394, 2016 Dec.  <https://dx.doi.org/10.1007/s00464-016-4895-9> | Wrong outcomes |
| 113. | Du Y; Shi H; Yang X; Wu W, Machine learning for infection risk prediction in postoperative patients with non-mechanical ventilation and intravenous neurotargeted drugs. Front Neurol 2022;13():942023, Switzerland 2022.  <https://dx.doi.org/10.3389/fneur.2022.942023> | Timepoint infection not specified |
| 114. | Egberts JH; Stroeh A; Alkatout I; Goumas FA; Brand PA; Schafmayer C; Becker T; Schniewind B, Preoperative risk evaluation of postoperative morbidity in IBD patients--impact of the POSSUM score. Int J Colorectal Dis Jun 2011;26(6):783-92, Germany 2011 Jun.  <https://dx.doi.org/10.1007/s00384-011-1179-7> | Wrong outcomes |
| 115. | Ejaz A; Schmidt C; Johnston FM; Frank SM; Pawlik TM, Risk factors and prediction model for inpatient surgical site infection after major abdominal surgery. J Surg Res Sep 2017;217():153-159, United States 2017 Sep.  <https://dx.doi.org/10.1016/j.jss.2017.05.018> | Timepoint infection not in prediction window |
| 116. | Ekşi M; Fakir AE; Evren İ; Şam E; Arıkan Y; Kargı T; Hacıislamoğlu A; Yavuzsan AH; Şahin S; Taşçı Aİ, Prediction of infective complications after retrograde intra renal surgery using Machine learning. Minim Invasive Ther Allied Technol Apr 2023;32(2):73-80, England 2023 Apr  <https://dx.doi.org/10.1080/13645706.2023.2186181> | Timepoint infection not specified |
| 117. | El-Daly, I.; Ibraheim, H.; Culpan, P.; Bates, P., Pre-operative Waterlow score: Predicts risk of post-operative infection in patients with neck of femur fractures, Injury 01 Dec 2015;46(12):2394-2398, 2015 01 Dec. | Does not report (internal) validation performance metric(s) |
| 118. | El Hechi, M.; Gebran, A.; Bouardi, H. T.; Maurer, L. R.; El Moheb, M.; Zhuo, D.; Dunn, J.; Bertsimas, D.; Velmahos, G. C.; Kaafarani, H. M. A., Validation of the artificial intelligence-based trauma outcomes predictor (TOP) in patients 65 years and older, Surgery Dec 23 2021;(): 2021 Dec 23.  <https://dx.doi.org/10.1016/j.surg.2021.11.016> | Timepoint infection not specified |
| 119. | El Hechi M; Gebran A; Bouardi HT; Maurer LR; El Moheb M; Zhuo D; Dunn J; Bertsimas D; Velmahos GC; Kaafarani HMA, Validation of the artificial intelligence-based trauma outcomes predictor (TOP) in patients 65 years and older. Surgery Jun 2022;171(6):1687-1694, United States 2022 Jun.  <https://dx.doi.org/10.1016/j.surg.2021.11.016> | Timepoint infection not specified |
| 120. | El'kin, A. V.; Titarenko, O. T.; Esmedliaeva, D. S.; D'Iakova M, E.; Alekseeva, N. P.; Perova, T. L., Assessment of a risk for postoperative infectious complications in patients with fibrocarvous pulmonary tuberculosis, Probl Tuberk Bolezn Legk 2009;(5):31-4, 2009. | Not in English |
| 121. | Elhage, S. A.; Deerenberg, E. B.; Ayuso, S. A.; Murphy, K. J.; Shao, J. M.; Kercher, K. W.; Smart, N. J.; Fischer, J. P.; Augenstein, V. A.; Colavita, P. D.; Heniford, B. T., Development and Validation of Image-Based Deep Learning Models to Predict Surgical Complexity and Complications in Abdominal Wall Reconstruction, JAMA Surg Oct 1 2021;156(10):933-940, 2021 Oct 1.  <https://dx.doi.org/10.1001/jamasurg.2021.3012> | Timepoint infection not specified |
| 122. | Er, S.; Ozden, S.; Koca, F.; Yildiz, B. D.; Yuksel, B. C.; Tez, M., External validation of anastomotic leakage risk analysis system in patients who underwent colorectal resection, Turkish Journal of Medical Sciences 2019;49(1):279-282, 2019.  <https://dx.doi.org/10.3906/sag-1807-205> | Timepoint infection not in prediction window |
| 123. | Ercole FF; Chianca TC; Duarte D; Starling CE; Carneiro M, Surgical site infection in patients submitted to orthopedic surgery: the NNIS risk index and risk prediction. Rev Lat Am Enfermagem Mar-Apr 2011;19(2):269-76, Brazil 2011 Mar-Apr.  <https://dx.doi.org/10.1590/s0104-11692011000200007> | Wrong patient population |
| 124. | Fan, J.; Wan, S.; Liu, L.; Zhao, Z.; Mai, Z.; Chen, D.; Zhu, W.; Yang, Z.; Ou, L.; Wu, W., Predictors for uroseptic shock in patients who undergo minimally invasive percutaneous nephrolithotomy, Urolithiasis Dec 2017;45(6):573-578, 2017 Dec.  <https://dx.doi.org/10.1007/s00240-017-0963-4> | Timepoint infection not specified |
| 125. | Farhat JS; Velanovich V; Falvo AJ; Horst HM; Swartz A; Patton JH Jr; Rubinfeld IS, Are the frail destined to fail? Frailty index as predictor of surgical morbidity and mortality in the elderly. J Trauma Acute Care Surg Jun 2012;72(6):1526-30; discussion 1530-1, United States 2012 Jun.  <https://dx.doi.org/10.1097/TA.0b013e3182542fab> | Wrong outcomes |
| 126. | Farinas-Alvarez, C.; Farinas, M. C.; Prieto, D.; Delgado-Rodriguez, M., Applicability of two surgical-site infection risk indices to risk of sepsis in surgical patients, Infection Control and Hospital Epidemiology 2000;21(10):633‐638, 2000. | Wrong patient population |
| 127. | Farrokhi F; Buchlak QD; Sikora M; Esmaili N; Marsans M; McLeod P; Mark J; Cox E; Bennett C; Carlson J, Investigating Risk Factors and Predicting Complications in Deep Brain Stimulation Surgery with Machine Learning Algorithms. World Neurosurg Feb 2020;134():e325-e338, United States 2020 Feb.  <https://dx.doi.org/10.1016/j.wneu.2019.10.063> | Timepoint infection not in prediction window |
| 128. | Feher B; Lettner S; Heinze G; Karg F; Ulm C; Gruber R; Kuchler U, An advanced prediction model for postoperative complications and early implant failure. Clin Oral Implants Res Oct 2020;31(10):928-935, 2020 Oct.  <https://dx.doi.org/10.1111/clr.13636> | Wrong outcomes |
| 129. | Feld SI; Cobian AG; Tevis SE; Kennedy GD; Craven MW, Modeling the Temporal Evolution of Postoperative Complications. AMIA Annu Symp Proc 2016;2016():551-559, 2016. | Timepoint infection not in prediction window |
| 130. | Fernandez-Moure JS; Wes A; Kaplan LJ; Fischer JP, Actionable Risk Model for the Development of Surgical Site Infection after Emergency Surgery. Surg Infect (Larchmt) Mar 2021;22(2):168-173, United States 2021 Mar.  <https://dx.doi.org/10.1089/sur.2019.282> | Timepoint infection not specified |
| 131. | Fernandez-Ruiz, M.; Seron, D.; Alonso, A.; Lora, D.; Hernandez, D.; Gonzalez, E.; Perez-Saez, M. J.; Gomez, G.; Pallardo-Mateu, L. M.; Jimeno-Garcia, L.; Cofan, F.; Gutierrez-Dalmau, A.; Ruiz, J. C.; Ramirez-Puga, A.; Santana Estupinan, R.; Marcen, R.; Portoles, J. M.; Munoz-Cepeda, M. A.; Lopez-Medrano, F.; San Juan, R.; Andres, A.; Aguado, J. M., Derivation and external validation of the SIMPLICITY score as a simple immune-based risk score to predict infection in kidney transplant recipients, Kidney International October 2020;98(4)():1031-1043, 2020 October.  <https://dx.doi.org/10.1016/j.kint.2020.04.054> | Timepoint infection not specified |
| 132. | Fernández-Ugidos P; Barge-Caballero E; Gómez-López R; Paniagua-Martin MJ; Barge-Caballero G; Couto-Mallón D; Solla-Buceta M; Iglesias-Gil C; Aller-Fernández V; González-Barbeito M; Vázquez-Rodríguez JM; Crespo-Leiro MG, In-hospital postoperative infection after heart transplantation: Risk factors and development of a novel predictive score. Transpl Infect Dis Aug 2019;21(4):e13104, Denmark 2019 Aug.  <https://dx.doi.org/10.1111/tid.13104> | Timepoint infection not in prediction window |
| 133. | Findlay JM; Tilson RC; Harikrishnan A; Sgromo B; Marshall RE; Maynard ND; Gillies RS; Middleton MR, Attempted validation of the NUn score and inflammatory markers as predictors of esophageal anastomotic leak and major complications. Dis Esophagus Oct 2015;28(7):626-33, United States 2015 Oct.  <https://dx.doi.org/10.1111/dote.12244> | Timepoint infection not in prediction window |
| 134. | Fischer, J. P.; Wes, A. M.; Tuggle, C. T.; Serletti, J. M.; Wu, L. C., Risk analysis and stratification of surgical morbidity after immediate breast reconstruction, J Am Coll Surg Nov 2013;217(5):780-7, 2013 Nov.  <https://dx.doi.org/10.1016/j.jamcollsurg.2013.07.004> | Wrong outcomes |
| 135. | Fletcher, R. R.; Schneider, G.; Bikorimana, L.; Rukundo, G.; Niyigena, A.; Miranda, E.; Riviello, R.; Kateera, F.; Hedt-Gauthier, B., The Use of Mobile Thermal Imaging and Deep Learning for Prediction of Surgical Site Infection, Annu Int Conf IEEE Eng Med Biol Soc Nov 2021;2021():5059-5062, 2021 Nov.  <https://dx.doi.org/10.1109/embc46164.2021.9630094> | Does not report (internal) validation performance metric(s) |
| 136. | Fletcher RR; Olubeko O; Sonthalia H; Kateera F; Nkurunziza T; Ashby JL; Riviello R; Hedt-Gauthier B, Application of Machine Learning to Prediction of Surgical Site Infection. Annu Int Conf IEEE Eng Med Bio Soc Jul 2019;2019():2234-2237, United States 2019 Jul.  <https://dx.doi.org/10.1109/EMBC.2019.8857942> | Timepoint infection not in prediction window |
| 137. | Fligor, J.; Lanier, S.; Dumanian, G., Three risk stratification systems are non-predictive of surgical site infections and occurrences in midline ventral hernias, Hernia March 2016;20(1):S56, 2016 March.  <https://dx.doi.org/10.1007/s10029-016-1468-8> | Conference abstract |
| 138. | Formeister EJ; Baum R; Knott PD; Seth R; Ha P; Ryan W; El-Sayed I; George J; Larson A; Plonowska K; Heaton C, Machine Learning for Predicting Complications in Head and Neck Microvascular Free Tissue Transfer. Laryngoscope Dec 2020;130(12):E843-E849, United States 2020 Dec.  <https://dx.doi.org/10.1002/lary.28508> | Timepoint infection not specified |
| 139. | Foster CA; Charles EJ; Turrentine FE; Sohn MW; Kron IL; Jones RS, Development and Validation of Procedure-Specific Risk Score for Predicting Postoperative Pulmonary Complication: A NSQIP Analysis. J Am Coll Surg Oct 2019;229(4):355-365.e3, United States 2019 Oct.  <https://dx.doi.org/10.1016/j.jamcollsurg.2019.05.028> | Wrong outcomes |
| 140. | Franzotti SADS; Sloboda DA; Silva JR; Souza EAS; Reboreda JZ; Ferretti-Rebustini REL; Nogueira LS, Performance of Severity Indices to Estimate Postoperative Complications of Myocardial Revascularization. Arq Bras Cardiol Sep 2020;115(3):452-459, Brazil 2020 Sep.  <https://dx.doi.org/10.36660/abc.20190120> | Timepoint infection not specified |
| 141. | Friedman, N. D.; Bull, A. L.; Russo, P. L.; Gurrin, L.; Richards, M., Performance of the national nosocomial infections surveillance risk index in predicting surgical site infection in Australia, Infect Control Hosp Epidemiol Jan 2007;28(1):55-9, 2007 Jan.  <https://dx.doi.org/10.1086/509848> | Timepoint infection not in prediction window |
| 142. | Fukuda H; Kuroki M, The Development of Statistical Models for Predicting Surgical Site Infections in Japan: Toward a Statistical Model-Based Standardized Infection Ratio. Infect Control Hosp Epidemiol Mar 2016;37(3):260-71, United States 2016 Mar.  <https://dx.doi.org/10.1017/ice.2015.302> | Timepoint infection not specified |
| 143. | Fukuda H; Morikane K; Kuroki M; Taniguchi S; Shinzato T; Sakamoto F; Okada K; Matsukawa H; Ieiri Y; Hayashi K; Kawai S, Toward the rational use of standardized infection ratios to benchmark surgical site infections. Am J Infect Control Sep 2013;41(9):810-4, United States 2013 Sep.  <https://dx.doi.org/10.1016/j.ajic.2012.10.004> | Wrong outcomes |
| 144. | Galandiuk, S.; Britt, R.; Langham, M.; Bergquist, J., Failure of Colorectal Surgical Site Infection Predictive Models Applied to an Independent Dataset: Do They Add Value or Just Confusion? Discussion, Journal of the American College of Surgeons Apr 2016;222(4):438-439, 2016 Apr. | Duplicate (exact similar publication in other journal) |
| 145. | Galivanche, A. R.; Huang, J. K.; Mu, K. W.; Varthi, A. G.; Grauer, J. N., Ensemble Machine Learning Algorithms for Prediction of Complications after Elective Total Hip Arthroplasty, Journal of the American College of Surgeons October 2019;229 (4 Supplement 1)():S194-S195, 2019 October.  <https://dx.doi.org/10.1016/j.jamcollsurg.2019.08.429> | Conference abstract |
| 146. | Gao, Y.; Wang, C.; Wang, Y.; Li, J.; Wang, J.; Wang, S.; Tian, Y.; Liu, J.; Diao, X.; Zhao, W., Establishment and Validation of a Nomogram to Predict Hospital-Acquired Infection in Elderly Patients After Cardiac Surgery, Clin Interv Aging 2022;17():141-150, 2022.  <https://dx.doi.org/10.2147/cia.S351226> | Timepoint infection not specified |
| 147. | Gatti G; Barbati G; Luzzati R; Sinagra G; Pappalardo A, Prospective validation of a predictive scoring system for deep sternal wound infection after routine bilateral internal thoracic artery grafting. Interact Cardiovasc Thorac Surg May 2016;22(5):606-11, 2016 May.  <https://dx.doi.org/10.1093/icvts/ivw016> | Timepoint infection not in prediction window |
| 148. | Gatti G; Fiore A; Ceschia A; Ecarnot F; Chaara R; Luzzati R; Folliguet T; Chocron S; Pappalardo A; Perrotti A, Risk stratification tool for all surgical site infections after coronary artery bypass grafting. Infect Control Hosp Epidemiol Feb 2021;42(2):182-193, United States 2021 Feb.  <https://dx.doi.org/10.1017/ice.2020.412> | Timepoint infection not in prediction window |
| 149. | Gatti, G.; Michelotti, S.; Ceschia, A.; Fiore, A.; Perrotti, A.; Chocron, S.; Folliguet, T.; Pappalardo, A., Risk stratification tool for all surgical site infections after coronaryartery bypass grafting, European Heart Journal, Supplement August 2020;22(SUPPL G)():G100, 2020 August.  <https://dx.doi.org/10.1093/eurheartj/suaa106> | Duplicate (exact similar publication in other journal) |
| 150. | Gatti G; Rochon M; Raja SG; Luzzati R; Dreas L; Pappalardo A, Predictive models of surgical site infections after coronary surgery: insights from a validation study on 7090 consecutive patients. J Hosp Infect Jul 2019;102(3):277-286, England 2019 Jul.  <https://dx.doi.org/10.1016/j.jhin.2019.01.009> | Timepoint infection not in prediction window |
| 151. | Gbegnon, A.; Monestina, J.; Cromwell, J. W., Machine learning algorithm for accurate, automated, real-time prediction of surgical site infections using EHR data, Journal of Surgical Research February 2014;186 (2)():527, 2014 February.  <https://dx.doi.org/10.1016/j.jss.2013.11.380> | Conference abstract |
| 152. | Gelbard RB; Hensman H; Schobel S; Khatri V; Tracy BM; Dente CJ; Buchman T; Kirk A; Elster E, Random forest modeling can predict infectious complications following trauma laparotomy. J Trauma Acute Care Surg Nov 2019;87(5):1125-1132, United States 2019 Nov.  <https://dx.doi.org/10.1097/TA.0000000000002486> | Timepoint infection not specified |
| 153. | Geraghty, R.; Finch, W.; Fowler, S.; Sriprasad, S.; Smith, D.; Dickinson, A.; Gall, Z.; Somani, B., Machine learning can predict the need for post-PCNL transfusion and infectious complications with an extremely high degree of accuracy: Analysis of UK BAUS PCNL database, European Urology February 2022;81(Supplement 1)():S1517-S1518, 2022 February.  <https://dx.doi.org/10.1016/S0302-2838> | Conference abstract |
| 154. | Geraghty, R.; Fitzpatrick, J.; Harding, C.; Veeratterapillay, R.; Rogers, A.; Shaw, M., A machine learning study of postureteroscopy infectious complications, Journal of Clinical Urology June 2021;14(1 SUPPL)():94, 2021 June.  <https://dx.doi.org/10.1177/20514158211013240> | Does not report (internal) validation performance metric(s) |
| 155. | Geubbels, N.; de Brauw, L. M.; Acherman, Y. I. Z.; van de Laar, Awjm; Bruin, S. C., Risk Stratification Models: How Well do They Predict Adverse Outcomes in a Large Dutch Bariatric Cohort? Obesity Surgery Dec 2015;25(12):2290-2301, 2015 Dec.  <https://dx.doi.org/10.1007/s11695-015-1699-2> | Wrong outcomes |
| 156. | Ghani, R.; Hutt, J.; Mitchell, P.; Granger, L.; Sandiford, N. A., Serial C-reactive Protein Monitoring in Prosthetic Joint Infection: A Powerful Predictor or Potentially Pointless? Cureus Feb 12 2020;12(2):e6967, 2020 Feb 12.  <https://dx.doi.org/10.7759/cureus.6967> | Timepoint infection not in prediction window |
| 157. | Gibbons C; Bruce J; Carpenter J; Wilson AP; Wilson J; Pearson A; Lamping DL; Krukowski ZH; Reeves BC, Identification of risk factors by systematic review and development of risk-adjusted models for surgical site infection. Health Technol Assess Sep 2011;15(30):1-156, iii-iv, England 2011 Sep.  <https://dx.doi.org/10.3310/hta15300> | Timepoint infection not specified |
| 158. | Glazyrine, V.; Graw, S.; Niu, S.; Jensen, D.; Koestler, D.; Lee, E., Preoperative risk factors predicting postoperative complications in radical cystectomy for bladder cancer, Journal of Urology April 2017;197 (4 Supplement 1)():e108-e109, 2017 April. | Conference abstract |
| 159. | Godil SS; Shamim MS; Enam SA; Qidwai U; Qadeer M; Sobani ZA, Cranial reconstruction after decompressive craniectomy: prediction of complications using fuzzy logic. J Craniofac Surg Jul 2011;22(4):1307-11, United States 2011 Jul.  <https://dx.doi.org/10.1097/SCS.0b013e31821c6d37> | Timepoint infection not specified |
| 160. | Goel N; Manstein SM; Ward WH; DeMora L; Smaldone MC; Farma JM; Uzzo RG; Esnaola NF, Does the Surgical Apgar Score predict serious complications after elective major cancer surgery? J Surg Res Nov 2018;231():242-247, United States 2018 Nov.  <https://dx.doi.org/10.1016/j.jss.2018.05.037> | Wrong outcomes |
| 161. | González-Martínez S; Olona Tabueña N; Martín Baranera M; Martí-Saurí I; Moll JL; Morales García MÁ; Borrell Grau N; Pueyo Zurdo JM, Inflammatory markers as predictors of postoperative adverse outcome in octogenarian surgical patients: an observational prospective study. Cir Esp Mar 2015;93(3):166-73, Spain 2015 Mar.  <https://dx.doi.org/10.1016/j.ciresp.2014.08.006> | Wrong outcomes |
| 162. | Greenblatt DY; Kelly KJ; Rajamanickam V; Wan Y; Hanson T; Rettammel R; Winslow ER; Cho CS; Weber SM, Preoperative factors predict perioperative morbidity and mortality after pancreaticoduodenectomy. Ann Surg Oncol Aug 2011;18(8):2126-35, United States 2011 Aug.  <https://dx.doi.org/10.1245/s10434-011-1594-6> | Wrong outcomes |
| 163. | Guilbaud T; Birnbaum DJ; Lemoine C; Chirica M; Risse O; Berdah S; Girard E; Moutardier V, C-Reactive Protein on Postoperative Day 1 Is a Reliable Predictor of Pancreas-Specific Complications After Pancreaticoduodenectomy. J Gastrointest Surg May 2018;22(5):818-830, United States 2018 May.  <https://dx.doi.org/10.1007/s11605-017-3658-9> | Wrong outcomes |
| 164. | Gupta M; Dugan A; Chacon E; Davenport DL; Shah MB; Marti F; Roth JS; Bernard A; Zwischenberger JB; Gedaly R, Detailed perioperative risk among patients with extreme obesity undergoing nonbariatric general surgery. Surgery Sep 2020;168(3):462-470. United States 2020 Sep.  <https://dx.doi.org/10.1016/j.surg.2020.03.016> | Wrong study design |
| 165. | Gurunathan U; Rapchuk IL; Dickfos M; Larsen P; Forbes A; Martin C; Leslie K; Myles PS, Association of Obesity With Septic Complications After Major Abdominal Surgery: A Secondary Analysis of the RELIEF Randomized Clinical Trial. JAMA Netw Open Nov 2019;2(11):e1916345, 2019 Nov.  <https://dx.doi.org/10.1001/jamanetworkopen.2019.16345> | Wrong study design |
| 166. | Gwilym, B. L.; Ambler, G. K.; Saratzis, A.; Bosanquet, D. C., Groin Wound Infection after Vascular Exposure (GIVE) Risk Prediction Models: Development, Internal Validation, and Comparison with Existing Risk Prediction Models Identified in a Systematic Literature Review. Eur J Vasc Endovasc Surg Aug 2021;62(2):258-266, 2021 Aug.  <https://dx.doi.org/10.1016/j.ejvs.2021.05.009> | Timepoint infection not specified |
| 167. | Hani, S. M.; Fahmi, M. N.; Dewi, F. S. T., Neutrophil-lymphocyte ratio as an independent preoperative risk factor of surgical site infection in ovarian cancer patients undergoing primary surgery, Journal of Obstetrics and Gynaecology Research August 2020;46 (SUPPL 1)():131, 2020 August.  <https://dx.doi.org/10.1111/jog.14462> | Conference abstract |
| 168. | Hanwright PJ; Davila AA; Mioton LM; Fine NA; Bilimoria KY; Kim JY, A predictive model of risk and outcomes in tissue expander reconstruction: a multivariate analysis of 9786 patients. J Plast Surg Hand Surg Dec 2013;47(6):513-8, Sweden 2013 Dec.  <https://dx.doi.org/10.3109/2000656X.2013.789436> | Wrong study design |
| 169. | Hao X; Wang X; Wei H; Ding H; Zheng S; Wang L; Li Z; Yin H, Development and Validation of the Prediction Model of Sepsis in Patients After Percutaneous Nephrolithotomy and Sepsis Progresses to Septic Shock. J Endourol Apr 2023;37(4):377-386, United States 2023 Apr.  <https://dx.doi.org/10.1089/end.2022.0384> | Timepoint infection not specified |
| 170. | Haskins IN; Olson MA; Stewart TG; Rosen MJ; Poulose BK, Development and Validation of the Ventral Hernia Repair Outcomes Reporting App for Clinician and Patient Engagement (ORACLE). J Am Coll Surg Sep 2019;229(3):259-266, United States 2019 Sep.  <https://dx.doi.org/10.1016/j.jamcollsurg.2019.03.014> | Timepoint infection not in prediction window |
| 171. | Hedrick, T.; Friel, C.; Sawyer, R.; Stukenborg, G., A method for estimating the risk of surgical site infection in colorectal surgery, Diseases of the Colon and Rectum May 2012;55 (5)():e272, 2012 May. | Conference abstract |
| 172. | Heredia-Rodríguez M; Bustamante-Munguira J; Lorenzo M; Gómez-Sánchez E; Álvarez FJ; Fierro I; Conejo E; Tamayo E, Procalcitonin and white blood cells, combined predictors of infection in cardiac surgery patients. J Surg Res May 2017;212():187-194, United States 2017 May.  <https://dx.doi.org/10.1016/j.jss.2017.01.021> | Wrong study design |
| 173. | Hill SS; Harnsberger CR; Crawford AS; Hoang CM; Davids JS; Sturrock PR; Maykel JA; Alavi K, Creation and Institutional Validation of a Readmission Risk Calculator for Elective Colorectal Surgery. Dis Colon Rectum Oct 2020;63(10):1436-1445, United States 2020 Oct.  <https://dx.doi.org/10.1097/DCR.0000000000001674> | Wrong outcomes |
| 174. | Hirai, T.; Kobayashi, H.; Okuma, T.; Ishibashi, Y.; Ikegami, M.; Ohki, T.; Shinoda, Y.; Okajima, K.; Zhang, L.; Akiyama, T.; Goto, T.; Tanaka, S., Skeletal muscle measurements predict surgical wound complications but not overall survival in patients with soft tissue sarcoma, Jpn J Clin Oncol Sep 28 2020;50(10):1168-1174, 2020 Sep 28.  <https://dx.doi.org/10.1093/jjco/hyaa100> | Wrong outcomes |
| 175. | Hirose H; Inaba H; Noguchi C; Tambara K; Yamamoto T; Yamasaki M; Kikuchi K; Amano A, EuroSCORE predicts postoperative mortality, certain morbidities, and recovery time. Interact Cardiovasc Thorac Surg Oct 2009;9(4):613-7, England 2009 Oct.  <https://dx.doi.org/10.1510/icvts.2009.210526> | Timepoint infection not in prediction window |
| 176. | Hodari A; Tsiouris A; Eichenhorn M; Horst M; Rubinfeld I, Exploring National Surgical Quality Improvement Program respiratory comorbidities: developing a predictive understanding of postoperative respiratory occurrences, Clavien 4 complications, and death. J Surg Res Aug 2013;183(2):663-7, United States 2013 Aug.  <https://dx.doi.org/10.1016/j.jss.2013.01.031> | Does not report (internal) validation performance metric(s) |
| 177. | Hoeboer SH; Groeneveld AB; Engels N; van Genderen M; Wijnhoven BP; van Bommel J, Rising C-reactive protein and procalcitonin levels precede early complications after esophagectomy. J Gastrointest Surg Apr 2015;19(4):613-24, United States 2015 Apr.  <https://dx.doi.org/10.1007/s11605-015-2745-z> | Timepoint infection not in prediction window |
| 178. | Hoek VT; Buettner S; Sparreboom CL; Detering R; Menon AG; Kleinrensink GJ; Wouters MWJM; Lange JF; Wiggers JK, A preoperative prediction model for anastomotic leakage after rectal cancer resection based on 13.175 patients. Eur J Surg Oncol Dec 2022;48(12):2495-2501, England 2022 Dec.  <https://dx.doi.org/10.1016/j.ejso.2022.06.016> | Wrong outcomes |
| 179. | Hokari S; Ohshima Y; Nakayama H; Suzuki R; Kajiwara T; Koya T; Kagamu H; Takada T; Suzuki E; Narita I, Superiority of respiratory failure risk index in prediction of postoperative pulmonary complications after digestive surgery in Japanese patients. Respir Investig May 2015;53(3):104-10, Netherlands 2015 May.  <https://dx.doi.org/10.1016/j.resinv.2014.12.004> | Wrong outcomes |
| 180. | Hopkins BS; Mazmudar A; Driscoll C; Svet M; Goergen J; Kelsten M; Shlobin NA; Kesavabhotla K; Smith ZA; Dahdaleh NS, Using artificial intelligence (AI) to predict postoperative surgical site infection: A retrospective cohort of 4046 posterior spinal fusions. Clin Neurol Neurosurg May 2020;192():105718, Netherlands 2020 May.  <https://dx.doi.org/10.1016/j.clineuro.2020.105718> | Timepoint infection not specified |
| 181. | Hoshino N; Hida K; Sakai Y; Osada S; Idani H; Sato T; Takii Y; Bando H; Shiomi A; Saito N, Nomogram for predicting anastomotic leakage after low anterior resection for rectal cancer. Int J Colorectal Dis Apr 2018;33(4):411-418, Germany 2018 Apr. | Timepoint infection not specified |
| 182. | Hu B; Tan HY; Rao XW; Jiang JY; Yang K, A Scoring System for Surgical Site Infection after Pancreaticoduodenectomy Using Clinical Data. Surg Infect (Larchmt) Mar 2021;22(2):240-244, United States 2021 Mar.  <https://dx.doi.org/10.1089/sur.2020.082> | Timepoint infection not in prediction window |
| 183. | Hu X; Cheng Y, A Clinical Parameters-Based Model Predicts Anastomotic Leakage After a Laparoscopic Total Mesorectal Excision: A Large Study With Data From China. Medicine (Baltimore) Jul 2015;94(26):e1003, 2015 Jul.  <https://dx.doi.org/10.1097/MD.0000000000001003> | Does not report (internal) validation performance metric(s) |
| 184. | Huang C; Yao H; Huang Q; Lu H; Xu M; Wu J, A novel nomogram to predict the risk of anastomotic leakage in patients after oesophagectomy., BMC Surg Apr 2020;20(1):64, 2020 Apr.  <https://dx.doi.org/10.1186/s12893-020-00726-7> | Timepoint infection not specified |
| 185. | Huang TS; Hu FC; Fan CW; Lee CH; Jwo SC; Chen HY, A simple novel model to predict hospital mortality, surgical site infection, and pneumonia in elderly patients undergoing operation. Dig Surg Aug 2010;27(3):224-31, Switzerland 2010 Aug.  <https://dx.doi.org/10.1159/000274485> | Does not report (internal) validation performance metric(s) |
| 186. | Huang X; Guo Y; Fu R; Li H, A nomogram to predict postoperative surgical site infection of adult patients who received orthopaedic surgery: a retrospective study. Sci Rep May 2023;13(1):8129, England 2023 May.  <https://dx.doi.org/10.1038/s41598-023-34926-x> | Timepoint infection not specified |
| 187. | Huynh, M. J.; Wang, Y.; Joshi, M.; Krasnow, R.; Yu, A. X.; Mossanen, M.; Chung, B. I.; Chang, S. L., Patient factors predict complications after partial nephrectomy: validation and calibration of the Preoperative Risk Evaluation for Partial Nephrectomy (PREP) score, BJU Int Mar 2021;127(3):369-374, 2021 Mar.  <https://dx.doi.org/10.1111/bju.15240> | Wrong outcomes |
| 188. | Hwang JS; Kim SJ; Bamne AB; Na YG; Kim TK, Do glycemic markers predict occurrence of complications after total knee arthroplasty in patients with diabetes? Clin Orthop Relat Res May 2015;473(5):1726-31, United States 2015 May.  <https://dx.doi.org/10.1007/s11999-014-4056-1> | Does not report (internal) validation performance metric(s) |
| 189. | Inacio MC; Pratt NL; Roughead EE; Graves SE, Predicting Infections After Total Joint Arthroplasty Using a Prescription Based Comorbidity Measure. J Arthroplasty Oct 2015;30(10):1692-8, United States 2015 Oct.  <https://dx.doi.org/10.1016/j.arth.2015.05.004> | Timepoint infection not in prediction window |
| 190. | Inose H; Kobayashi Y; Yuasa M; Hirai T; Yoshii T; Okawa A, Procalcitonin and Neutrophil Lymphocyte Ratio After Spinal Instrumentation Surgery. Spine (Phila Pa 1976) Dec 2019;44(23):E1356-E1361, United States 2019 Dec.  <https://dx.doi.org/10.1097/BRS.0000000000003157> | Timepoint infection not specified |
| 191. | Isbell KD; Hatton GE; Wei S; Green C; Truong VTT; Woloski J; Pedroza C; Wade CE; Harvin JA; Kao LS, Risk Stratification for Superficial Surgical Site Infection after Emergency Trauma Laparotomy. Surg Infect (Larchmt) Jan 2021;(): United States 2021 Jan.  <https://dx.doi.org/10.1089/sur.2020.242> | Wrong patient population |
| 192. | Janssen DMC; van Kuijk SMJ; d'Aumerie B; Willems P, A prediction model of surgical site infection after instrumented thoracolumbar spine surgery in adults. Eur Spine J Apr 2019;28(4):775-782, Germany 2019 Apr. | Timepoint infection not in prediction window |
| 193. | Janssen DMC; van Kuijk SMJ; d'Aumerie BB; Willems PC, External validation of a prediction model for surgical site infection after thoracolumbar spine surgery in a Western European cohort. J Orthop Surg Res May 2018;13(1):114, 2018 May.  <https://dx.doi.org/10.1186/s13018-018-0821-2> | Timepoint infection not in prediction window |
| 194. | Jia R; Zhou M; Tuttle CSL; Maier AB, Immune capacity determines outcome following surgery or trauma: a systematic review and meta-analysis., Eur J Trauma Emerg Surg Oct 2020;46(5):979-991, 2020 Oct.  <https://dx.doi.org/10.1007/s00068-019-01271-6> | Wrong outcomes |
| 195. | Jiang E; Guo H; Yang B; Li P; Mishra P; Yang T; Li Y; Wang H; Jiang Y, Predicting and comparing postoperative infections in different stratification following PCNL based on nomograms. Sci Rep Jul 2020;10(1):11337, 2020 Jul.  <https://dx.doi.org/10.1038/s41598-020-68430-3> | Wrong outcomes |
| 196. | Jiang HY; Kohtakangas EL; Asai K; Shum JB, Predictive Power of the NSQIP Risk Calculator for Early Post-Operative Outcomes After Whipple: Experience from a Regional Center in Northern Ontario. J Gastrointest Cancer Sep 2018;49(3):288-294, United States 2018 Sep.  <https://dx.doi.org/10.1007/s12029-017-9949-2> | Does not report (internal) validation performance metric(s) |
| 197. | Jiang, W.; Feng, M. Y.; Dong, X. Y.; Dong, S. M.; Zheng, J. X.; Liu, X. M.; Liu, W. J.; Yan, J., Risk factor analysis on anastomotic leakage after laparoscopic surgery in rectal cancer patient with neoadjuvant therapy and establishment of a nomogram prediction model. Zhonghua Wei Chang Wai Ke Za Zhi Aug 25 2019;22(8):748-754, 2019 Aug 25.  <https://dx.doi.org/10.3760/cma.j.issn.1671-0274.2019.08.009> | Not in English |
| 198. | Jiang, W.; Feng, M.; Zheng, J.; Wang, G.; Xu, S.; Zhou, L.; Zhuo, S.; Yan, J., Association of the collagen score with anastomotic leakage in rectal cancer patients after neoadjuvant chemoradiotherapy, Surgery Nov 2021;170(5):1331-1341, 2021 Nov.  <https://dx.doi.org/10.1016/j.surg.2021.05.023> | Timepoint infection not in prediction window |
| 199. | Jin D; Yuan L; Li F; Wang S; Mao Y, A novel nomogram predicting the risk of postoperative pneumonia for esophageal cancer patients after minimally invasive esophagectomy. Surg Endosc Nov 2022;36(11):8144-8153, Germany 2022 Nov.  <https://dx.doi.org/10.1007/s00464-022-09249-z> | Timepoint infection not specified |
| 200. | Jin F; Liu W; Qiao X; Shi J; Xin R; Jia HQ, Nomogram prediction model of postoperative pneumonia in patients with lung cancer: A retrospective cohort study. Front Oncol 2023;13():1114302, Switzerland 2023. | Timepoint infection not specified |
| 201. | Johnson AJ; Zywiel MG; Stroh A; Marker DR; Mont MA, Serological markers can lead to false negative diagnoses of periprosthetic infections following total knee arthroplasty. Int Orthop Nov 2011;35(11):1621-6, Germany 2011 Nov.  <https://dx.doi.org/10.1007/s00264-010-1175-5> | Timepoint infection not in prediction window |
| 202. | Johnson, C.; Campwala, I.; Gupta, S., Lack of input specificity, great outcome variability, and imprecise risk calculations: Pitfalls of the ACS-NSQIP risk calculator in plastic surgery, Journal of Investigative Medicine January 2016;64 (1)():260, 2016 January.  <https://dx.doi.org/10.1136/jim-d-15-00013.289> | Article not available |
| 203. | Johnson C; Campwala I; Gupta S, Examining the validity of the ACS-NSQIP Risk Calculator in plastic surgery: lack of input specificity, outcome variability and imprecise risk calculations. J Investig Med Mar 2017;65(3):722-725, England 2017 Mar.  <https://dx.doi.org/10.1136/jim-2016-000224> | Does not report (internal) validation performance metric(s) |
| 204. | Johnson RL; Habermann EB; Johnson MQ; Abdel MP; Chamberlain AM; Mantilla CB, How Is Surgical Risk Best Assessed? A Cohort Comparison of Measures in Total Joint Arthroplasty. J Arthroplasty Mar 2021;36(3):851-856.e3, United States 2021 Mar.  <https://dx.doi.org/10.1016/j.arth.2020.09.046> | Timepoint infection not in prediction window |
| 205. | Jonczyk, M. M.; Fisher, C. S.; Babbitt, R.; Paulus, J. K.; Freund, K. M.; Czerniecki, B. J.; Margenthaler, J. A.; Losken, A.; Chatterjee, A. Surgical Predictive Model for Breast Cancer Patients Assessing Acute Postoperative Complications: The Breast Cancer Surgery Risk Calculator (BCSRc), Journal of the American College of Surgeons October 2020;231 (4 Supplement 1)():S43, 2020 October.  <https://dx.doi.org/10.1016/j.jamcollsurg.2020.07.026> | Conference abstract |
| 206. | Junchen Z; Houjing Z; Yun F, Cox Regression Model Analysis of Infection in Renal Transplants After Operation. Transplant Proc Oct 2016;48(8):2678-2683, United States 2016 Oct.  <https://dx.doi.org/10.1016/j.transproceed.2016.08.014> | Timepoint infection not in prediction window |
| 207. | Kamaleswaran, R.; Koo, C.; Helmick, R.; Mas, V.; Eason, J.; Maluf, D., Predicting early post-operative sepsis in liver transplantation applying artificial intelligence, Transplantation August 2019;103 (8 Supplement 1)():42, 2019 August.  <https://dx.doi.org/10.1097/01.tp.0000580472.17422.db> | Conference abstract |
| 208. | Kamaleswaran, R.; Sataphaty, S. K.; Mas, V. R.; Eason, J. D.; Maluf, D. G., Artificial Intelligence May Predict Early Sepsis After Liver Transplantation, Front Physiol 2021;12():692667, 2021.  <https://dx.doi.org/10.3389/fphys.2021.692667> | Timepoint infection not specified |
| 209. | Kanters AE; Krpata DM; Blatnik JA; Novitsky YM; Rosen MJ, Modified hernia grading scale to stratify surgical site occurrence after open ventral hernia repairs. J Am Coll Surg Dec 2012;215(6):787-93, United States 2012 Dec.  <https://dx.doi.org/10.1016/j.jamcollsurg.2012.08.012> | Wrong outcomes |
| 210. | Karamanos E; Kandagatla P; Watson J; Schmoekel N; Siddiqui A, Development and Validation of a Scoring System to Predict Surgical Site Infection After Ventral Hernia Repair: A Michigan Surgical Quality Collaborative Study. World J Surg Apr 2017;41(4):914-918, United States 2017 Apr.  <https://dx.doi.org/10.1007/s00268-016-3835-0> | Timepoint infection not in prediction window |
| 211. | Karliczek, A.; Harlaar, N. J.; Zeebregts, C. J.; Wiggers, T.; Baas, P. C.; van Dam, G. M., Surgeons lack predictive accuracy for anastomotic leakage in gastrointestinal surgery, International Journal of Colorectal Disease 2009;24(5):569-576, 2009.  <https://dx.doi.org/10.1007/s00384-009-0658-6> | Wrong intervention |
| 212. | Kassicieh CS; Kassicieh AJ; Rumalla K; Courville EN; Cole KL; Kazim SF; Bowers CA; Schmidt MH, Hospital-acquired infection following spinal tumor surgery: A frailty-driven pre-operative risk model. Clin Neurol Neurosurg Feb 2023;225():107591, Netherlands 2023 Feb.  <https://dx.doi.org/10.1016/j.clineuro.2023.107591> | Does not report (internal) validation performance metric(s) |
| 213. | Kaufmann, K. B.; Heinrich, S.; Staehle, H. F.; Bogatyreva, L.; Buerkle, H.; Goebel, U., Perioperative cytokine profile during lung surgery predicts patients at risk for postoperative complications-A prospective, clinical study, PLoS One 2018;13(7): 2018.  <https://dx.doi.org/10.1371/journal.pone.0199807> | Wrong outcomes |
| 214. | Kawaguchi Y; Hanaoka J; Ohshio Y; Igarashi T; Kataoka Y; Okamoto K; Kaku R; Hayashi K, A risk score to predict postoperative complications after lobectomy in elderly lung cancer patients. Gen Thorac Cardiovasc Surg Sep 2018;66(9):537-542, Japan 2018 Sep.  <https://dx.doi.org/10.1007/s11748-018-0960-8> | Wrong outcomes |
| 215. | Kawasaki K; Yamamoto M; Suka Y; Kawasaki Y; Ito K; Koike D; Furuya T; Nagai M; Nomura Y; Tanaka N; Kawaguchi Y, Development and validation of a nomogram predicting postoperative pneumonia after major abdominal surgery. Surg Today Sep 2019;49(9):769-777, Japan 2019 Sep.  <https://dx.doi.org/10.1007/s00595-019-01796-8> | Timepoint infection not specified |
| 216. | Kelly KJ; Greenblatt DY; Wan Y; Rettammel RJ; Winslow E; Cho CS; Weber SM, Risk stratification for distal pancreatectomy utilizing ACS-NSQIP: preoperative factors predict morbidity and mortality.  J Gastrointest Surg Feb 2011;15(2):250-9, discussion 259-61, United States 2011 Feb.  <https://dx.doi.org/10.1007/s11605-010-1390-9> | Wrong outcomes |
| 217. | Khanna AK; Kelava M; Ahuja S; Makarova N; Liang C; Tanner D; Insler SR, A nomogram to predict postoperative pulmonary complications after cardiothoracic surgery. J Thorac Cardiovasc Surg Jun 2023;165(6):2134-2146, United States 2023 Jun.  <https://dx.doi.org/10.1016/j.jtcvs.2021.08.034> | Wrong outcomes |
| 218. | Kilic, A.; Ohkuma, R.; Grimm, J. C.; Magruder, J. T.; Sussman, M.; Schneider, E. B.; Whitman, G. J. R., A novel score to estimate the risk of pneumonia after cardiac surgery, Journal of Thoracic and Cardiovascular Surgery 01 May 2016;151(5):1415-1421, 2016 01 May.  <https://dx.doi.org/10.1016/j.jtcvs.2015.12.049> | Timepoint infection not in prediction window |
| 219. | Kim DH; Shim JK; Hong SW; Cho KR; Kang SY; Kwak YL, Predictive value of C-reactive protein for major postoperative complications following off-pump coronary artery bypass surgery: prospective and observational trial. Circ J May 2009;73(5):872-7, Japan 2009 May.  <https://dx.doi.org/10.1253/circj.cj-08-1010> | Does not report (internal) validation performance metric(s) |
| 220. | Kim, H.; Choi, G.; Park, J.; Park, S., Development and validattion of a prediction model for anastomotic leakage risk during laparoscopic low anterior resection: A decision-making tool for the choice of protecive stoma, Diseases of the Colon and Rectum May 2018;61 (5)():e261, 2018 May.  <https://dx.doi.org/10.1097/DCR.0000000000001104> | Conference abstract |
| 221. | Kim, J. S.; Arvind, V.; Oermann, E. K.; Kaji, D.; Ranson, W.; Ukogu, C.; Hussain, A. K.; Caridi, J.; Cho, S. K., Predicting Surgical Complications in Patients Undergoing Elective Adult Spinal Deformity Procedures Using Machine Learning, Spine Deform Nov-Dec 2018;6(6):762-770, 2018 Nov-Dec.  <https://dx.doi.org/10.1016/j.jspd.2018.03.003> | Wrong outcomes |
| 222. | Kim JY; Park IS; Kang DH; Lee YS; Kim KT; Hong SJ, Prediction of Risk Factors after Spine Surgery in Patients Aged >75 Years Using the Modified Frailty Index. J Korean Neurosurg Soc Nov 2020;63(6):827-833, 2020 Nov.  <https://dx.doi.org/10.3340/jkns.2020.0019> | Wrong outcomes |
| 223. | Kisana, H.; Hui, C.; Martin, J. R.; Stecher, C.; Hustedt, J., Development of A Risk Stratification Scoring System to Predict General Surgical Complications in Foot and Ankle Surgery Patients, Foot Ankle Orthop Jan 2022;7(1):2473011421s00282, 2022 Jan.  <https://dx.doi.org/10.1177/2473011421s00282> | Conference abstract |
| 224. | Klein HJ; Csordas A; Falk V; Slankamenac K; Rudiger A; Schönrath F; Rodriguez Cetina Biefer H; Starck CT; Graf R, Pancreatic stone protein predicts postoperative infection in cardiac surgery patients irrespective of cardiopulmonary bypass or surgical technique. PLoS One 2015;10(3):e0120276, United States 2015.  <https://dx.doi.org/10.1371/journal.pone.0120276> | Wrong outcomes |
| 225. | Klose J; Tarantino I; von Fournier A; Stowitzki MJ; Kulu Y; Bruckner T; Volz C; Schmidt T; Schneider M; Büchler MW; Ulrich A, A Nomogram to Predict Anastomotic Leakage in Open Rectal Surgery-Hope or Hype? J Gastrointest Surg Sep 2018;22(9):1619-1630, United States 2018 Sep.  <https://dx.doi.org/10.1007/s11605-018-3782-1> | Timepoint infection not specified |
| 226. | Klose, J.; Tarantino, I.; Von Fournier, A.; Strowitzki, M.; Kulu, Y.; Bruckner, T.; Schmidt, T.; Schneider, M.; Buchler, M. W.; Ulrich, A. B., Do risk scores suit to predict anastomotic leakage in colorectal surgery? validation of a normogram with 972 rectal cancer patients, European Surgical Research April 2018;59 (Supplement 1)():10, 2018 April.  <https://dx.doi.org/10.1159/000488177> | Conference abstract |
| 227. | Koakutsu T; Sato T; Aizawa T; Itoi E; Kushimoto S, Postoperative Changes in Presepsin Level and Values Predictive of Surgical Site Infection After Spinal Surgery: A Single-Center, Prospective Observational Study. Spine (Phila Pa 1976) Apr 2018;43(8):578-584, United States 2018 Apr  <https://dx.doi.org/10.1097/BRS.0000000000002376> | Does not report (internal) validation performance metric(s) |
| 228. | Kocbek P; Fijacko N; Soguero-Ruiz C; Mikalsen KØ; Maver U; Povalej Brzan P; Stozer A; Jenssen R; Skrøvseth SO; Stiglic G, Maximizing Interpretability and Cost-Effectiveness of Surgical Site Infection (SSI) Predictive Models Using Feature-Specific Regularized Logistic Regression on Preoperative Temporal Data. Comput Math Methods Med 2019;2019():2059851, 2019.  <https://dx.doi.org/10.1155/2019/2059851> | Timepoint infection not specified |
| 229. | Kohut AY; Liu JJ; Stein DE; Sensenig R; Poggio JL, Patient-specific risk factors are predictive for postoperative adverse events in colorectal surgery: an American College of Surgeons National Surgical Quality Improvement Program-based analysis. Am J Surg Feb 2015;209(2):219-29, United States 2015 Feb.  <https://dx.doi.org/10.1016/j.amjsurg.2014.08.020> | Timepoint infection not in prediction window |
| 230. | Kong X; Liu K, The Predictive Value of PCT and Other Infection Indicators in Postoperative Infection of Epithelial Ovarian Cancer. Infect Drug Resist 2023;16():1521-1536, New Zealand 2023.  <https://dx.doi.org/10.2147/IDR.S399666> | Timepoint infection not specified |
| 231. | Kosaka, H.; Asano, Y.; Suzumura, K.; Sueoka, H.; Uyama, N.; Okada, T.; Hirano, T.; Iimuro, Y.; Fujimoto, J., The validation analysis of our prediction method for postoperative pancreatic fistula after pancreas head resection, Pancreas November 2014;43 (8)():1382, 2014 November.  <https://dx.doi.org/10.1097/MPA.0000000000000231> | Conference abstract |
| 232. | Koshizaka M; Ishibashi R; Maeda Y; Ishikawa T; Maezawa Y; Takemoto M; Yokote K, Predictive model and risk engine web application for surgical site infection risk in perioperative patients with type 2 diabetes. Diabetol Int Oct 2022;13(4):657-664,Japan 2022 Oct  <https://dx.doi.org/10.1007/s13340-022-00587-w> | Wrong patient population |
| 233. | Koshizaka, M.; Ishibashi, R.; Maeda, Y.; Ishikawa, T.; Maezawa, Y.; Takemoto, M.; Yokote, K., Prediction method for infection risk in perioperative patients with diabetes, Diabetes. Conference: 79th Scientific Sessions of the American Diabetes Association, ADA 2019;68(Supplement 1): 2019.  <https://dx.doi.org/10.2337/db19-1592-P> | Conference abstract |
| 234. | Kostić Z; Panišić M; Milev B; Mijušković Z; Slavković D; Ignjatović M, Diagnostic value of serial measurement of C-reactive protein in serum and matrix metalloproteinase-9 in drainage fluid in the detection of infectious complications and anastomotic leakage in patients with colorectal resection. Vojnosanit Pregl Oct 2015;72(10):889-98, Serbia 2015 Oct.  <https://dx.doi.org/10.2298/vsp140723011k> | Wrong study design |
| 235. | Kunisaki C; Miyata H; Konno H; Saze Z; Hirahara N; Kikuchi H; Wakabayashi G; Gotoh M; Mori M, Modeling preoperative risk factors for potentially lethal morbidities using a nationwide Japanese web-based database of patients undergoing distal gastrectomy for gastric cancer. Gastric Cancer May 2017;20(3):496-507, Japan 2017 May.  <https://dx.doi.org/10.1007/s10120-016-0634-0> | Timepoint infection not specified |
| 236. | Kunutsor SK; Whitehouse MR; Blom AW; Beswick AD, Systematic review of risk prediction scores for surgical site infection or periprosthetic joint infection following joint arthroplasty. Epidemiol Infect Jul 2017;145(9):1738-1749, England 2017 Jul.  <https://dx.doi.org/10.1017/S0950268817000486> | Wrong study design |
| 237. | Kuo, F. C.; Hu, W. H.; Hu, Y. J., Periprosthetic Joint Infection Prediction via Machine Learning: Comprehensible Personalized Decision Support for Diagnosis, J Arthroplasty Jan 2022;37(1):132-141, 2022 Jan.  <https://dx.doi.org/10.1016/j.arth.2021.09.005> | Timepoint infection not in prediction window |
| 238. | Kuo PJ; Wu SC; Chien PC; Chang SS; Rau CS; Tai HL; Peng SH; Lin YC; Chen YC; Hsieh HY; Hsieh CH, Artificial neural network approach to predict surgical site infection after free-flap reconstruction in patients receiving surgery for head and neck cancer. Oncotarget Mar 2018;9(17):13768-13782, 2018 Mar.  <https://dx.doi.org/10.18632/oncotarget.24468> | Timepoint infection not specified |
| 239. | Lago, V.; Fotopoulou, C.; Chiantera, V.; Minig, L.; Gil-Moreno, A.; Cascales-Campos, P. A.; Jurado, M.; Tejerizo, A.; Padilla-Iserte, P.; Malune, M. E.; Di Dona, M. C.; Marina, T.; Sanchez-Iglesias, J. L.; Olloqui, A.; Garcia-Granero, A.; Matute, L.; Fornes, V.; Domingo, S. Risk factors for anastomotic leakage after colorectal resection in ovarian cancer surgery: A multi-centre study, International Journal of Gynecological Cancer November 2019;29 (Supplement 4)():A140, 2019 November.  <https://dx.doi.org/10.1136/ijgc-2019-ESGO.198> | Timepoint infection not specified |
| 240. | Lago V; Segarra-Vidal B; Cappucio S; Angeles MA; Fotopoulou C; Muallem MZ; Manzanedo I; Iglesias JLS; Chacón E; Padilla-Iserte P; Fagotti A; Ferron G; Kluge L; Vargiu V; Del M; Scambia G; Minig L; Tejerizo Á; Segovia MG; Cascales-Campos PA; Hervás D; Domingo S, OVA-LEAK: Prognostic score for colo-rectal anastomotic leakage in patients undergoing ovarian cancer surgery. Gynecol Oncol Oct 2022;167(1):22-27, United States 2022 Oct.  <https://dx.doi.org/10.1016/j.ygyno.2022.08.004> | Timepoint infection not specified |
| 241. | Lago, V.; Segarra Vidal, B.; Padilla Iserte, P.; Gil-Moreno, A.; Cascales, P.; Luis Javier, M. T.; S. Anchez Iglesias J.L; Gurrea, M.; Domingo, S., Ova-leak: Prognostic score for colo-rectal anastomotic leakage in patients undergoing ovariancancer surgery, International Journal of Gynecological Cancer October 2021;31(SUPPL 1)():A374, 2021 October.  <https://dx.doi.org/10.1136/ijgc-2021-ESGO.663> | Conference abstract |
| 242. | Laih, C. Y.; Hsiao, P. J.; Huang, C. P.; Chen, W. C.; Chou, C. L.; Chang, C. H., China Medical University Hospital-post ureteral lithotripsy sepsis score (CMUH-PULSE score), is a brand new tool to precisely predict postoperative sepsis rate resulting from ureteroscopic lithotripsy (URSL), International Journal of Urology August 2019;26 (Supplement 2)():78-79, 2019 August.  <https://dx.doi.org/10.1111/iju.14061> | Conference abstract |
| 243. | Laih, C. Y.; Lai, C. M.; Hsiao, P. J.; Huang, C. P.; Chen, W. C.; Chou, C. L.; Chang, C. H., Post Ureteral Lithotripsy Sepsis Evaluation Score (CMUH-PULSE score)is a brand new tool to precise predict postoperative sepsis rate resulting from ureteroscopic lithotripsy (URSL), European Urology, Supplements March 2019;18 (1)():e2015-e2016, 2019 March  <https://dx.doi.org/10.1016/S1569-9056> | Conference abstract |
| 244. | Lan, N.; Shen, B., Predication of Postoperative Outcome of Colectomy/Proctocolectomy in Ulcerative Colitis Patients using Model of End-Stage Liver Disease Score, Gastroenterology May 2018;154 (6 Supplement 1)():S-355-S-356, 2018 May  <https://dx.doi.org/10.1016/S0016-5085> | Wrong outcomes |
| 245. | Lapshyn, H.; Billmann, F.; Makowiec, F.; Bausch, D.; Hopt, U.; Keck, T.; Wellner, U., Preoperative prediction of the postoperative pancreatic f istula and other severe complications in pancreatoduodenectomy by artificial neuronal network analysis, Langenbeck's Archives of Surgery March 2014;399 (3)():397, 2014 March.  <https://dx.doi.org/10.1007/s00423-014-1175-9> | Conference abstract |
| 246. | Lapshyn, H.; Makowiec, F.; Bausch, D.; Hopt, U. T.; Keck, T.; Wellner, U., Preoperative prediction of the "high-risk pancreas" by artificial neuronal network analysis of over 450 pancreatoduodenectomies, Gastroenterology May 2013;144(5):S1082, 2013 May. | Conference abstract |
| 247. | Lara, C.; Cárcamo, M.; Cerón, I.; Adasme, R.; Urquidi, C.; Cavada, G., Predictive model preparation for surgical wound infection in adult patients undergoing total hip arthroplasty in high complexity hospital for years 2012 and 2014, Rev Chilena Infectol Jun 2019;36(3):265-273, 2019 Jun.  <https://dx.doi.org/10.4067/s0716-10182019000300265> | Not in English |
| 248. | Lascano, D.; Pak, J. S.; Lipsky, M. J.; Finkelstein, J. B.; Benson, M. C.; DeCastro, G. J.; McKiernan, J. M., Simplified frailty index predicts adverse outcomes in radical cystectomy: An analysis of the ACS-NSQIP database, Journal of Urology April 2015;193(4):e800, 2015 April. | Conference abstract |
| 249. | Lawrence, V. A. Predicting postoperative pulmonary complications: the sleeping giant stirs, Ann Intern Med Nov 20 2001;135(10):919-21, 2001 Nov 20  <https://dx.doi.org/10.7326/0003-4819-135-10-200111200-00012> | Wrong study design |
| 250. | Leclère B; Lasserre C; Bourigault C; Juvin ME; Chaillet MP; Mauduit N; Caillon J; Hanf M; Lepelletier D, Matching bacteriological and medico-administrative databases is efficient for a computer-enhanced surveillance of surgical site infections: retrospective analysis of 4,400 surgical procedures in a French university hospital. Infect Control Hosp Epidemiol Nov 2014;35(11):1330-5, United States 2014 Nov  <https://dx.doi.org/10.1086/678422> | Wrong outcomes |
| 251. | Lee JS; Terjimanian MN; Tishberg LM; Alawieh AZ; Harbaugh CM; Sheetz KH; Holcombe SA; Wang SC; Sonnenday CJ; Englesbe MJ, Surgical site infection and analytic morphometric assessment of body composition in patients undergoing midline laparotomy. J Am Coll Surg Aug 2011;213(2):236-44, 2011 Aug  <https://dx.doi.org/10.1016/j.jamcollsurg.2011.04.008> | Does not report (internal) validation performance metric(s) |
| 252. | Lee MJ; Cizik AM; Hamilton D; Chapman JR, Predicting surgical site infection after spine surgery: a validated model using a prospective surgical registry. Spine J Sep 2014;14(9):2112-7, United States 2014 Sep.  <https://dx.doi.org/10.1016/j.spinee.2013.12.026> | Timepoint infection not specified |
| 253. | Lemmens J; Klarenbeek B; Verstegen M; van Workum F; Hannink G; Ubels S; Rosman C, Performance of a consensus-based algorithm for diagnosing anastomotic leak after minimally invasive esophagectomy for esophageal cancer. Dis Esophagus Mar 2023;(): United States 2023 Mar  <https://dx.doi.org/10.1093/dote/doad016> | Timepoint infection not specified |
| 254. | Lepelletier, D.; Perron, S.; Bizouarn, P.; Caillon, J.; Drugeon, H.; Michaud, J. L.; Duveau, D., Surgical site infection predictive model and impact of patient skin preparation after cardiac surgery, International Journal of Antimicrobial Agents Dec 2004;24():S226-S227, 2004 Dec | Article not available |
| 255. | Levi B; Zhang P; Lisiecki J; Terjimanian MN; Rinkinen J; Agarwal S; Holcombe SA; Kozlow JH; Wang SC; Kuzon WM, Use of morphometric assessment of body composition to quantify risk of surgical-site infection in patients undergoing component separation ventral hernia repair. Plast Reconstr Surg Apr 2014;133(4):559e-566e, United States 2014 Apr.  <https://dx.doi.org/10.1097/PRS.0000000000000009> | Timepoint infection not specified |
| 256. | Lewallen LW; Maradit Kremers H; Lahr BD; Mabry TM; Steckelberg JM; Berry DJ; Hanssen AD; Berbari EF; Osmon DR, External validation of the national healthcare safety network risk models for surgical site infections in total hip and knee replacements. Infect Control Hosp Epidemiol Nov 2014;35(11):1323-9, United States 2014 Nov.  <https://dx.doi.org/10.1086/678412> | Timepoint infection not in prediction window |
| 257. | Li MP; Liu WC; Wu JB; Luo K; Liu Y; Zhang Y; Xiao SN; Liu ZL; Huang SH; Liu JM, Machine learning for the prediction of postoperative nosocomial pulmonary infection in patients with spinal cord injury. Eur Spine J May 2023;(): Germany 2023 May.  <https://dx.doi.org/10.1007/s00586-023-07772-8> | Timepoint infection not specified |
| 258. | Li P; Wang Y; Li H; Cheng B; Wu S; Ye H; Ma D; Fang X, Prediction of postoperative infection in elderly using deep learning-based analysis: an observational cohort study. Aging Clin Exp Res Mar 2023;35(3):639-647, Germany 2023 Mar.  <https://dx.doi.org/10.1007/s40520-022-02325-3> | Timepoint infection not in prediction window |
| 259. | Li R; Zhou J; Zhao S; Sun Q; Wang D, Prediction model of anastomotic leakage after anterior resection for rectal cancer-based on nomogram and multivariate analysis with 1995 patients. Int J Colorectal Dis May 2023;38(1):139, Germany 2023 May  <https://dx.doi.org/10.1007/s00384-023-04438-1> | Timepoint infection not specified |
| 260. | Li, S.; Su, J.; Sui, Q.; Wang, G., A nomogram for predicting postoperative pulmonary infection in esophageal cancer patients. BMC Pulm Med Sep 6 2021;21(1):283, 2021 Sep 6  <https://dx.doi.org/10.1186/s12890-021-01656-7> | Timepoint infection not specified |
| 261. | Li T; Huang J; Lei P; Yang X; Chen Z; Chen P; Zhai J; Guo X; Wei H, A novel nomogram for anastomotic leakage after surgery for rectal cancer: a retrospective study. PeerJ 2022;10():e14437, United States 2022.  <https://dx.doi.org/10.7717/peerj.14437> | Timepoint infection not specified |
| 262. | Li T; Sun XZ; Lai DH; Li X; He YZ, Fever and systemic inflammatory response syndrome after retrograde intrarenal surgery: Risk factors and predictive model. Kaohsiung J Med Sci Jul 2018;34(7):400-408, China (Republic : 1949- ) 2018 Jul  <https://dx.doi.org/10.1016/j.kjms.2018.01.002> | Wrong outcomes |
| 263. | Li X; Nylander W; Smith T; Han S; Gunnar W, Risk Factors and Predictive Model Development of Thirty-Day Post-Operative Surgical Site Infection in the Veterans Administration Surgical Population. Surg Infect (Larchmt) Apr 2018;19(3):278-285, United States 2018 Apr.  <https://dx.doi.org/10.1089/sur.2017.283> | Timepoint infection not in prediction window |
| 264. | Li, Y.; Ma, Y. L.; Gao, Y. Y.; Wang, D. D.; Chen, Q., Analysis of the risk factors of postoperative cardiopulmonary complications and ability to predicate the risk in patients after lung cancer surgery. Journal of Thoracic Disease Jun 2017;9(6):1565-1573, 2017 Jun.  <https://dx.doi.org/10.21037/jtd.2017.05.42> | Does not report (internal) validation performance metric(s) |
| 265. | Li Y; Zhang J; He Z, Early Predictive Value of Procalcitonin for the Diagnosis of Pulmonary Infections after Off-pump Coronary Artery Bypass Grafting. Heart Surg Forum Jan 2021;24(1):E004-E008, United States 2021 Jan.  <https://dx.doi.org/10.1532/hsf.3381> | Wrong study design |
| 266. | Liang, M.; Kao, L.; Martindale, R.; Roth, S., External validation of the ventral hernia risk score for predicting surgical site infections, Surgical Infections April 2014;15():S-20, 2014 April.  <https://dx.doi.org/10.1089/sur.2014.9990.abstracts> | Article not available |
| 267. | Liao, C.; Aziz, M.; Chiu, C. Y.; Mari, G.; Tate, D. L., Predicting post-cesarean surgical site infections: A secondary analysis of the consortium on safe labor, Obstetrics and Gynecology 2020;135 (Supplement 1)():174S, 2020. | Conference abstract |
| 268. | Lindenmann J; Fink-Neuboeck N; Porubsky C; Fediuk M; Anegg U; Kornprat P; Smolle M; Maier A; Smolle J; Smolle-Juettner FM, A nomogram illustrating the probability of anastomotic leakage following cervical esophagogastrostomy. Surg Endosc Oct 2020;(): Germany 2020 Oct.  <https://dx.doi.org/10.1007/s00464-020-08107-0> | Timepoint infection not specified |
| 269. | Liu, J.; Yang, Q.; Lan, J.; Hong, Y.; Huang, X.; Yang, B. Risk factors and prediction model of urosepsis in patients with diabetes after percutaneous nephrolithotomy. BMC Urol Apr 28 2021;21(1):74, 2021 Apr 28  <https://dx.doi.org/10.1186/s12894-021-00799-3> | Timepoint infection not specified |
| 270. | Liu, Q.; Fu, C.; Zhang, W. The anastomotic leak risk score (ALRS): A model to predict the risk of anastomotic leakage, Diseases of the Colon and Rectum May 2016;59 (5)():e203, 2016 May.  <https://dx.doi.org/10.1097/01.dcr.0000482708.50838.af> | Conference abstract |
| 271. | Liu X; Kelleners-Smeets NWJ; Sprengers M; Hira V; Mosterd K; Nelemans PJ, A Clinical Prediction Model for Surgical Site Infections in Dermatological Surgery. Acta Derm Venereol Jul 2018;98(7):683-688, Sweden 2018 Jul  <https://dx.doi.org/10.2340/00015555-2945> | Wrong patient population |
| 272. | Liu, Y.; Lu, J.; Xiao, C.; Ma, L., A predicting model based on risk factors for urosepsis after one-phase percutaneous nephrolithotomy, Journal of Urology April 2018;199 (4 Supplement 1)():e124-e125, 2018 April. | Conference abstract |
| 273. | Liu, Y. Q.; Lu, J.; Hao, Y. C.; Xiao, C. L.; Ma, L. L., Predicting model based on risk factors for urosepsis after percutaneous nephrolithotomy, Beijing Da Xue Xue Bao Yi Xue Ban Jun 18 2018;50(3):507-513, 2018 Jun 18. | Not in English |
| 274. | Liu Y; Wan X; Wang G; Ren Y; Cheng Y; Zhao Y; Han G, A scoring system to predict the risk of anastomotic leakage after anterior resection for rectal cancer. J Surg Oncol Feb 2014;109(2):122-5, United States 2014 Feb.  <https://dx.doi.org/10.1002/jso.23467> | Does not report (internal) validation performance metric(s) |
| 275. | Liu Z; Dai T; Wang Z; Zhang Z; Qiu W; He Y, Nomogram model to predict postoperative infection after mandibular osteoradionecrosis surgery. Sci Rep Jun 2017;7(1):3479, 2017 Jun  <https://dx.doi.org/10.1038/s41598-017-03672-2> | Timepoint infection not specified |
| 276. | Liu Z; Wu H; Liufu N; Cheng S; Huang H; Hu C; Cao M, Development and validation of a nomogram incorporating selected systemic inflammation-based prognostic marker for complication prediction after vascularized fibula flap reconstruction. Oral Oncol Dec 2019;99():104467, England 2019 Dec  <https://dx.doi.org/10.1016/j.oraloncology.2019.104467> | Wrong outcomes |
| 277. | Lobato, L. F.; Wells, B.; Wick, E.; Pronty, K. T.; Kiran, P. R.; Remzi, F. H.; Vogel, J. D., Predicting organ space surgical site infection with a nomogram, Gastroenterology May 2009;136(5):A884-A885, 2009 May.  <https://dx.doi.org/10.1016/S0016-5085> | Duplicate (exact similar publication in other journal) |
| 278. | Loftus, T. J.; Giordano, C.; Upchurch, G. R.; Bihorac, A., Artificial Intelligence for Predicting Complication with Live-Streaming Data: Prospective MySurgeryRisk Validation, Journal of the American College of Surgeons October 2020;231 (4 Supplement 1)():S132-S133, 2020 October.  <https://dx.doi.org/10.1016/j.jamcollsurg.2020.07.256> | Conference abstract |
| 279. | Lohmann, S.; Brix, T.; Varghese, J.; Warneke, N.; Schwake, M.; Suero Molina, E.; Holling, M.; Stummer, W.; Schipmann, S., Development and validation of prediction scores for nosocomial infections, reoperations, and adverse events in the daily clinical setting of neurosurgical patients with cerebral and spinal tumors, J Neurosurg Mar 20 2020;134(3):1226-1236, 2020 Mar 20.  <https://dx.doi.org/10.3171/2020.1.Jns193186> | Timepoint infection not in prediction window |
| 280. | Loosen SH; Breuer A; Tacke F; Kather JN; Gorgulho J; Alizai PH; Bednarsch J; Roeth AA; Lurje G; Schmitz SM; Brozat JF; Paffenholz P; Vucur M; Ritz T; Koch A; Trautwein C; Ulmer TF; Roderburg C; Longerich T; Neumann UP; Luedde T, Circulating levels of soluble urokinase plasminogen activator receptor predict outcome after resection of biliary tract cancer. JHEP Rep Apr 2020;2(2):100080, 2020 Apr.  <https://dx.doi.org/10.1016/j.jhepr.2020.100080> | Wrong outcomes |
| 281. | Lopez CD; Gazgalis A; Peterson JR; Confino JE; Levine WN; Popkin CA; Lynch TS, Machine Learning Can Accurately Predict Overnight Stay, Readmission, and 30-Day Complications Following Anterior Cruciate Ligament Reconstruction. Arthroscopy Mar 2023;39(3):777-786.e5, United States 2023 Mar.  <https://dx.doi.org/10.1016/j.arthro.2022.06.032> | Publication date |
| 282. | Lorenzi, E.; Henao, R.; Heller, K., HIERARCHICAL INFINITE FACTOR MODELS FOR IMPROVING THE PREDICTION OF SURGICAL COMPLICATIONS FOR GERIATRIC PATIENTS, Annals of Applied Statistics Dec 2019;13(4):2637-2661, 2019 Dec.  <https://dx.doi.org/10.1214/19-aoas1292> | Wrong outcomes |
| 283. | Lu G; Liu Y; Huang Y; Ding J; Zeng Q; Zhao L; Li M; Yu H; Li Y, Prediction model of central nervous system infections in patients with severe traumatic brain injury after craniotomy. J Hosp Infect Jun 2023;136():90-99, England 2023 Jun.  <https://dx.doi.org/10.1016/j.jhin.2023.04.004> | Timepoint infection not specified |
| 284. | Lu, J.; Zheng, L.; Li, R.; Hao, C.; Gao, W.; Feng, Z.; Yin, G.; Wang, Y., Diagnostic value of dynamic monitoring of C-reactive protein in drain drainage to predict early anastomotic leakage after colorectal cancer surgery, Zhonghua Wei Chang Wai Ke Za Zhi Sep 25 2017;20(9):1055-1059, 2017 Sep 25. | Not in English |
| 285. | Lu, K.; Ma, T.; Yang, C.; Qu, Q.; Liu, H., Risk prediction model for deep surgical site infection (DSSI) following open reduction and internal fixation of displaced intra-articular calcaneal fracture, Int Wound J Mar 2022;19(3):656-665, 2022 Mar.  <https://dx.doi.org/10.1111/iwj.13663> | Does not report (internal) validation performance metric(s) |
| 286. | Lu, S. Q.; Chang, X. F.; Yang, X. D.; Yu, D. C.; Huang, Q. G.; Wang, F., Establishment of a nomogram predicting risk factors of postoperative perineal wound complications after abdominoperineal resection for rectal cancer, Zhonghua Wei Chang Wai Ke Za Zhi Apr 25 2019;22(4):357-363, 2019 Apr 25.  <https://dx.doi.org/10.3760/cma.j.issn.1671-0274.2019.04.008> | Not in English |
| 287. | Lubelski D; Alentado V; Nowacki AS; Shriver M; Abdullah KG; Steinmetz MP; Benzel EC; Mroz TE, Preoperative Nomograms Predict Patient-Specific Cervical Spine Surgery Clinical and Quality of Life Outcomes. Neurosurgery Jul 2018;83(1):104-113, United States 2018 Jul.  <https://dx.doi.org/10.1093/neuros/nyx343> | Wrong outcomes |
| 288. | Lubelski, D.; Feghali, J.; Ehresman, J.; Pennington, Z.; Schilling, A.; Huq, S.; Medikonda, R.; Theodore, N.; Sciubba, D. M., Web-Based Calculator Predicts Surgical-Site Infection After Thoracolumbar Spine Surgery, World Neurosurg Jul 2021;151():e571-e578, 2021 Jul.  <https://dx.doi.org/10.1016/j.wneu.2021.04.086> | Timepoint infection not in prediction window |
| 289. | Luo K; Huang YQ; Zhu LB; Gan XR; Zhang Y; Xiao SN; Zhou RP; Chen JW; Liu JM; Liu ZL, Risk Factors and Nomogram for Postoperative Pulmonary Infection in Patients with Cervical Spinal Cord Injury. World Neurosurg Jun 2023;(): United States 2023 Jun.  <https://dx.doi.org/10.1016/j.wneu.2023.06.040> | Timepoint infection not specified |
| 290. | Luo Y; Tang Z; Hu X; Lu S; Miao B; Hong S; Bai H; Sun C; Qiu J; Liang H; Na N, Machine learning for the prediction of severe pneumonia during posttransplant hospitalization in recipients of a deceased-donor kidney transplant. Ann Transl Med Feb 2020;8(4):82, 2020 Feb.  <https://dx.doi.org/10.21037/atm.2020.01.09> | Timepoint infection not specified |
| 291. | Luz CF; Vollmer M; Decruyenaere J; Nijsten MW; Glasner C; Sinha B, Machine learning in infection management using routine electronic health records: tools, techniques, and reporting of future technologies. Clin Microbiol Infect Oct 2020;26(10):1291-1299, England 2020 Oct.  <https://dx.doi.org/10.1016/j.cmi.2020.02.003> | Wrong study design |
| 292. | Ma, K. W.; Cheung, T. T.; She, W. H.; Chok, K. S. H.; Chan, A. C. Y.; Dai, W. C.; Lo, C. M., Risk prediction model for major complication after hepatectomy for malignant tumour - A validated scoring system from a university center, Surgical Oncology-Oxford Dec 2017;26(4):446-452, 2017 Dec.  <https://dx.doi.org/10.1016/j.suronc.2017.08.007> | Wrong outcomes |
| 293. | Ma R; He J; Xu B; Zhao C; Zhang Y; Li X; Sun S; Zhang Q, Nomogram prediction of surgical site infection of HIV-infected patients following orthopedic surgery: a retrospective study. BMC Infect Dis Nov 2020;20(1):896, 2020 Nov.  <https://dx.doi.org/10.1186/s12879-020-05613-3> | Timepoint infection not in prediction window |
| 294. | Ma, T.; Li, Q. S.; Wang, Y.; Wang, B.; Wu, Z.; Lv, Y.; Wu, R. Q., Value of pretransplant albumin-bilirubin score in predicting outcomes after liver transplantation, World J Gastroenterol Apr 21 2019;25(15):1879-1889, 2019 Apr 21.  <https://dx.doi.org/10.3748/wjg.v25.i15.1879> | Wrong outcomes |
| 295. | Machida H; Hom MS; Shabalova A; Grubbs BH; Matsuo K, Predictive model of urinary tract infection after surgical treatment for women with endometrial cancer. Arch Gynecol Obstet Aug 2017;296(2):335-343, 2017 Aug.  <https://dx.doi.org/10.1007/s00404-017-4434-5> | Does not report (internal) validation performance metric(s) |
| 296. | Magboo R; Drey N; Cooper J; Byers H; Shipolini A; Sanders J, Predicting cardiac surgical site infection: development and validation of the Barts Surgical Infection Risk tool. J Clin Epidemiol Dec 2020;128():57-65, United States 2020 Dec.  <https://dx.doi.org/10.1016/j.jclinepi.2020.08.015> | Timepoint infection not specified |
| 297. | Mahmud N; Fricker Z; Lewis JD; Taddei TH; Goldberg DS; Kaplan DE, Risk Prediction Models for Postoperative Decompensation and Infection in Patients With Cirrhosis: A Veterans Affairs Cohort Study. Clin Gastroenterol Hepatol May 2022;20(5):e1121-e1134, United States 2022 May.  <https://dx.doi.org/10.1016/j.cgh.2021.06.050> | Timepoint infection not in prediction window |
| 298. | Makino Y; Ishida K; Kishi K; Kodama H; Miyawaki T, The association between surgical complications and the POSSUM score in head and neck reconstruction: a retrospective single-center study. J Plast Surg Hand Surg Jun 2018;52(3):153-157, Sweden 2018 Jun.  <https://dx.doi.org/10.1080/2000656X.2017.1372288> | Wrong outcomes |
| 299. | Mansukhani V; Desai G; Shah R; Jagannath P, The role of preoperative C-reactive protein and procalcitonin as predictors of post-pancreaticoduodenectomy infective complications: A prospective observational study. Indian J Gastroenterol Jul 2017;36(4):289-295, India 2017 Jul.  <https://dx.doi.org/10.1007/s12664-017-0770-4> | Does not report (internal) validation performance metric(s) |
| 300 | Mao, C. C.; Chen, X. D.; Lin, J.; Zhu-Ge, W. S.; Xie, Z. D.; Chen, X. Y.; Zhang, F. M.; Wu, R. S.; Zhang, W. T.; Lou, N.; Shi, L.; Zhu, G. B.; Shen, X., A Novel Nomogram for Predicting Postsurgical Intra-abdominal Infection in Gastric Cancer Patients: a Prospective Study, J Gastrointest Surg Mar 2018;22(3):421-429, 2018 Mar.  <https://dx.doi.org/10.1007/s11605-017-3580-1> | Timepoint infection not specified |
| 301. | Mao, Y. S.; Hao, S. J.; Zou, C. F.; Xie, Z. B.; Fu, D. L., Controlling Nutritional Status score is superior to Prognostic Nutritional Index score in predicting survival and complications in pancreatic ductal adenocarcinoma: a Chinese propensity score matching study, Br J Nutr Dec 14 2020;124(11):1190-1197, 2020 Dec 14.  <https://dx.doi.org/10.1017/s0007114520002299> | Does not report (internal) validation performance metric(s) |
| 302. | Martin, G.; Dupré, A.; Mulliez, A.; Prunel, F.; Slim, K.; Pezet, D., Validation of a score for the early diagnosis of anastomotic leakage following elective colorectal surgery, J Visc Surg Feb 2015;152(1):5-10, 2015 Feb.  <https://dx.doi.org/10.1016/j.jviscsurg.2014.12.002> | Timepoint infection not specified |
| 303. | Marubashi S; Ichihara N; Kakeji Y; Miyata H; Taketomi A; Egawa H; Takada Y; Umeshita K; Seto Y; Gotoh M, "Real-time" risk models of postoperative morbidity and mortality for liver transplants. Ann Gastroenterol Surg Jan 2019;3(1):75-95, 2019 Jan.  <https://dx.doi.org/10.1002/ags3.12217> | Wrong outcomes |
| 304. | Matsumoto, H.; Campbell, M.; Minkara, A.; Roye, D. P.; Garg, S.; Johnston, C.; Samdani, A.; Smith, J.; Sponseller, P.; Sturm, P. F.; Vitale, M., Paper #45: Development of a Risk Severity Score (RSS) Predicting Surgical Site Infection in Early Onset Scoliosis: Identifying High-Risk Patients, Spine Deformity 01 Nov 2017;5(6):464-465, 2017 01 Nov.  <https://dx.doi.org/10.1016/j.jspd.2017.09.048> | Conference abstract |
| 305. | Medic G; Kosaner Kließ M; Atallah L; Weichert J; Panda S; Postma M; El-Kerdi A, Evidence-based Clinical Decision Support Systems for the prediction and detection of three disease states in critical care: A systematic literature review. F1000Res 2019;8():1728, 2019.  <https://dx.doi.org/10.12688/f1000research.20498.2> | Wrong outcomes |
| 306. | Medline A; Muralidharan VJ; Codner J; Sharma J, Organ-Space Surgical Site Infections: Consequences and Prediction Using ACS-NSQIP. Am Surg Aug 2022;88(8):1773-1782, United States 2022 Aug.  <https://dx.doi.org/10.1177/00031348221083944> | Timepoint infection not specified |
| 307. | Meltzer AJ; Graham A; Connolly PH; Meltzer EC; Karwowski JK; Bush HL; Schneider DB, The Comprehensive Risk Assessment for Bypass (CRAB) facilitates efficient perioperative risk assessment for patients with critical limb ischemia. J Vasc Surg May 2013;57(5):1186-95, United States 2013 May.  <https://dx.doi.org/10.1016/j.jvs.2012.09.083> | Wrong outcomes |
| 308. | Merkow, R. P.; Kmiecik, T. E.; Bentrem, D. J.; Winchester, D. P.; Stewart, A. K.; Ko, C. Y.; Bilimoria, K. Y., Effect of including cancer-specific variables on models examining short-term outcomes, Cancer Apr 2013;119(7):1412-1419, 2013 Apr.  <https://dx.doi.org/10.1002/cncr.27891> | Does not report (internal) validation performance metric(s) |
| 309. | Meyer ZC; Schreinemakers JM; de Waal RA; van der Laan L, Searching for predictors of surgical complications in critically ill surgery patients in the intensive care unit: a review. Surg Today Sep 2015;45(9):1091-101, Japan 2015 Sep.  <https://dx.doi.org/10.1007/s00595-015-1159-6> | Wrong study design |
| 310. | Miholic J; Hudec M; Müller MM; Domanig E; Wolner E, Early prediction of deep sternal wound infection after heart operations by alpha-1 acid glycoprotein and C-reactive protein measurements. Ann Thorac Surg Oct 1986;42(4):429-33, Netherlands 1986 Oct.  <https://dx.doi.org/10.1016/s0003-4975(10)60552-8> | Timepoint infection not in prediction window |
| 311. | Mikkelsen MM; Andersen NH; Christensen TD; Hansen TK; Eiskjaer H; Gjedsted J; Johnsen SP; Hjortdal VE, Microalbuminuria is associated with high adverse event rate following cardiac surgery. Eur J Cardiothorac Surg Jun 2011;39(6):932-8, Germany 2011 Jun.  <https://dx.doi.org/10.1016/j.ejcts.2010.09.043> | Does not report (internal) validation performance metric(s) |
| 312. | Mikkelsen MM; Andersen NH; Christensen TD; Hansen TK; Eiskjaer H; Mogensen CE; Hjortdal VE; Johnsen SP, Microalbuminuria and short-term prognosis in patients undergoing cardiac surgery., Interact Cardiovasc Thorac Surg Sep 2009;9(3):484-90, England 2009 Sep.  <https://dx.doi.org/10.1510/icvts.2009.203836> | Timepoint infection not specified |
| 313. | Miyata H; Motomura N; Tsukihara H; Takamoto S, Risk models including high-risk cardiovascular procedures: clinical predictors of mortality and morbidity. Eur J Cardiothorac Surg May 2011;39(5):667-74, Germany 2011 May.  <https://dx.doi.org/10.1016/j.ejcts.2010.08.050> | Wrong outcomes |
| 314. | Mohri Y; Miki C; Kobayashi M; Okita Y; Inoue M; Uchida K; Tanaka K; Inoue Y; Kusunoki M, Correlation between preoperative systemic inflammation and postoperative infection in patients with gastrointestinal cancer: a multicenter study. Surg Today May 2014;44(5):859-67, Japan 2014 May.  <https://dx.doi.org/10.1007/s00595-013-0622-5> | Does not report (internal) validation performance metric(s) |
| 315. | Mok JM; Pekmezci M; Piper SL; Boyd E; Berven SH; Burch S; Deviren V; Tay B; Hu SS, Use of C-reactive protein after spinal surgery: comparison with erythrocyte sedimentation rate as predictor of early postoperative infectious complications. Spine (Phila Pa 1976) Feb 2008;33(4):415-21, United States 2008 Feb.  <https://dx.doi.org/10.1097/BRS.0b013e318163f9ee> | Timepoint infection not specified |
| 316. | Montewka, M.; Rudzki, S.; Plewik, D.; Koziol-Montewka, M.; Skrzek, A., Innovative define of Bayesian model to assess the risk of surgical site infection in patients undergoing abdominal surgery, International Journal of Infectious Diseases April 2014;21():281, 2014 April.  <https://dx.doi.org/10.1016/j.ijid.2014.03.1003> | Conference abstract |
| 317. | Moreno Elola-Olaso A; Davenport DL; Hundley JC; Daily MF; Gedaly R, Predictors of surgical site infection after liver resection: a multicentre analysis using National Surgical Quality Improvement Program data. HPB (Oxford) Feb 2012;14(2):136-41, England 2012 Feb.  <https://dx.doi.org/10.1111/j.1477-2574.2011.00417.x> | Does not report (internal) validation performance metric(s) |
| 318. | Morey VM; Song YD; Whang JS; Kang YG; Kim TK, Can Serum Albumin Level and Total Lymphocyte Count be Surrogates for Malnutrition to Predict Wound Complications After Total Knee Arthroplasty? J Arthroplasty Jun 2016;31(6):1317-1321, United States 2016 Jun.  <https://dx.doi.org/10.1016/j.arth.2015.12.004> | Timepoint infection not in prediction window |
| 319. | Mortazavi BJ; Desai N; Zhang J; Coppi A; Warner F; Krumholz HM; Negahban S, Prediction of Adverse Events in Patients Undergoing Major Cardiovascular Procedures. IEEE J Biomed Health Inform Nov 2017;21(6):1719-1729, United States 2017 Nov.  <https://dx.doi.org/10.1109/JBHI.2017.2675340> | Timepoint infection not specified |
| 320. | Moulton, L. J.; Jelovsek, J. E.; Lachiewicz, M.; Chagin, K.; Goje, O., A model to predict risk of infection after cesarean delivery, Obstetrics and Gynecology May 2016;127 (Supplement 1)():85S, 2016 May.  <https://dx.doi.org/10.1097/01.AOG.0000483785.85498.7b> | Conference abstract |
| 321. | Moulton LJ; Eric Jelovsek J; Lachiewicz M; Chagin K; Goje O, A model to predict risk of postpartum infection after Caesarean delivery. J Matern Fetal Neonatal Med Sep 2018;31(18):2409-2417, England 2018 Sep.  <https://dx.doi.org/10.1080/14767058.2017.1344632> | Timepoint infection not in prediction window |
| 322. | Mu Y; Edwards JR; Horan TC; Berrios-Torres SI; Fridkin SK, Improving risk-adjusted measures of surgical site infection for the national healthcare safety network. Infect Control Hosp Epidemiol Oct 2011;32(10):970-86, United States 2011 Oct.  <https://dx.doi.org/10.1086/662016> | Timepoint infection not in prediction window |
| 323. | Mueller KB; Hou Y; Beach K; Griffin LP, Development and validation of a point-of-care clinical risk score to predict surgical site infection following open spinal fusion. N Am Spine Soc J Mar 2023;13():100196, United States 2023 Mar.  <https://dx.doi.org/10.1016/j.xnsj.2022.100196> | Timepoint infection not in prediction window |
| 324. | Mujagic, E.; Marti, W. R.; Coslovsky, M.; Zeindler, J.; Staubli, S.; Marti, R.; Mechera, R.; Soysal, S. D.; Gürke, L.; Weber, W. P., The role of preoperative blood parameters to predict the risk of surgical site infection, American Journal of Surgery 2018;215(4):651‐657, 2018.  <https://dx.doi.org/10.1016/j.amjsurg.2017.08.021> | Does not report (internal) validation performance metric(s) |
| 325. | Mulder T; Kluytmans-van den Bergh MFQ; van Mourik MSM; Romme J; Crolla RMPH; Bonten MJM; Kluytmans JAJW, A diagnostic algorithm for the surveillance of deep surgical site infections after colorectal surgery. Infect Control Hosp Epidemiol May 2019;40(5):574-578, 2019 May.  <https://dx.doi.org/10.1017/ice.2019.36> | Wrong outcomes |
| 326. | Murphy, M. M.; Shah, S. A.; Simons, J. P.; Csikesz, N.; Ng, S. C.; Zhou, Z.; Tseng, J. F., Major complications after laparoscopic cholecystectomy:A simple risk score, Gastroenterology May 2009;136(5):A885-A886, 2009 May.  <https://dx.doi.org/10.1016/S0016-5085> | Timepoint infection not in prediction window |
| 327. | Murphy MM; Shah SA; Simons JP; Csikesz NG; McDade TP; Bodnari A; Ng SC; Zhou Z; Tseng JF, Predicting major complications after laparoscopic cholecystectomy: a simple risk score. J Gastrointest Surg Nov 2009;13(11):1929-36, United States 2009 Nov.  <https://dx.doi.org/10.1007/s11605-009-0979-3> | Wrong outcomes |
| 328. | Nadal, L. R.; Boustani, S. E.; Johann, L.; Medeiros Da Silva, A.; Medrado, M. B.; Lupinacci, R. A.; Farah, J. F., Pre-operative cross-sectional measurement of bilateral psoas muscle in computerized tomography to estimate the risk of postoperative infectious or anastomotic complications of colorectal resection. Colorectal Disease September 2019;21 (Supplement 3)():46-47, 2019 September. | Conference abstract |
| 329. | Nakazawa, K.; Ishikawa, T.; Toyama, A.; Wakai, T.; Akazawa, K., Prediction of postoperative infection for patients undergoing gastrointestinal surgery: Findings from electronic health records, Gastroenterology Insights 2020;11(2): 2020.  <https://dx.doi.org/10.3390/GASTROENT11020007> | Timepoint infection not specified |
| 330. | Namba T; Ueno M; Inoue G; Imura T; Saito W; Nakazawa T; Miyagi M; Shirasawa E; Takahashi O; Takaso M, Prediction tool for high risk of surgical site infection in spinal surgery. Infect Control Hosp Epidemiol Jul 2020;41(7):799-804, United States 2020 Jul.  <https://dx.doi.org/10.1017/ice.2020.107> | Timepoint infection not specified |
| 331. | Nemeth, S. K.; Simon, H. L.; Keller, D. S., Creation of a Optimized Risk Model for Morbidity and Mortality in Colectomy, Journal of the American College of Surgeons October 2020;231 (4 Supplement 2)():e94-e95, 2020 October.  <https://dx.doi.org/10.1016/j.jamcollsurg.2020.08.239> | Conference abstract |
| 332. | Nguyen, J.; Ferguson, S.; Bernardini, M.; May, T.; Laframboise, S.; Hogen, L.; Bouchard-Fortier, G., Preoperative neutrophil-to-lymphocyte ratio: A predictor of 30-day postoperative complications after primary surgery for ovarian cancer, International Journal of Gynecological Cancer September 2018;28 (Supplement 2)():60, 2018 September.  <https://dx.doi.org/10.1097/01.IGC.0000546279.09648.02> | Wrong outcomes |
| 333. | Nie H; Jiang D; Ou Y; Quan Z; Hao J; Bai C; Huang X; An H, Procalcitonin as an early predictor of postoperative infectious complications in patients with acute traumatic spinal cord injury. Spinal Cord Jun 2011;49(6):715-20, England 2011 Jun.  <https://dx.doi.org/10.1038/sc.2010.190> | Timepoint infection not specified |
| 334. | Nieto-Cabrera M; Fernández-Pérez C; García-González I; Martin-Benítez JC; Ferrero J; Bringas M; Carnero M; Maroto L; Sánchez-García M, Med-Score 24: A multivariable prediction model for poststernotomy mediastinitis 24 hours after admission to the intensive care unit. J Thorac Cardiovasc Surg Mar 2018;155(3):1041-1051.e5, United States 2018 Mar.  <https://dx.doi.org/10.1016/j.jtcvs.2017.09.160> | Timepoint infection not specified |
| 335. | Noble F; Curtis N; Harris S; Kelly JJ; Bailey IS; Byrne JP; Underwood TJ, Risk assessment using a novel score to predict anastomotic leak and major complications after oesophageal resection. J Gastrointest Surg Jun 2012;16(6):1083-95, United States 2012 Jun.  <https://dx.doi.org/10.1007/s11605-012-1867-9> | Timepoint infection not specified |
| 336. | Noorit P; Siribumrungwong B; Thakkinstian A, Clinical prediction score for superficial surgical site infection after appendectomy in adults with complicated appendicitis. World J Emerg Surg 2018;13():23, 2018.  <https://dx.doi.org/10.1186/s13017-018-0186-1> | Timepoint infection not in prediction window |
| 337. | Obeid, N. M.; Rubinfeld, I. S.; Kwon, D. S.; Schmoekel, N. H.; Kather, R.; Velanovich, V., Age deciles contribute to risk stratification of short-term pancreatectomy morbidity and mortality, Journal of the American College of Surgeons September 2013;217(3):S60, 2013 September.  <https://dx.doi.org/10.1016/j.jamcollsurg.2013.07.126> | Conference abstract |
| 338. | Okugawa Y; Toiyama Y; Yamamoto A; Shigemori T; Ide S; Kitajima T; Fujikawa H; Yasuda H; Hiro J; Yoshiyama S; Yokoe T; Saigusa S; Tanaka K; Shirai Y; Kobayashi M; Ohi M; Araki T; McMillan DC; Miki C; Goel A; Kusunoki M, Lymphocyte-C-reactive Protein Ratio as Promising New Marker for Predicting Surgical and Oncological Outcomes in Colorectal Cancer. Ann Surg Aug 2020;272(2):342-351, United States 2020 Aug.  <https://dx.doi.org/10.1097/SLA.0000000000003239> | Timepoint infection not specified |
| 339. | Okui J; Ueno R; Matsui H; Uegami W; Hayashi H; Miyajima T; Kusanagi H, Early prediction model of organ/space surgical site infection after elective gastrointestinal or hepatopancreatobiliary cancer surgery. J Infect Chemother Sep 2020;26(9):916-922, Netherlands 2020 Sep.  <https://dx.doi.org/10.1016/j.jiac.2020.04.009> | Timepoint infection not in prediction window |
| 340. | Oliveira, M.; Rodrigues, D.; Valerio, M.; Brito, P. U.; Rodrigues, C., Cardiopulmonary Exercise Testing predicting postoperative morbimortality, European Respiratory Journal. Conference: 29th International Congress of the European Respiratory Society, ERS. Madrid Spain 2019;54(Supplement 63): 2019.  <https://dx.doi.org/10.1183/13993003.congress-2019.PA4138> | Conference abstract |
| 341. | Olsen, M. A.; Nickel, K. B.; Margenthaler, J. A.; Fox, I. K.; Wallace, A. E.; Fraser, V. J., Development of a single model to predict surgical site infection and non-infectious wound complications after mastectomy with immediate reconstruction, Open Forum Infectious Diseases. Conference: ID Week 2016;3(Supplement 1): 2016  <https://dx.doi.org/10.1093/ofid/ofw172.1065> | Conference abstract |
| 342. | Olsen MA; Higham-Kessler J; Yokoe DS; Butler AM; Vostok J; Stevenson KB; Khan Y; Fraser VJ, Developing a risk stratification model for surgical site infection after abdominal hysterectomy. Infect Control Hosp Epidemiol Nov 2009;30(11):1077-83, 2009 Nov.  <https://dx.doi.org/10.1086/606166> | Wrong study design |
| 343. | Olsen MA; Nickel KB; Margenthaler JA; Fox IK; Ball KE; Mines D; Wallace AE; Colditz GA; Fraser VJ, Development of a Risk Prediction Model to Individualize Risk Factors for Surgical Site Infection After Mastectomy. Ann Surg Oncol Aug 2016;23(8):2471-9, 2016 Aug.  <https://dx.doi.org/10.1245/s10434-015-5083-1> | Timepoint infection not in prediction window |
| 344. | Oosterhoff, J. H. F.; Gravesteijn, B. Y.; Karhade, A. V.; Jaarsma, R. L.; Kerkhoffs, Gmmj; Ring, D.; Schwab, J. H.; Steyerberg, E. W.; Doornberg, J. N., Feasibility of Machine Learning and Logistic Regression Algorithms to Predict Outcome in Orthopaedic Trauma Surgery, J Bone Joint Surg Am Dec 17 2021;(): 2021 Dec 17.  <https://dx.doi.org/10.2106/jbjs.21.00341> | Timepoint infection not specified |
| 345. | Oosterhoff JHF; Gravesteijn BY; Karhade AV; Jaarsma RL; Kerkhoffs GMMJ; Ring D; Schwab JH; Steyerberg EW; Doornberg JN, Feasibility of Machine Learning and Logistic Regression Algorithms to Predict Outcome in Orthopaedic Trauma Surgery. J Bone Joint Surg Am Mar 2022;104(6):544-551, United States 2022 Mar.  <https://dx.doi.org/10.2106/JBJS.21.00341> | Timepoint infection not specified |
| 346. | Orfanoudaki, A.; Giannoutsou, A.; Hashim, S.; Bertsimas, D.; Hagberg, R. C., Machine learning models for mitral valve replacement: A comparative analysis with the Society of Thoracic Surgeons risk score, J Card Surg Jan 2022;37(1):18-28, 2022 Jan.  <https://dx.doi.org/10.1111/jocs.16072> | Timepoint infection not specified |
| 347. | Orozco-Martin, V.; Orjuela, J. O.; Aragon, A. E. R.; Rivera, E. X. R., Mathematical Model for Surgical Site Infection (SSI) Prediction in Patients Underwent to Ventral Hernia Repair (VHR). A Prospective Study in a Level I Hospital in Bogota, Colombia, Journal of the American College of Surgeons October 2020;231 (4 Supplement 2)():e132, 2020 October.  <https://dx.doi.org/10.1016/j.jamcollsurg.2020.08.345> | Conference abstract |
| 348. | Özbilgin Ş; Hancı V; Ömür D; Özbilgin M; Tosun M; Yurtlu S; Küçükgüçlü S; Arkan A, Morbidity and mortality predictivity of nutritional assessment tools in the postoperative care unit. Medicine (Baltimore) Oct 2016;95(40):e5038, 2016 Oct.  <https://dx.doi.org/10.1097/MD.0000000000005038> | Timepoint infection not in prediction window |
| 349. | Ozmen I; Grupa VEM; Bedrikovetski S; Dudi-Venkata NN; Huisman DE; Reudink M; Slooter GD; Sammour T; Kroon HM; Daams F, Risk Nomogram Does Not Predict Anastomotic Leakage After Colon Surgery Accurately: Results of the Multi-center LekCheck Study. J Gastrointest Surg Apr 2022;26(4):900-910, United States 2022 Apr.  <https://dx.doi.org/10.1007/s11605-021-05119-6> | Timepoint infection not specified |
| 350. | Paling FP; Olsen K; Ohneberg K; Wolkewitz M; Fowler VG Jr; DiNubile MJ; Jafri HS; Sifakis F; Bonten MJM; Harbarth SJ; Kluytmans JAJW, Risk prediction for Staphylococcus aureus surgical site infection following cardiothoracic surgery; A secondary analysis of the V710-P003 trial. PLoS One 2018;13(3):e0193445, 2018.  <https://dx.doi.org/10.1371/journal.pone.0193445> | Timepoint infection not in prediction window |
| 351. | Paolo Cerea, P.; Maranta, F.; Fossati, L.; Cianfanelli, L.; Pagnesi, M.; Cartella, I.; Pistoni, A.; Negri, S.; Cianflone, D., Development of a prognostic algorithm to predict functional recovery and complications following cardiac surgery, European Journal of Preventive Cardiology June 2018;25 (2 Supplement 1)():S45, 2018 June. | Conference abstract |
| 352. | Park, C. H.; Park, S. W.; Yang, M. J.; Moon, S. H.; Park, D. H., Pre- and post-procedure risk prediction models for post-endoscopic retrograde cholangiopancreatography pancreatitis, Surgical Endoscopy March 2022;36(3)():2052-2061, 2022 March.  <https://dx.doi.org/10.1007/s00464-021-08491-1> | Wrong intervention |
| 353. | Park, H. J.; Jung, D. Y.; Ji, W.; Choi, C. M., Detection of Bacteremia in Surgical In-Patients Using Recurrent Neural Network Based on Time Series Records: Development and Validation Study, J Med Internet Res Aug 4 2020;22(8):e19512, 2020 Aug 4.  <https://dx.doi.org/10.2196/19512> | Timepoint infection not specified |
| 354. | Paryavi E; Stall A; Gupta R; Scharfstein DO; Castillo RC; Zadnik M; Hui E; O'Toole RV, Predictive model for surgical site infection risk after surgery for high-energy lower-extremity fractures: development of the risk of infection in orthopedic trauma surgery score. J Trauma Acute Care Surg Jun 2013;74(6):1521-7, United States 2013 Jun.  <https://dx.doi.org/10.1097/TA.0b013e318292158d> | Timepoint infection not in prediction window |
| 355. | Pasic F; Salkic NN, Predictive score for anastomotic leakage after elective colorectal cancer surgery: a decision making tool for choice of protective measures. Surg Endosc Oct 2013;27(10):3877-82, Germany 2013 Oct.  <https://dx.doi.org/10.1007/s00464-013-2997-1> | Timepoint infection not specified |
| 356. | Passias PG; Oh C; Horn SR; Kim HJ; Hamilton DK; Sciubba DM; Neuman BJ; Buckland AJ; Poorman GW; Segreto FA; Bortz CA; Brown AE; Protopsaltis TS; Klineberg EO; Ames C; Smith JS; Lafage V, Predicting the occurrence of complications following corrective cervical deformity surgery: Analysis of a prospective multicenter database using predictive analytics. J Clin Neurosci Jan 2019;59():155-161, Scotland 2019 Jan.  <https://dx.doi.org/10.1016/j.jocn.2018.10.111> | Wrong outcomes |
| 357. | Pastor C; Baek JH; Varma MG; Kim E; Indorf LA; Garcia-Aguilar J, Validation of the risk index category as a predictor of surgical site infection in elective colorectal surgery. Dis Colon Rectum May 2010;53(5):721-7, United States 2010 May.  <https://dx.doi.org/10.1007/DCR.0b013e3181cc573b> | Timepoint infection not in prediction window |
| 358. | Paul M; Raz A; Leibovici L; Madar H; Holinger R; Rubinovitch B, Sternal wound infection after coronary artery bypass graft surgery: validation of existing risk scores. J Thorac Cardiovasc Surg Feb 2007;133(2):397-403, United States 2007 Feb.  <https://dx.doi.org/10.1016/j.jtcvs.2006.10.012> | Timepoint infection not in prediction window |
| 359. | Peng X; Hao X; Zhu T, A nomogram to predict postoperative infection for older hip fracture patients. Arch Orthop Trauma Surg Feb 2023;143(2):847-855, Germany 2023 Feb.  <https://dx.doi.org/10.1007/s00402-021-04171-w> | Timepoint infection not specified |
| 360. | Pergialiotis V; Papalios T; Haidopoulos D; Papapanagiotou A; Vlachos A; Rodolakis A; Thomakos N, Pre-Operative Neutrophil-to-Lymphocyte Ratio as a Predictor of Post-Operative Infectious Morbidity in Gynecologic Oncology Patients. Surg Infect (Larchmt) May 2023;24(4):390-396. United States 2023 May.  <https://dx.doi.org/10.1089/sur.2022.381> | Does not report (internal) validation performance metric(s) |
| 361. | Petrosyan, Y.; Thavorn, K.; Smith, G.; Maclure, M.; Preston, R.; van Walravan, C.; Forster, A. J., Predicting postoperative surgical site infection with administrative data: a random forests algorithm, BMC Med Res Methodol Aug 28 2021;21(1):179, 2021 Aug 28.  <https://dx.doi.org/10.1186/s12874-021-01369-9> | Wrong outcomes |
| 362. | Pierce, K. E.; Kapadia, B. H.; Naessig, S.; Ahmad, W.; Vira, S.; Paulino, C.; Gerling, M.; Passias, P. G., Validation of the ACS-NSQIP Risk Calculator: A Machine-Learning Risk Tool for Predicting Complications and Mortality Following Adult Spinal Deformity Corrective Surgery, Int J Spine Surg Dec 2021;15(6):1210-1216,  2021 Dec.  <https://dx.doi.org/10.14444/8153> | Does not report (internal) validation performance metric(s) |
| 363. | Pierce, K. E.; Passias, P. G.; Lafage, V.; Lafage, R.; Mundis, G. M.; Uribe, J. S.; Kim, H. J.; Protopsaltis, T. S.; Daniels, A. H.; Hart, R. A.; Burton, D. C.; Shaffrey, C. I.; Schwab, F. J.; Ames, C. P.; Smith, J. S.; Bess, S.; Klineberg, E. O.; Group, I. S. S., Validation of the ACS-NSQIP risk index in a prospective, multicenter adult spinal deformity database, Spine Journal September 2020;20 (9 Supplement)():S83,  2020 September.  <https://dx.doi.org/10.1016/j.spinee.2020.05.578> | Conference abstract |
| 364. | Pilz, G.; Kääb, S.; Kreuzer, E.; Werdan, K., Evaluation of definitions and parameters for sepsis assessment in patients after cardiac surgery, Infection Jan-Feb 1994;22(1):8-17, 1994 Jan-Feb.  <https://dx.doi.org/10.1007/bf01780757> | Timepoint infection not specified |
| 365. | Plat VD; Voeten DM; Daams F; van der Peet DL; Straatman J, C-reactive protein after major abdominal surgery in daily practice. Surgery Oct 2021;170(4):1131-1139, United States 2021 Oct.  <https://dx.doi.org/10.1016/j.surg.2021.04.025> | Wrong study design |
| 366. | Platt JJ; Ramanathan ML; Crosbie RA; Anderson JH; McKee RF; Horgan PG; McMillan DC, C-reactive protein as a predictor of postoperative infective complications after curative resection in patients with colorectal cancer. Ann Surg Oncol Dec 2012;19(13):4168-77, United States 2012 Dec.  <https://dx.doi.org/10.1245/s10434-012-2498-9> | Timepoint infection not specified |
| 367. | Pochhammer J; Scholtes B; Keuler J; Müssle B; Welsch T; Schäffer M, Serum C-reactive protein level after ventral hernia repair with mesh reinforcement can predict infectious complications: a retrospective cohort study. Hernia Feb 2020;24(1):41-48, France 2020 Feb.  <https://dx.doi.org/10.1007/s10029-018-1844-7> | Timepoint infection not specified |
| 368. | Prospero, E.; Barbadoro, P.; Annino, I.; D'Errico, M. M., Surgical site infections: might a longer locally defined T time affect the benchmarking? Am J Infect Control Nov 2007;35(9):582-4, 2007 Nov.  <https://dx.doi.org/10.1016/j.ajic.2007.01.004> | Timepoint infection not in prediction window |
| 369. | Putwatana, P.; Reodecha, P.; Sirapo-Ngam, Y.; Lertsithichai, P.; Sumboonnanonda, K., Nutrition screening tools and the prediction of postoperative infectious and wound complications: Comparison of methods in presence of risk adjustment, Nutrition June 2005;21(6):691-697, 2005 June.  <https://dx.doi.org/10.1016/j.nut.2004.10.015> | Wrong outcomes |
| 370. | Qu S; Sun M; Sun H; Hu B, C-reactive protein to albumin ratio (CAR) in predicting surgical site infection (SSI) following instrumented posterior lumbar interbody fusion (PLIF). Int Wound J Jan 2023;20(1):92-99, England 2023 Jan.  <https://dx.doi.org/10.1111/iwj.13843> | Publication date |
| 371. | Rafaqat W; Fatima HS; Kumar A; Khan S; Khurram M, Machine Learning Model for Assessment of Risk Factors and Postoperative Day for Superficial vs Deep/Organ-Space Surgical Site Infections. Surg Innov Apr 2023;():15533506231170933, United States 2023 Apr.  <https://dx.doi.org/10.1177/15533506231170933> | Wrong outcomes |
| 372. | Raja SG; Rochon M; Jarman JWE, Brompton Harefield Infection Score (BHIS): development and validation of a stratification tool for predicting risk of surgical site infection after coronary artery bypass grafting. Int J Surg Apr 2015;16(Pt A):69-73, England 2015 Apr.  <https://dx.doi.org/10.1016/j.ijsu.2015.02.008> | Timepoint infection not in prediction window |
| 373. | Ramanathan ML; MacKay G; Platt J; Horgan PG; McMillan DC, The impact of open versus laparoscopic resection for colon cancer on C-reactive protein concentrations as a predictor of postoperative infective complications. Ann Surg Oncol Mar 2015;22(3):938-43,  United States 2015 Mar.  <https://dx.doi.org/10.1245/s10434-014-4065-z> | Timepoint infection not specified |
| 374. | Ray RI; Aitken SA; McQueen MM; Court-Brown CM; Ralston SH, Predictors of poor clinical outcome following hip fracture in middle aged-patients. Injury Apr 2015;46(4):709-12, Netherlands 2015 Apr.  <https://dx.doi.org/10.1016/j.injury.2014.11.005> | Wrong outcomes |
| 375. | Reese SM; Knepper B; Young HL; Mauffrey C, Development of a surgical site infection prediction model in orthopaedic trauma: The Denver Health Model. Injury Dec 2017;48(12):2699-2704, Netherlands 2017 Dec.  <https://dx.doi.org/10.1016/j.injury.2017.10.011> | Timepoint infection not in prediction window |
| 376. | Reinersman, J. M.; Ferguson, M.; Allen, M. S.; Deschamps, C.; Nichols, F. C.; Shen, R.; Wigle, D. A.; Cassivi, S. D., External validation of the ferguson pulmonary risk score for predicting major pulmonary complications after oesophagectomy, Interactive Cardiovascular and Thoracic Surgery October 2014;19():S71-S72, 2014 October.  <https://dx.doi.org/10.1093/icvts/ivu276.240> | Conference abstract |
| 377. | Reisinger KW; Poeze M; Hulsewé KW; van Acker BA; van Bijnen AA; Hoofwijk AG; Stoot JH; Derikx JP, Accurate prediction of anastomotic leakage after colorectal surgery using plasma markers for intestinal damage and inflammation. J Am Coll Surg Oct 2014;219(4):744-51, United States 2014 Oct. <https://dx.doi.org/10.1016/j.jamcollsurg.2014.06.011> | Timepoint infection not specified |
| 378. | Ricco, J. B.; Guetarni, F.; Gargiulo, M., "Big Data" in Vascular Surgery: A Reality not to be Missed But to Use With Caution, European Journal of Vascular and Endovascular Surgery December 2019;58 (6 Supplement 3)():e624-e625, 2019 December.  <https://dx.doi.org/10.1016/j.ejvs.2019.09.110> | Conference abstract |
| 379. | Rivard, C. L.; Slagle, E. A.; Nahum, R.; Vogel, R. I.; Teoh, D., Evaluation of the NSQIP surgical risk calculator to predict complications in gynecologic oncology patients undergoing laparotomy, Journal of the American College of Surgeons October 2015;221(4):S98, 2015 October. | Conference abstract |
| 380. | Rivard, C.; Slagle, E.; Nahum, R.; Isaksson Vogel, R.; Teoh, D. G. K., Evaluation of the NSQIP surgical risk calculator to predict complications in gynecologic oncology patients undergoing laparotomy, Gynecologic Oncology April 2015;137():56-57, 2015 April.  <https://dx.doi.org/10.1016/j.ygyno.2015.01.132> | Duplicate (exact similar publication in other journal) |
| 381. | Roche M; Law TY; Kurowicki J; Sodhi N; Rosas S; Elson L; Summers S; Sabeh K; Mont MA, Albumin, Prealbumin, and Transferrin May Be Predictive of Wound Complications following Total Knee Arthroplasty. J Knee Surg Nov 2018;31(10):946-951, Germany 2018 Nov.  <https://dx.doi.org/10.1055/s-0038-1672122> | Does not report (internal) validation performance metric(s) |
| 382. | Rodriguez, R. A.; McClain, M.; Fahy, B. N.; Morris, K. T., Estimation of risk in cancer patients undergoing palliative procedures by the American College of Surgeons risk calculator, Journal of Clinical Oncology. Conference: Palliative Care in Oncology Symposium 2014;32(31 SUPPL. 1): 2014. | Conference abstract |
| 383. | Ros, E. P.; Benavides Buleje, J. A.; Marin, M. R.; Rodriguez, C. S.; Sanz, N. M.; Ruiz, M. V.; Cifuentes, A. S.; Parra Banos, P. A.; Marin-Blazquez, A. A., Can we predict and prevent post laparoscopic appendectomy intraabdominal abscess? Colorectal Disease September 2013;15():62, 2013 September.  <https://dx.doi.org/10.1111/codi.12353> | Conference abstract |
| 384. | Rosenthal, M.; Korenkevych, D.; Baslanti, T. O.; Webel, A.; Glerum, A.; Momcilovic, P.; Pardalos, P.; Bihorac, A., Algorithms can accurately predict risk for major postoperative complications using preoperative data, Critical Care Medicine December 2013;41(12):A173, 2013 December.  <https://dx.doi.org/10.1097/01.ccm.0000439941.83828.98> | Conference abstract |
| 385. | Rotem R; Erenberg M; Rottenstreich M; Segal D; Yohay Z; Idan I; Yohay D; Weintraub AY, Early prediction of post cesarean section infection using simple hematological biomarkers: A case control study. Eur J Obstet Gynecol Reprod Biol Feb 2020;245():84-88, Ireland 2020 Feb.  <https://dx.doi.org/10.1016/j.ejogrb.2019.12.009> | Timepoint infection not specified |
| 386. | Runner, R. P.; Bellamy, J. L.; Vu, C. C. L.; Erens, G. A.; Schenker, M. L.; Guild, G. N., 3^rd^, Modified Frailty Index Is an Effective Risk Assessment Tool in Primary Total Knee Arthroplasty, J Arthroplasty Sep 2017;32(9s):S177-s182, 2017 Sep.  <https://dx.doi.org/10.1016/j.arth.2017.03.046> | Does not report (internal) validation performance metric(s) |
| 387. | Rushton AB; Verra ML; Emms A; Heneghan NR; Falla D; Reddington M; Cole AA; Willems P; Benneker L; Selvey D; Hutton M; Heymans MW; Staal JB, Development and validation of two clinical prediction models to inform clinical decision-making for lumbar spinal fusion surgery for degenerative disorders and rehabilitation following surgery: protocol for a prospective observational study. BMJ Open May 2018;8(5):e021078, 2018 May.  <https://dx.doi.org/10.1136/bmjopen-2017-021078> | Wrong study design |
| 388. | Russotto V; Sabaté S; Canet J, Development of a prediction model for postoperative pneumonia: A multicentre prospective observational study. Eur J Anaesthesiol Feb 2019;36(2):93-104, England 2019 Feb.  <https://dx.doi.org/10.1097/EJA.0000000000000921> | Does not report (internal) validation performance metric(s) |
| 389. | Sá MPBO; Ferraz PE; Soares AF; Miranda RGA; Araújo ML; Silva FV; Lima RC, Development and Validation of a Stratification Tool for Predicting Risk of Deep Sternal Wound Infection after Coronary Artery Bypass Grafting at a Brazilian Hospital. Braz J Cardiovasc Surg Jan-Feb 2017;32(1):1-7, 2017 Jan-Feb.  <https://dx.doi.org/10.21470/1678-9741-2016-0030> | Timepoint infection not specified |
| 390. | Saadeddin ZM; Borrebach JD; Hodges JC; Avgerinos ED; Singh M; Siracuse JJ; Makaroun M; Eslami MH, Novel bypass risk predictive tool is superior to the 5-Factor Modified Frailty Index in predicting postoperative outcomes, J Vasc Surg Oct 2020;72(4):1427-1435.e1, United States 2020 Oct.  <https://dx.doi.org/10.1016/j.jvs.2019.11.050> | Wrong outcomes |
| 391. | Sabaté S; Mazo V; Canet J, Predicting postoperative pulmonary complications: implications for outcomes and costs. Curr Opin Anaesthesiol Apr 2014;27(2):201-9, United States 2014 Apr.  <https://dx.doi.org/10.1097/ACO.0000000000000045> | Wrong outcomes |
| 392. | Sadot, E.; Nash, G.; Smith, J. J.; Guillem, J. G.; Paty, P. B.; Temple, L. K.; Garcia-Aguilar, J.; Weiser, M., Hypophosphatemia as a novel early predictor of intra-abdominal infections in 7,423 patients undergoing colorectal surgery, Annals of Surgical Oncology February 2016;23(1):S36-S37, 2016 February.  <https://dx.doi.org/10.1245/s10434-015-5010-5> | Conference abstract |
| 393. | Sadot, E.; Roach, L.; McIntyre, C. A.; Allen, P. J.; Eaton, A. A.; D'Angelica, M. I.; DeMatteo, R. P.; Kingham, T. P.; Fong, Y.; Jarnagin, W. R., Early post-operative hypophosphatemia as a novel predictor of anastomotic failure after pancreatic resection: A risk-prediction tool, Hpb March 2015;17():38, 2015 March.  <https://dx.doi.org/10.1111/hpb.12399_8/abstract> | Conference abstract |
| 394. | Saeed K; Dale AP; Leung E; Cusack T; Mohamed F; Lockyer G; Arnaudov S; Wade A; Moran B; Lewis G; Dryden M; Cecil T; Cepeda JA, Procalcitonin levels predict infectious complications and response to treatment in patients undergoing cytoreductive surgery for peritoneal malignancy. Eur J Surg Oncol Feb 2016;42(2):234-43, England 2016 Feb.  <https://dx.doi.org/10.1016/j.ejso.2015.10.004> | Timepoint infection not in prediction window |
| 395. | Saji H; Ueno T; Nakamura H; Okumura N; Tsuchida M; Sonobe M; Miyazaki T; Aokage K; Nakao M; Haruki T; Ito H; Kataoka K; Okabe K; Tomizawa K; Yoshimoto K; Horio H; Sugio K; Ode Y; Takao M; Okada M; Chida M, A proposal for a comprehensive risk scoring system for predicting postoperative complications in octogenarian patients with medically operable lung cancer: JACS1303. Eur J Cardiothorac Surg Apr 2018;53(4):835-841, Germany 2018 Apr.  <https://dx.doi.org/10.1093/ejcts/ezx415> | Wrong outcomes |
| 396. | Sakaguchi S; Takifuji K; Arita S; Yamaue H, Development of an early diagnostic system using fuzzy theory for postoperative infections in patients with gastric cancer. Dig Surg 2004;21(3):210-4, Switzerland 2004.  <https://dx.doi.org/10.1159/000079394> | Timepoint infection not specified |
| 397. | Salati M; Migliorelli L; Moccia S; Andolfi M; Roncon A; Guiducci GM; Xiumè F; Tiberi M; Frontoni E; Refai M, A Machine Learning Approach for Postoperative Outcome Prediction: Surgical Data Science Application in a Thoracic Surgery Setting. World J Surg May 2021;45(5):1585-1594, United States 2021 May.  <https://dx.doi.org/10.1007/s00268-020-05948-7> | Does not report (internal) validation performance metric(s) |
| 398. | Salgado W Jr; de Queiroz Cunha F; dos Santos JS; Barbosa Nonino-Borges C; Kumar Sankarankutty A; de Castro E Silva O Jr; Ceneviva R, Early identification of infectious complications in bariatric surgery by the determination of peritoneal and systemic cytokines. Obes Surg Jul 2009;19(7):867-72, United States 2009 Jul.  <https://dx.doi.org/10.1007/s11695-009-9851-5> | Does not report (internal) validation performance metric(s) |
| 399. | Samareh A; Chang X; Lober WB; Evans HL; Wang Z; Qian X; Huang S, Artificial Intelligence Methods for Surgical Site Infection: Impacts on Detection, Monitoring, and Decision Making. Surg Infect (Larchmt) Oct 2019;20(7):546-554, United States 2019 Oct.  <https://dx.doi.org/10.1089/sur.2019.150> | Wrong study design |
| 400. | Sammour T; Cohen L; Karunatillake AI; Lewis M; Lawrence MJ; Hunter A; Moore JW; Thomas ML, Validation of an online risk calculator for the prediction of anastomotic leak after colon cancer surgery and preliminary exploration of artificial intelligence-based analytics. Tech Coloproctol Nov 2017;21(11):869-877, Italy 2017 Nov.  <https://dx.doi.org/10.1007/s10151-017-1701-1> | Timepoint infection not in prediction window |
| 401. | Sammour, T.; Lewis, M.; Thomas, M. L.; Lawrence, M. J.; Hunter, A.; Moore, J. W., A simple web-based risk calculator (www.anastomoticleak.com) is superior to the surgeon's estimate of anastomotic leak after colon cancer resection, Tech Coloproctol Jan 2017;21(1):35-41, 2017 Jan.  <https://dx.doi.org/10.1007/s10151-016-1567-7> | Timepoint infection not in prediction window |
| 402. | Sanaee MS; Pan K; Lee T; Koenig NA; Geoffrion R, Urinary tract infection after clean-contaminated pelvic surgery: a retrospective cohort study and prediction model. Int Urogynecol J Sep 2020;31(9):1821-1828, England 2020 Sep.  <https://dx.doi.org/10.1007/s00192-019-04119-0> | Timepoint infection not in prediction window |
| 403. | Sangsuwan, T.; Jamulitrat, S., Performance of the newly introduced NHSN logistic risk model for classification postcholecystectomy surgical site infection, Journal of the Medical Association of Thailand 2019;102(11):1205-1212, 2019. | Article not available |
| 404. | Sangsuwan, T.; Prueksasri, J.; Sae-Chan, J.; Chotpitchayanukul, K.; Jamulitrat, S., Performance of the newly introduced nhsn logistic risk model for classification postcholecystectomy surgical site infection, Antimicrobial Resistance and Infection Control. Conference: International Conference on Prevention and Infection Control, ICPIC 2017;6(Supplement 3): 2017.  <https://dx.doi.org/10.1186/s13756-017-0201-4> | Conference abstract |
| 405. | Santos M; Braga JU; Gomes RV; Werneck GL, Predictive factors for pneumonia onset after cardiac surgery in Rio de Janeiro, Brazil. Infect Control Hosp Epidemiol Apr 2007;28(4):382-8, United States 2007 Apr.  <https://dx.doi.org/10.1086/513119> | Conference abstract |
| 406. | Santos Mda S; Tura BR; Rouge A; Braga JU, External validation of models for predicting pneumonia after cardiac surgery. Surg Infect (Larchmt) Oct 2011;12(5):365-72, United States 2011 Oct.  <https://dx.doi.org/10.1089/sur.2010.014> | Timepoint infection not in prediction window |
| 407. | Sarmiento, E.; Carbone, J., Challenges associated with immunological scores for the prediction of the risk of infection after transplant, Transplant Infectious Disease 01 Feb 2015;17(1):156-157, 2015 01 Feb. | Wrong study design |
| 408. | Sasaki M; Miyoshi N; Fujino S; Ogino T; Takahashi H; Uemura M; Matsuda C; Yamamoto H; Mizushima T; Mori M; Doki Y, The Geriatric Nutritional Risk Index predicts postoperative complications and prognosis in elderly patients with colorectal cancer after curative surgery. Sci Rep Jul 2020;10(1):10744, 2020 Jul  <https://dx.doi.org/10.1038/s41598-020-67285-y> | Does not report (internal) validation performance metric(s) |
| 409. | Sato T; Kondo H; Watanabe A; Nakajima J; Niwa H; Horio H; Okami J; Okumura N; Sugio K; Teramukai S; Kishi K; Ebina M; Sugiyama Y; Kondo T; Date H, A simple risk scoring system for predicting acute exacerbation of interstitial pneumonia after pulmonary resection in lung cancer patients. Gen Thorac Cardiovasc Surg Mar 2015;63(3):164-72, Japan 2015 Mar.  <https://dx.doi.org/10.1007/s11748-014-0487-6> | Wrong outcomes |
| 410. | Saunders L; Perennec-Olivier M; Jarno P; L'Hériteau F; Venier AG; Simon L; Giard M; Thiolet JM; Viel JF, Improving prediction of surgical site infection risk with multilevel modeling. PLoS One 2014;9(5):e95295, 2014.  <https://dx.doi.org/10.1371/journal.pone.0095295> | Timepoint infection not in prediction window |
| 411. | Saze Z; Miyata H; Konno H; Gotoh M; Anazawa T; Tomotaki A; Wakabayashi G; Mori M, Risk Models of Operative Morbidities in 16,930 Critically Ill Surgical Patients Based on a Japanese Nationwide Database. Medicine (Baltimore) Jul 2015;94(30):e1224, 2015 Jul.  <https://dx.doi.org/10.1097/MD.0000000000001224> | Wrong patient population |
| 412. | Scattarelli, A.; Carriou, M.; Boulet, L.; Chati, R.; Coget, J.; Bridoux, V.; Tuech, J. J.; Roman, H., C-reactive protein assessment to predict early septic complications after laparoscopic bowel resection for endometriosis: a diagnostic study, Bjog Aug 2019;126(9):1176-1182, 2019 Aug.  <https://dx.doi.org/10.1111/1471-0528.15812> | Does not report (internal) validation performance metric(s) |
| 413. | Schlosser, K. A.; Kao, A. M.; Zhang, Y.; Prasad, T.; Kasten, K. R.; Davis, B. R.; Heniford, B. T.; Colavita, P. D., MELD-Na score associated with postoperative complications in hernia repair in non-cirrhotic patients, Hernia Feb 2019;23(1):51-59, 2019 Feb.  <https://dx.doi.org/10.1007/s10029-018-1849-2> | Wrong outcomes |
| 414. | Sekine Y; Suzuki H; Nakajima T; Yasufuku K; Yoshida S, Risk quantification for pulmonary complications after lung cancer surgery. Surg Today Nov 2010;40(11):1027-33, Japan 2010 Nov.  <https://dx.doi.org/10.1007/s00595-009-4182-7> | Wrong outcomes |
| 415. | Sevinyan, L.; Arakkal, D.; Prabhu, P.; Tailor, A.; Williams, P.; Madhuri, T. K., P-possum vs ACS-NSQIP: Patient understanding of post-operative risks and shared decision, International Journal of Gynecological Cancer December 2020;30 (SUPPL 4)():A135-A136, 2020 December.  <https://dx.doi.org/10.1136/ijgc-2020-ESGO.234> | Conference abstract |
| 416. | Shahian DM; O'Brien SM; Filardo G; Ferraris VA; Haan CK; Rich JB; Normand SL; DeLong ER; Shewan CM; Dokholyan RS; Peterson ED; Edwards FH; Anderson RP, The Society of Thoracic Surgeons 2008 cardiac surgery risk models: part 3--valve plus coronary artery bypass grafting surgery. Ann Thorac Surg Jul 2009;88(1 Suppl):S43-62, Netherlands 2009 Jul.  <https://dx.doi.org/10.1016/j.athoracsur.2009.05.055> | Timepoint infection not in prediction window |
| 417. | Shahrestani, S.; Bakhsheshian, J.; Solaru, S.; Ton, A.; Ballatori, A. M.; Chen, X. T.; Ariani, R.; Hsieh, P.; Buser, Z.; Wang, J. C., Inclusion of Frailty Improves Predictive Modeling for Postoperative Outcomes in Surgical Management of Primary and Secondary Lumbar Spine Tumors, World Neurosurg Sep 2021;153():e454-e463, 2021 Sep.  <https://dx.doi.org/10.1016/j.wneu.2021.06.143> | Does not report (internal) validation performance metric(s) |
| 418. | Shao CY; Liu KC; Li CL; Cong ZZ; Hu LW; Luo J; Diao YF; Xu Y; Ji SG; Qiang Y; Shen Y, C-reactive protein to albumin ratio is a key indicator in a predictive model for anastomosis leakage after esophagectomy: Application of classification and regression tree analysis. Thorac Cancer Apr 2019;10(4):728-737, 2019 Apr.  <https://dx.doi.org/10.1111/1759-7714.12990> | Timepoint infection not specified |
| 419. | Shao, S.; Liu, L.; Zhao, Y.; Mu, L.; Lu, Q.; Qin, J., Application of machine learning for predicting anastomotic leakage in patients with gastric adenocarcinoma who received total or proximal gastrectomy, Journal of Personalized Medicine August 2021;11(8) (no pagination)(): 2021 August.  <https://dx.doi.org/10.3390/jpm11080748> | Does not report (internal) validation performance metric(s) |
| 420. | Shao S; Zhao Y; Lu Q; Liu L; Mu L; Qin J, Artificial intelligence assists surgeons' decision-making of temporary ileostomy in patients with rectal cancer who have received anterior resection. Eur J Surg Oncol Feb 2023;49(2):433-439, England 2023 Feb.  <https://dx.doi.org/10.1016/j.ejso.2022.09.020> | Timepoint infection not specified |
| 421. | Sharma P; Patel K; Baria K; Lakhia K; Malhotra A; Shah K; Patel S, Procalcitonin level for prediction of postoperative infection in cardiac surgery. Asian Cardiovasc Thorac Ann May 2016;24(4):344-9, England 2016 May.  <https://dx.doi.org/10.1177/0218492316640953> | Wrong study design |
| 422. | Sharouf F; Hussain RN; Hettipathirannahelage S; Martin J; Gray W; Zaben M, C-reactive protein kinetics post elective cranial surgery. A prospective observational study. Br J Neurosurg Feb 2020;34(1):46-50, England 2020 Feb.  <https://dx.doi.org/10.1080/02688697.2019.1680795> | Timepoint infection not specified |
| 423. | Shavit L; Dolgoker I; Ivgi H; Assous M; Slotki I, Neutrophil gelatinase-associated lipocalin as a predictor of complications and mortality in patients undergoing non-cardiac major surgery. Kidney Blood Press Res 2011;34(2):116-24, Switzerland 2011.  <https://dx.doi.org/10.1159/000323897> | Does not report (internal) validation performance metric(s) |
| 424. | Shelton, J. A.; Brown, J. J.; Young, J. A., Preoperative C-reactive protein predicts the severity and likelihood of complications following appendicectomy, Ann R Coll Surg Engl Jul 2014;96(5):369-72, 2014 Jul.  <https://dx.doi.org/10.1308/003588414x13946184901722> | Does not report (internal) validation performance metric(s) |
| 425. | Shen, R.; Zhang, W.; Ming, S.; Li, L.; Peng, Y.; Gao, X., Gender-related differences in the performance of sequential organ failure assessment (SOFA) to predict septic shock after percutaneous nephrolithotomy, Urolithiasis Feb 2021;49(1):65-72, 2021 Feb.  <https://dx.doi.org/10.1007/s00240-020-01190-x> | Timepoint infection not specified |
| 426. | Shen Z; Lin Y; Ye Y; Jiang K; Xie Q; Gao Z; Wang S, The development and validation of a novel model for predicting surgical complications in colorectal cancer of elderly patients: Results from 1008 cases. Eur J Surg Oncol Apr 2018;44(4):490-495, England 2018 Apr  <https://dx.doi.org/10.1016/j.ejso.2018.01.007> | Wrong outcomes |
| 427. | Shi, J.; Wu, Z.; Wu, X.; Shan, F.; Zhang, Y.; Ying, X.; Li, Z.; Ji, J., Early Diagnosis of Anastomotic Leakage After Gastric Cancer Surgery Via Analysis of Inflammatory Factors in Abdominal Drainage, Ann Surg Oncol Feb 2022;29(2):1230-1241, 2022 Feb.  <https://dx.doi.org/10.1245/s10434-021-10763-y> | Does not report (internal) validation performance metric(s) |
| 428. | Shimizu T; Ishizuka M; Kubota K, The preoperative serum C-reactive protein level is a useful predictor of surgical site infections in patients undergoing appendectomy. Surg Today Nov 2015;45(11):1404-10, Japan 2015 Nov.  <https://dx.doi.org/10.1007/s00595-014-1086-y> | Wrong outcomes |
| 429. | Shiwakoti, E.; Song, J.; Li, J.; Wu, S.; Zhang, Z., Prediction model for anastomotic leakage after laparoscopic rectal cancer resection, Journal of International Medical Research 2020;48(9): 2020.  <https://dx.doi.org/10.1177/0300060520957547> | Does not report (internal) validation performance metric(s) |
| 430. | Sisti G; Sabre A; Mercado F; Upadhyay R; Joseph J; Schiattarella A, Predictive value of preoperative complete blood count components on the occurrence of surgical site infection after elective cesarean section. Minerva Obstet Gynecol Apr 2023;75(2):126-131, Italy 2023 Apr.  <https://dx.doi.org/10.23736/S2724-606X.21.04951-4> | Article not available |
| 431. | Sofo, L.; Caprino, P.; Potenza, A. E.; Sacchetti, F.; Schena, C. A., Machine learning (ML) in RCU-operated patients: Can we predict postoperative complications? Journal of Crohn's and Colitis February 2018;12 (Supplement 1)():S455, 2018 February. | Conference abstract |
| 432. | Sofo L; Caprino P; Schena CA; Sacchetti F; Potenza AE; Ciociola A, New perspectives in the prediction of postoperative complications for high-risk ulcerative colitis patients: machine learning preliminary approach. Eur Rev Med Pharmacol Sci Dec 2020;24(24):12781-12787, Italy 2020 Dec.  <https://dx.doi.org/10.26355/eurrev_202012_24178> | Timepoint infection not specified |
| 433. | Soguero-Ruiz C; Fei WM; Jenssen R; Augestad KM; Álvarez JL; Jiménez IM; Lindsetmo RO; Skrøvseth SO, Data-driven Temporal Prediction of Surgical Site Infection. AMIA Annu Symp Proc 2015;2015():1164-73, 2015. | Timepoint infection not specified |
| 434. | Soguero-Ruiz, C.; Hindberg, K.; Mora-Jiménez, I.; Rojo-Álvarez, J. L.; Skrøvseth, S. O.; Godtliebsen, F.; Mortensen, K.; Revhaug, A.; Lindsetmo, R. O.; Augestad, K. M.; Jenssen, R., Predicting colorectal surgical complications using heterogeneous clinical data and kernel methods, J Biomed Inform Jun 2016;61():87-96, 2016 Jun.  <https://dx.doi.org/10.1016/j.jbi.2016.03.008> | Duplicate (exact similar publication in other journal) |
| 435. | Soguero-Ruiz C; Hindberg K; Rojo-Alvarez JL; Skrovseth SO; Godtliebsen F; Mortensen K; Revhaug A; Lindsetmo RO; Augestad KM; Jenssen R, Support Vector Feature Selection for Early Detection of Anastomosis Leakage From Bag-of-Words in Electronic Health Records. IEEE J Biomed Health Inform Sep 2016;20(5):1404-15, United States 2016 Sep.  <https://dx.doi.org/10.1109/JBHI.2014.2361688> | Timepoint infection not specified |
| 436. | Sohn, S.; Larson, D. W.; Habermann, E. B.; Naessens, J. M.; Alabbad, J. Y.; Liu, H. Detection of clinically important colorectal surgical site infection using Bayesian network, J Surg Res Mar 2017;209():168-173, 2017 Mar.  <https://dx.doi.org/10.1016/j.jss.2016.09.058> | Wrong outcomes |
| 437. | Son HJ; Roh JL; Choi SH; Nam SY; Kim SY, Nutritional and hematologic markers as predictors of risk of surgical site infection in patients with head and neck cancer undergoing major oncologic surgery. Head Neck Mar 2018;40(3):596-604, United States 2018 Mar.  <https://dx.doi.org/10.1002/hed.25031> | Does not report (internal) validation performance metric(s) |
| 438. | Song, Y.; Liu, J.; Lei, M.; Wang, Y.; Fu, Q.; Wang, B.; Guo, Y.; Mi, W.; Tong, L., An External-Validated Algorithm to Predict Postoperative Pneumonia Among Elderly Patients With Lung Cancer After Video-Assisted Thoracoscopic Surgery, Front Oncol 2021;11():777564, 2021.  <https://dx.doi.org/10.3389/fonc.2021.777564> | Timepoint infection not specified |
| 439. | Sood, A.; Abdollah, F.; Sammon, J.; Klett, D.; Pucheril, D.; Majumder, K.; Schmid, M.; Kibel, A.; Menon, M.; Trinh, Q. D., Postoperative sepsis prediction in patients undergoing major cancer surgery, Journal of Urology April 2015;193(4):e107, 2015 April. | Conference abstract |
| 440. | Sood A; Abdollah F; Sammon JD; Arora N; Weeks M; Peabody JO; Menon M; Trinh QD, Postoperative sepsis prediction in patients undergoing major cancer surgery. J Surg Res Mar 2017;209():60-69, United States 2017 Mar.  <https://dx.doi.org/10.1016/j.jss.2016.09.059> | Timepoint infection not in prediction window |
| 441. | Souwer ETD; Bastiaannet E; Steyerberg EW; Dekker JT; van den Bos F; Portielje JEA, Risk prediction models for postoperative outcomes of colorectal cancer surgery in the older population - a systematic review. J Geriatr Oncol Nov 2020;11(8):1217-1228, Netherlands 2020 Nov.  <https://dx.doi.org/10.1016/j.jgo.2020.04.006> | Wrong study design |
| 442. | Souza, F.; Couto, B.; da Conceicao, F. L. A.; da Silva, G. H. S.; Dias, I. G.; Rigueira, R. V. M.; Pimenta, G. M.; Martins, M.; Mendes, J. C.; Fagundes, A. M.; Escalda, B. V. F.; de Souza, I. M.; de Vasconcelos, L. F.; Medeiros, M. E. R.; de Almeida, T. A., Pattern Recognition Algorithms for Predicting Surgical Site Infection in Abdominal Hysterectomy, Infection Control and Hospital Epidemiology Oct 2020;41():S344-S345, 2020 Oct.  <https://dx.doi.org/10.1017/ice.2020.958> | Conference abstract |
| 443. | Souza, F. H. B. D.; Couto, B. R. G. M.; Braga, G. M.; Teixeira, J. A.; Santos, R. C.; Martins, J. M. C.; Sousa, K. S. D.; De Souza, D. N.; Alves, G. B., Medicine allied to technology: The use of artificial neural networks in the prediction of surgical site infection for general surgery service, Antimicrobial Resistance and Infection Control. Conference: 5th International Conference on Prevention and Infection Control, ICPIC 2019;8(Supplement 1): 2019.  <https://dx.doi.org/10.1186/s13756-019-0567-6> | Conference abstract |
| 444. | Souza, F. H. B. D.; Couto, B. R. G. M.; De Mello, F. B.; Caldeira, I.; De Morais, J. S. F. R.; Mota, L. G.; Dumont, M. F.; Savio, R. W.; Mazzoni, S. M., Algorithms for pattern recognition for prediction of surgical site infection in vascular surgeries, Antimicrobial Resistance and Infection Control. Conference: 5th International Conference on Prevention and Infection Control, ICPIC 2019;8(Supplement 1): 2019.  <https://dx.doi.org/10.1186/s13756-019-0567-6> | Conference abstract |
| 445. | Souza, F. H. B. D.; Couto, B. R. G. M.; Maroca, A. M. R.; Soares, I. H. S.; Duarte, J. M. D. C.; Costa, S. C., Power of prediction of surgical site infection in caesarean surgery using pattern-based analysis based on multilayer perceptron artificial neural networks, Antimicrobial Resistance and Infection Control. Conference: 5th International Conference on Prevention and Infection Control, ICPIC 2019;8(Supplement 1): 2019.  <https://dx.doi.org/10.1186/s13756-019-0567-6> | Conference abstract |
| 446. | Souza, F. H. B. D.; Couto, B. R. G. M.; Matias, J. D. O.; De Araujo, L. L.; Rossati, L. S.; Polidoro, L. R., Risk prediction for surgical site infection in craniotomy patients, Antimicrobial Resistance and Infection Control. Conference: 5th International Conference on Prevention and Infection Control, ICPIC 2019;8(Supplement 1): 2019.  <https://dx.doi.org/10.1186/s13756-019-0567-6> | Conference abstract |
| 447. | Souza, F. H. B. D.; Couto, B. R. G. M.; Michelini, A. C. L.; De Melo, A. P. R.; Guerra, L. G.; Costa, L. M. B.; Melo, M. V.; De Oliveira Junior, O. J., Risk of surgical site infection after cardiac surgeries: Pattern-based analysis based on multilayer perceptron artificial neural networks, Antimicrobial Resistance and Infection Control. Conference: 5th International Conference on Prevention and Infection Control, ICPIC 2019;8(Supplement 1): 2019.  <https://dx.doi.org/10.1186/s13756-019-0567-6> | Conference abstract |
| 448. | Souza, F. H. B. D.; Couto, B. R. G. M.; Serpa, G. S. D. M.; Abelha, I. P. D. A. L.; Valadao, L. F.; Bernardes, M. E. C.; Vidigal, M. C. B.; Haddad, Y. D. A. E. S., Using artificial neural networks to predict of surgical site infection in orthopedic patients, Antimicrobial Resistance and Infection Control. Conference: 5th International Conference on Prevention and Infection Control, ICPIC 2019;8(Supplement 1): 2019.  <https://dx.doi.org/10.1186/s13756-019-0567-6> | Conference abstract |
| 449. | Sparreboom, C. L.; Komen, N.; Rizopoulos, D.; Verhaar, A. P.; Dik, W. A.; Wu, Z.; van Westreenen, H. L.; Doornebosch, P. G.; Dekker, J. W. T.; Menon, A. G.; Daams, F.; Lips, D.; van Grevenstein, W. M. U.; Karsten, T. M.; Bayon, Y.; Peppelenbosch, M. P.; Wolthuis, A. M.; D'Hoore, A.; Lange, J. F., A multicentre cohort study of serum and peritoneal biomarkers to predict anastomotic leakage after rectal cancer resection, Colorectal Disease. 2019;(): 2019.  <https://dx.doi.org/10.1111/codi.14789> | Timepoint infection not specified |
| 450. | Stepanov, I. A.; Beloborodov, V. A.; Shameeva, M. A.; Borisov, E. B., A scoring system to predict the risk of surgical site infections after spinal surgery, Coluna/ Columna 2021;20(3):212-216, 2021.  <https://dx.doi.org/10.1590/S1808-185120212003251045> | Wrong outcomes |
| 451. | Straatman, J.; Harmsen, A. M. K.; Cuesta, M. A.; Berkhof, J.; Jansma, E. P.; van der Peet, D. L., Predictive Value of C-Reactive Protein for Major Complications after Major Abdominal Surgery: A Systematic Review and Pooled-Analysis, PLoS One Jul 2015;10(7):14, 2015 Jul.  <https://dx.doi.org/10.1371/journal.pone.0132995> | Wrong outcomes |
| 452. | Strabelli TM; Stolf NA; Uip DE, Practical use of a risk assessment model for complications after cardiac surgery. Arq Bras Cardiol Nov 2008;91(5):342-7, Brazil 2008 Nov.  <https://dx.doi.org/10.1590/s0066-782x2008001700010> | Timepoint infection not specified |
| 453. | Strobel RJ; Liang Q; Zhang M; Wu X; Rogers MA; Theurer PF; Fishstrom AB; Harrington SD; DeLucia A 3rd; Paone G; Patel HJ; Prager RL; Likosky DS, A Preoperative Risk Model for Postoperative Pneumonia After Coronary Artery Bypass Grafting. Ann Thorac Surg Oct 2016;102(4):1213-9, 2016 Oct.  <https://dx.doi.org/10.1016/j.athoracsur.2016.03.074> | Timepoint infection not specified |
| 454. | Suehiro K; Tanaka K; Matsuura T; Funao T; Yamada T; Mori T; Tsuchiya M; Nishikawa K, Preoperative hydroperoxide concentrations are associated with a risk of postoperative complications after cardiac surgery. Anaesth Intensive Care Jul 2014;42(4):487-94, United States 2014 Jul.  <https://dx.doi.org/10.1177/0310057X1404200410> | Wrong outcomes |
| 455. | Sugita S; Hozumi T; Yamakawa K; Goto T; Kondo T, White Blood Cell Count and C-Reactive Protein Variations After Posterior Surgery With Intraoperative Radiotherapy for Spinal Metastasis., Clin Spine Surg Oct 2017;30(8):E1022-E1025, United States 2017 Oct.  <https://dx.doi.org/10.1097/BSD.0000000000000261> | Wrong outcomes |
| 456. | Sun, C.; Pei, L.; Zhang, Y.; Huang, Y., Risk prediction model for postoperative healthcare associated infection based on deep learning, Anesthesia and Analgesia September 2021;133(3 SUPPL 2)():1468-1469, 2021 September. | Conference abstract |
| 457. | Sun ZW; Du H; Li JR; Qin HY, Constructing a risk prediction model for anastomotic leakage after esophageal cancer resection. J Int Med Res Apr 2020;48(4):300060519896726, 2020 Apr.  <https://dx.doi.org/10.1177/0300060519896726> | Does not report (internal) validation performance metric(s) |
| 458. | Sutherland A; Thomas M; Brandon RA; Brandon RB; Lipman J; Tang B; McLean A; Pascoe R; Price G; Nguyen T; Stone G; Venter D, Development and validation of a novel molecular biomarker diagnostic test for the early detection of sepsis. Crit Care Jun 2011;15(3):R149, 2011 Jun.  <https://dx.doi.org/10.1186/cc10274> | Wrong outcomes |
| 459. | Sweerts L; Dekkers PW; van der Wees PJ; van Susante JLC; de Jong LD; Hoogeboom TJ; van de Groes SAW, External Validation of Prediction Models for Surgical Complications in People Considering Total Hip or Knee Arthroplasty Was Successful for Delirium but Not for Surgical Site Infection, Postoperative Bleeding, and Nerve Damage: A Retrospective Cohort Stud, J Pers Med Jan 2023;13(2): Switzerland 2023 Jan.  <https://dx.doi.org/10.3390/jpm13020277> | Timepoint infection not specified |
| 460. | Sweerts L; Hoogeboom TJ; van Wessel T; van der Wees PJ; van de Groes SAW, Development of prediction models for complications after primary total hip and knee arthroplasty: a single-centre retrospective cohort study in the Netherlands. BMJ Open Aug 2022;12(8):e062065, England 2022 Aug.  <https://dx.doi.org/10.1136/bmjopen-2022-062065> | Timepoint infection not specified |
| 461. | Taha-Mehlitz, S.; Wentzler, L.; Angehrn, F.; Hendie, A.; Ochs, V.; Staartjes, V. E.; von Flue, M.; Taha, A.; Steinemann, D., Machine-learning based preoperative analytics for the prediction of anastomotic insufficiency in colorectal surgery: A single-centre pilot study, medRxiv. 2021;13(): 2021.  <https://dx.doi.org/10.1101/2021.12.11.21267569> | Timepoint infection not specified |
| 462. | Takakura Y; Hinoi T; Egi H; Shimomura M; Adachi T; Saito Y; Tanimine N; Miguchi M; Ohdan H, Procalcitonin as a predictive marker for surgical site infection in elective colorectal cancer surgery. Langenbecks Arch Surg Aug 2013;398(6):833-9, Germany 2013 Aug.  <https://dx.doi.org/10.1007/s00423-013-1095-0> | Timepoint infection not specified |
| 463. | Talbot TR; D'Agata EM; Brinsko V; Lee B; Speroff T; Schaffner W, Perioperative blood transfusion is predictive of poststernotomy surgical site infection: marker for morbidity or true immunosuppressant? Clin Infect Dis May 2004;38(10):1378-82, United States 2004 May.  <https://dx.doi.org/10.1086/386334> | Does not report (internal) validation performance metric(s) |
| 464. | Tan, T. L.; Maltenfort, M. G.; Chen, A. F.; Shahi, A.; Higuera, C. A.; Siqueira, M.; Parvizi, J., Development and Evaluation of a Preoperative Risk Calculator for Periprosthetic Joint Infection Following Total Joint Arthroplasty, J Bone Joint Surg Am May 2 2018;100(9):777-785, 2018 May 2.  <https://dx.doi.org/10.2106/jbjs.16.01435> | Timepoint infection not in prediction window |
| 465. | Tan, T. P.; Arekapudi, A.; Metha, J.; Prasad, A.; Venkatraghavan, L., Neutrophil-lymphocyte ratio as predictor of mortality and morbidity in cardiovascular surgery: a systematic review, ANZ J Surg Jun 2015;85(6):414-9, 2015 Jun.  <https://dx.doi.org/10.1111/ans.13036> | Wrong outcomes |
| 466. | Tang, M. T.; Li, S.; Liu, X.; Huang, X.; Zhang, D. Y.; Lei, M. X., Early Detection of Pneumonia with the Help of Dementia in Geriatric Hip Fracture Patients, Orthop Surg Jan 2022;14(1):129-138, 2022 Jan.  <https://dx.doi.org/10.1111/os.13199> | Timepoint infection not specified |
| 467. | Tang, Y.; Zhang, C.; Mo, C.; Gui, C.; Luo, J.; Wu, R., Predictive Model for Systemic Infection After Percutaneous Nephrolithotomy and Related Factors Analysis, Front Surg 2021;8():696463, 2021. <https://dx.doi.org/10.3389/fsurg.2021.696463> | Does not report (internal) validation performance metric(s) |
| 468. | Tariq A; Lancaster L; Elugunti P; Siebeneck E; Noe K; Borah B; Moriarty J; Banerjee I; Patel BN, Graph convolutional network-based fusion model to predict risk of hospital acquired infections. J Am Med Inform Assoc May 2023;30(6):1056-1067, England 2023 May.  <https://dx.doi.org/10.1093/jamia/ocad045> | Timepoint infection not in prediction window |
| 469. | Tatsuoka T; Okuyama T; Takeshita E; Oi H; Noro T; Mitsui T; Yoshitomi H; Oya M, Early detection of infectious complications using C-reactive protein and the procalcitonin levels after laparoscopic colorectal resection: a prospective cohort study. Surg Today Mar 2021;51(3):397-403, Japan 2021 Mar.  <https://dx.doi.org/10.1007/s00595-020-02111-6> | Timepoint infection not specified |
| 470. | Thirukumaran CP; Zaman A; Rubery PT; Calabria C; Li Y; Ricciardi BF; Bakhsh WR; Kautz H, Natural Language Processing for the Identification of Surgical Site Infections in Orthopaedics. J Bone Joint Surg Am Dec 2019;101(24):2167-2174, 2019 Dec.  <https://dx.doi.org/10.2106/JBJS.19.00661> | Timepoint infection not in prediction window |
| 471. | Thottakkara P; Ozrazgat-Baslanti T; Hupf BB; Rashidi P; Pardalos P; Momcilovic P; Bihorac A, Application of Machine Learning Techniques to High-Dimensional Clinical Data to Forecast Postoperative Complications. PLoS One 2016;11(5):e0155705, 2016  <https://dx.doi.org/10.1371/journal.pone.0155705> | Timepoint infection not specified |
| 472. | Tian JY; Hao XY; Cao FY; Liu JJ; Li YX; Guo YX; Mi WD; Tong L; Fu Q, Preoperative Frailty Assessment Predicts Postoperative Mortality, Delirium and Pneumonia in Elderly Lung Cancer Patients: A Retrospective Cohort Study. Ann Surg Oncol Jun 2023;(): United States 2023 Jun.  <https://dx.doi.org/10.1245/s10434-023-13696-w> | Timepoint infection not specified |
| 473. | Tleyjeh, I.; Alasmari, F.; Riaz, M.; Greason, K.; Berbari, E.; Virk, A.; Baddour, L., A clinical prediction rule of deep sternal wound infections after coronary artery bypass graft surgery: A populationbased cohort study, 1993-2008, Clinical Microbiology and Infection April 2012;18():79, 2012 April.  <https://dx.doi.org/10.1111/j.1469-0691.2012.03801.x> | Conference abstract |
| 474. | Toumpoulis, I. K.; Anagnostopoulos, C. E.; Swistel, D. G.; DeRose, J. J., Jr., Does EuroSCORE predict length of stay and specific postoperative complications after cardiac surgery? Eur J Cardiothorac Surg Jan 2005;27(1):128-33, 2005 Jan.  <https://dx.doi.org/10.1016/j.ejcts.2004.09.020> | Timepoint infection not specified |
| 475. | Traven SA; Reeves RA; Althoff AD; Slone HS; Walton ZJ, New Five-Factor Modified Frailty Index Predicts Morbidity and Mortality in Geriatric Hip Fractures. J Orthop Trauma Jul 2019;33(7):319-323, United States 2019 Jul.  <https://dx.doi.org/10.1097/BOT.0000000000001455> | Wrong outcomes |
| 476. | Trivedi, A.; Ezomo, O. T.; Gronbeck, C.; Harrington, M. A.; Halawi, M. J., Time Trends and Risk Factors for 30-Day Adverse Events in Black Patients Undergoing Primary Total Knee Arthroplasty, J Arthroplasty Nov 2020;35(11):3145-3149, 2020 Nov.  <https://dx.doi.org/10.1016/j.arth.2020.06.013> | Wrong outcomes |
| 477. | Tseng, Y. J.; Huang, B. C.; Lin, H. C.; Chen, M. Y.; Shang, R. J.; Sheng, W. H.; Chen, Y. C.; Lai, F.; Chang, S. C., Development and evaluation of a hospital-wide healthcare associated surgical site infection detection algorithm, Journal of Microbiology, Immunology and Infection April 2015;48(2):S43, 2015 April | Conference abstract |
| 478. | Tsiouris, A.; Horst, H. M.; Paone, G.; Hodari, A.; Eichenhorn, M.; Rubinfeld, I., Preoperative risk stratification for thoracic surgery using the American College of Surgeons National Surgical Quality Improvement Program data set: functional status predicts morbidity and mortality, J Surg Res Sep 2012;177(1):1-6, 2012 Sep.  <https://dx.doi.org/10.1016/j.jss.2012.02.048> | Wrong outcomes |
| 479. | Tu RH; Huang CM; Lin JX; Chen QY; Zheng CH; Li P; Xie JW; Wang JB; Lu J; Cao LL; Lin M, A scoring system to predict the risk of organ/space surgical site infections after laparoscopic gastrectomy for gastric cancer based on a large-scale retrospective study. Surg Endosc Jul 2016;30(7):3026-34, 2016 Jul.  <https://dx.doi.org/10.1007/s00464-015-4594-y> | Does not report (internal) validation performance metric(s) |
| 480. | Tu RH; Lin JX; Zheng CH; Li P; Xie JW; Wang JB; Lu J; Chen QY; Cao LL; Lin M; Huang CM, Development of a nomogram for predicting the risk of anastomotic leakage after a gastrectomy for gastric cancer. Eur J Surg Oncol Feb 2017;43(2):485-492, England 2017 Feb.  <https://dx.doi.org/10.1016/j.ejso.2016.11.022> | Timepoint infection not specified |
| 481. | Tunthanathip T; Sae-Heng S; Oearsakul T; Sakarunchai I; Kaewborisutsakul A; Taweesomboonyat C, Machine learning applications for the prediction of surgical site infection in neurological operations. Neurosurg Focus Aug 2019;47(2):E7, United States 2019 Aug.  <https://dx.doi.org/10.3171/2019.5.FOCUS19241> | Timepoint infection not in prediction window |
| 482. | Turner, P. L.; Saager, L.; Dalton, J.; Abd-Elsayed, A.; Roberman, D.; Melara, P.; Kurz, A.; Turan, A., A nomogram for predicting surgical complications in bariatric surgery patients, Obes Surg May 2011;21(5):655-62, 2011 May.  <https://dx.doi.org/10.1007/s11695-010-0325-6> | Wrong outcomes |
| 483. | Valls V; Díez M; Ena J; Gutiérrez A; Gómez-Herruz P; Martín A; Gónzalez-Palacios R; Granell J, Evaluation of the SENIC risk index in a Spanish university hospital. Infect Control Hosp Epidemiol Mar 1999;20(3):196-9, United States 1999 Mar.  <https://dx.doi.org/10.1086/501611> | Wrong patient population |
| 484. | van Niftrik, C. H. B.; van der Wouden, F.; Staartjes, V. E.; Fierstra, J.; Stienen, M. N.; Akeret, K.; Sebok, M.; Fedele, T.; Sarnthein, J.; Bozinov, O.; Krayenbuhl, N.; Regli, L.; Serra, C., Machine Learning Algorithm Identifies Patients at High Risk for Early Complications After Intracranial Tumor Surgery: Registry-Based Cohort Study, Neurosurgery Oct 2019;85(4):E756-E764, 2019 Oct.  <https://dx.doi.org/10.1093/neuros/nyz145> | Wrong outcomes |
| 485. | Van Olphen, S.; De Koning, I.; De Grim, L.; Kazemier, G.; Hansen, B.; De Man, R.; Rogiers, X.; Schurink, K.; Metselaar, H., Identification of patients at high risk for infections after liver transplantation; development and validation of a new risk model, Transplant International September 2011;24():176, 2011 September.  <https://dx.doi.org/10.1111/j.1432-2277.2011.01350.x> | Conference abstract |
| 486. | Van Olphen, S.; Konings, I.; De Grim, L.; Kazemier, G.; Hansen, B.; De Man, R.; Rogiers, X.; Schurink, K.; Metselaar, H., Identification of patients at high risk for infections after liver transplantation, Liver Transplantation June 2011;17():S223-S224, 2011 June  <https://dx.doi.org/10.1002/lt.22457> | Conference abstract |
| 487. | van Walraven, C.; Jackson, T. D.; Daneman, N., Administrative data measured surgical site infection probability within 30 days of surgery in elderly patients, J Clin Epidemiol Sep 2016;77():112-117, 2016 Sep.  <https://dx.doi.org/10.1016/j.jclinepi.2016.05.010> | Wrong outcomes |
| 488. | van Walraven C; Jackson TD; Daneman N, Derivation and Validation of the Surgical Site Infections Risk Model Using Health Administrative Data. Infect Control Hosp Epidemiol Apr 2016;37(4):455-65, United States 2016 Apr.  <https://dx.doi.org/10.1017/ice.2015.327> | Wrong outcomes |
| 489. | van Walraven C; Musselman R, The Surgical Site Infection Risk Score (SSIRS): A Model to Predict the Risk of Surgical Site Infections. PLoS One 2013;8(6):e67167, 2013  <https://dx.doi.org/10.1371/journal.pone.0067167> | Timepoint infection not in prediction window |
| 490. | van Winsen M; McSorley ST; McLeod R; MacDonald A; Forshaw MJ; Shaw M; Puxty K, Postoperative C-reactive protein concentrations to predict infective complications following gastrectomy for cancer. J Surg Oncol Dec 2021;124(7):1060-1069, United States 2021 Dec.  <https://dx.doi.org/10.1002/jso.26613> | Timepoint infection not in prediction window |
| 491. | Vaziri, S.; Wilson, J.; Carney, T.; Abbatematteo, J.; Kubilis, P.; Hoh, D., The predictive performance of the american college of surgeons universal risk calculator in neurosurgical patients, Journal of Neurosurgery April 2016;124 (4)():A1181, 2016 April.  <https://dx.doi.org/10.3171/2016.4.JNS.AANS2016abstracts> | Conference abstract |
| 492. | Vegas, A. A.; Jodra, V. M.; Soriano, C.; Lopez, R.; Gil, A.; Garcia, M. L., INFECTION OF THE SURGICAL WOUND - RISK-FACTORS AND PREDICTIVE MODEL, Medicina Clinica Apr 1993;100(14):521-525,  1993 Apr | Not in English |
| 493. | Velazquez N; Press B; Renson A; Wysock JS; Taneja S; Huang WC; Bjurlin MA, Development of a Novel Prognostic Risk Score for Predicting Complications of Penectomy in the Surgical Management of Penile Cancer. Clin Genitourin Cancer Feb 2019;17(1):e123-e129,  United States 2019 Feb.  <https://dx.doi.org/10.1016/j.clgc.2018.09.018> | Wrong outcomes |
| 494. | Venter, D.; Thomas, M.; Lipman, J.; Tang, B.; McLean, A.; Pascoe, R.; Price, G.; Nguyen, T.; Brandon, R.; Sutherland, A., A novel molecular biomarker diagnostic for the early detection of sepsis, Critical Care 01 Sep 2010;14():S3-S4, 2010 01 Sep.  <https://dx.doi.org/10.1186/cc9112> | Conference abstract |
| 495. | Verberk, J.; Van Rooden, S.; Koek, M.; Hopmans, T.; Bonten, M.; De Greeff, S.; Van Mourik, M., Validation of a semiautomated surveillance algorithm for deep surgical site infections after primary total hip or knee arthroplasty - Interim analysis of a multicentre study, Antimicrobial Resistance and Infection Control. Conference: 5th International Conference on Prevention and Infection Control, ICPIC 2019;8(Supplement 1): 2019  <https://dx.doi.org/10.1186/s13756-019-0567-6> | Conference abstract |
| 496. | Verberk JDM; van Rooden SM; Koek MBG; Hetem DJ; Smilde AE; Bril WS; Streefkerk RHRA; Hopmans TEM; Bonten MJM; de Greeff SC; van Mourik MSM, Validation of an algorithm for semiautomated surveillance to detect deep surgical site infections after primary total hip or knee arthroplasty-A multicenter study. Infect Control Hosp Epidemiol Jan 2021;42(1):69-74, United States 2021 Jan  <https://dx.doi.org/10.1017/ice.2020.377> | Timepoint infection not in prediction window |
| 497. | Vinas-Rios JM; Rauschmann M; Sellei R; Arabmotlagh M; Sobotke R; Meyer F, Intra-and postoperative complications in the treatment of spinal metastases. A multicentre surveillance study from the German Spinal Registry (DWG-Register). J Neurosurg Sci Oct 2020;(): Italy 2020 Oct. | Article not available |
| 498. | Walczak, S.; Davila, M. P.; Velanovich, V., Prophylactic Antibiotic Bundle Compliance Does Not Predict Surgical Site Infection: An Artificial Neural Network, Journal of the American College of Surgeons Oct 2018;227(4):E166-E167, 2018 Oct.  <https://dx.doi.org/10.1016/j.jamcollsurg.2018.08.455> | Conference abstract |
| 499. | Walker, L. C.; Clement, N. D.; Hashmi, M.; Samuel, J.; Deehan, D. J., Diagnostic value of routine pre-operative investigations used in combination in the diagnosis of periprosthetic joint infection, Acta Orthop Belg Jun 2021;87(2):374-381, 2021 Jun. | Timepoint infection not specified |
| 500. | Wang, D.; Abuduaini, X.; Huang, X.; Wang, H.; Chen, X.; Le, S.; Chen, M.; Du, X., Development and validation of a risk prediction model for postoperative pneumonia in adult patients undergoing Stanford type A acute aortic dissection surgery: a case control study, J Cardiothorac Surg Feb 23 2022;17(1):22, 2022 Feb 23.  <https://dx.doi.org/10.1186/s13019-022-01769-y> | Duplicate (exact similar publication in other journal) |
| 501. | Wang, D.; Chen, X.; Wu, J.; Le, S.; Xie, F.; Li, X.; Wang, H.; Huang, X.; Zhang, A.; Du, X., Development and Validation of Nomogram Models for Postoperative Pneumonia in Adult Patients Undergoing Elective Cardiac Surgery, Front Cardiovasc Med 2021;8():750828, 2021.  <https://dx.doi.org/10.3389/fcvm.2021.750828> | Timepoint infection not specified |
| 502. | Wang, D.; Huang, X.; Wang, H.; Le, S.; Yang, H.; Wang, F.; Du, X., Risk factors for postoperative pneumonia after cardiac surgery: a prediction model, J Thorac Dis Apr 2021;13(4):2351-2362, 2021 Apr.  <https://dx.doi.org/10.21037/jtd-20-3586> | Does not report (internal) validation performance metric(s) |
| 503. | Wang, D.; Li, Y.; Sheng, W.; Wang, H.; Le, S.; Huang, X.; Du, X., Development and validation of a nomogram model for pneumonia after redo cardiac surgery, J Cardiovasc Med (Hagerstown) Feb 16 2022;(): 2022 Feb 16.  <https://dx.doi.org/10.2459/jcm.0000000000001302> | Timepoint infection not specified |
| 504. | Wang, D. S.; Huang, X. F.; Wang, H. F.; Le, S.; Du, X. L., Clinical risk score for postoperative pneumonia following heart valve surgery, Chin Med J (Engl) Sep 15 2021;134(20):2447-2456, 2021 Sep 15.  <https://dx.doi.org/10.1097/cm9.0000000000001715> | Does not report (internal) validation performance metric(s) |
| 505. | Wang, F.; Hall, N.; Sbitany, H.; Esserman, L., Validation of a NSQIP-based prediction model for surgical site infections after breast reconstruction, Annals of Surgical Oncology April 2017;24 (2 Supplement 1)():52, 2017 April.  <https://dx.doi.org/10.1245/s10434-017-5854-y> | Conference abstract |
| 506. | Wang, H.; Fan, T.; Yang, B.; Lin, Q.; Li, W.; Yang, M., Development and Internal Validation of Supervised Machine Learning Algorithms for Predicting the Risk of Surgical Site Infection Following Minimally Invasive Transforaminal Lumbar Interbody Fusion, Front Med (Lausanne) 2021;8():771608, 2021.  <https://dx.doi.org/10.3389/fmed.2021.771608> | Wrong outcomes |
| 507. | Wang H; Yang J; Jiang L; Wen T; Wang W; Xu M; Li B; Yan L, Development and validation of a prediction score for complications after hepatectomy in hepatitis B-related hepatocellular carcinoma patients. PLoS One 2014;9(8):e105114, 2014.  <https://dx.doi.org/10.1371/journal.pone.0105114> | Wrong outcomes |
| 508. | Wang, P.; Song, W.; Cheng, S.; Shuai, Y.; Yang, J.; Luo, S., Establishment of a Nomogram for Predicting Lumbar Drainage-Related Meningitis: A Simple Tool to Estimate the Infection Risk, Neurocritical Care April 2021;34(2)():557-565, 2021 April.  <https://dx.doi.org/10.1007/s12028-020-01076-1> | Timepoint infection not specified |
| 509. | Wang TK; Choi DH; Haydock D; Gamble G; Stewart R; Ruygrok P, Comparison of Risk Scores for Prediction of Complications following Aortic Valve Replacement. Heart Lung Circ Jun 2015;24(6):595-601,  Australia 2015 Jun.  <https://dx.doi.org/10.1016/j.hlc.2014.11.021> | Timepoint infection not specified |
| 510. | Wang X; Zhang X; Lai X, Exploring an optimal risk adjustment model for public reporting of cesarean section surgical site infections. J Infect Public Health Nov-Dec 2018;11(6):821-825, England 2018 Nov-Dec.  <https://dx.doi.org/10.1016/j.jiph.2018.06.003> | Timepoint infection not specified |
| 511. | Warner JL; Zhang P; Liu J; Alterovitz G, Classification of hospital acquired complications using temporal clinical information from a large electronic health record. J Biomed Inform Feb 2016;59():209-17, 2016 Feb.  <https://dx.doi.org/10.1016/j.jbi.2015.12.008> | Wrong patient population |
| 512. | Watanabe, M.; Suzuki, H.; Nomura, S.; Hanawa, H.; Chihara, N.; Mizutani, S.; Yoshino, M.; Uchida, E., Performance assessment of the risk index category for surgical site infection after colorectal surgery, Surg Infect (Larchmt) Feb 2015;16(1):84-9, 2015 Feb.  <https://dx.doi.org/10.1089/sur.2013.260> | Timepoint infection not in prediction window |
| 513. | Watanabe T; Miyata H; Konno H; Kawai K; Ishihara S; Sunami E; Hirahara N; Wakabayashi G; Gotoh M; Mori M, Prediction model for complications after low anterior resection based on data from 33,411 Japanese patients included in the National Clinical Database. Surgery Jun 2017;161(6):1597-1608, United States 2017 Jun.  <https://dx.doi.org/10.1016/j.surg.2016.12.011> | Timepoint infection not specified |
| 514. | Watcharasin, P.; Vikan, S.; Roong-Rajatavej, P.; Sangsuwan, T.; Jamulitrat, S., Improvement of predictive performance of the new nhsn surgical site infection risk adjustment model for colon surgery by adding variable emergency operation to the model, Antimicrobial Resistance and Infection Control. Conference: International Conference on Prevention and Infection Control, ICPIC 2017;6(Supplement 3): 2017.  <https://dx.doi.org/10.1186/s13756-017-0201-4> | Conference abstract |
| 515. | Wei S; Green C; Kao LS; Padilla-Jones BB; Truong VTT; Wade CE; Harvin JA, Accurate risk stratification for development of organ/space surgical site infections after emergent trauma laparotomy. J Trauma Acute Care Surg Feb 2019;86(2):226-231,  2019 Feb.  <https://dx.doi.org/10.1097/TA.0000000000002143> | Wrong patient population |
| 516. | Welsch T; Müller SA; Ulrich A; Kischlat A; Hinz U; Kienle P; Büchler MW; Schmidt J; Schmied BM, C-reactive protein as early predictor for infectious postoperative complications in rectal surgery. Int J Colorectal Dis Dec 2007;22(12):1499-507, Germany 2007 Dec.  <https://dx.doi.org/10.1007/s00384-007-0354-3> | Timepoint infection not specified |
| 517. | Wen, J.; Pan, T.; Yuan, Y. C.; Huang, Q. S.; Shen, J., Nomogram to predict postoperative infectious complications after surgery for colorectal cancer: a retrospective cohort study in China, World J Surg Oncol Jul 8 2021;19(1):204, 2021 Jul 8.  <https://dx.doi.org/10.1186/s12957-021-02323-1> | Timepoint infection not specified |
| 518. | Wen, R.; Zheng, K.; Zhang, Q.; Zhou, L.; Liu, Q.; Yu, G.; Gao, X.; Hao, L.; Lou, Z.; Zhang, W., Machine learning-based random forest predicts anastomotic leakage after anterior resection for rectal cancer, Journal of Gastrointestinal Oncology June 2021;12(3)():921-932, 2021 June.  <https://dx.doi.org/10.21037/jgo-20-436> | Does not report (internal) validation performance metric(s) |
| 519. | Wierdak M; Pisarska M; Kuśnierz-Cabala B; Witowski J; Major P; Ceranowicz P; Budzyński A; Pędziwiatr M, Serum Amyloid A as an Early Marker of Infectious Complications after Laparoscopic Surgery for Colorectal Cancer. Surg Infect (Larchmt) Aug/Sep 2018;19(6):622-628, United States 2018 Aug/Sep.  <https://dx.doi.org/10.1089/sur.2018.105> | Wrong outcomes |
| 520. | Williams MR; McMeekin S; Wilson RJ; Miller GV; Langlands FE; Wong W; Peter M; Giles MS, Predictive Value of C-Reactive Protein for Complications Post-Laparoscopic Roux-En-Y Gastric Bypass. Obes Surg Mar 2017;27(3):709-715, United States 2017 Mar.  <https://dx.doi.org/10.1007/s11695-016-2349-z> | Wrong outcomes |
| 521. | Williams, R.; Reps, J.; Ying, H. E.; Rijnbeek, P.; Sena, A. G.; Prieto-Alhambra, D.; Ryan, P., Development and validation of patient-level prediction models for adverse outcomes following total knee arthroplasty in osteoarthritic patients, Pharmacoepidemiology and Drug Safety August 2019;28 (Supplement 2)():432, 2019 August.  <https://dx.doi.org/10.1002/pds.4864> | Conference abstract |
| 522. | Wise BT; Connelly D; Rocca M; Mascarenhas D; Huang Y; Maceroli MA; Gage MJ; Joshi M; Castillo RC; OʼToole RV, A Predictive Score for Determining Risk of Surgical Site Infection After Orthopaedic Trauma Surgery. J Orthop Trauma Oct 2019;33(10):506-513, United States 2019 Oct.  <https://dx.doi.org/10.1097/BOT.0000000000001513> | Timepoint infection not in prediction window |
| 523. | Wood CB; Shinn JR; Rees AB; Patel PN; Freundlich RE; Smith DK; McEvoy MD; Rohde SL, Existing Predictive Models for Postoperative Pulmonary Complications Perform Poorly in a Head and Neck Surgery Population. J Med Syst Aug 2019;43(10):312, 2019 Aug.  <https://dx.doi.org/10.1007/s10916-019-1435-x> | Wrong outcomes |
| 524. | Wouthuyzen-Bakker M; Ploegmakers JJW; Kampinga GA; Wagenmakers-Huizenga L; Jutte PC; Muller Kobold AC, Synovial calprotectin: a potential biomarker to exclude a prosthetic joint infection. Bone Joint J May 2017;99-B(5):660-665, England 2017 May.  <https://dx.doi.org/10.1302/0301-620X.99B5.BJJ-2016-0913.R2> | Timepoint infection not in prediction window |
| 525. | Wu, J. M.; Tsai, C. J.; Ho, T. W.; Lai, F. P.; Tai, H. C.; Lin, M. T., A Unified Framework for Automatic Detection of Wound Infection with Artificial Intelligence, Applied Sciences-Basel Aug, 2020;10(15):11, 2020 Aug.  <https://dx.doi.org/10.3390/app10155353> | Timepoint infection not in prediction window |
| 526. | Xiang, G.; Dong, X.; Xu, T.; Feng, Y.; He, Z.; Ke, C.; Xiao, J.; Weng, Y. M., A nomogram for prediction of postoperative pneumonia risk in elderly hip fracture patients, Risk Management and Healthcare Policy 2020;13():1603-1611, 2020.  <https://dx.doi.org/10.2147/RMHP.S270326> | Timepoint infection not specified |
| 527. | Xiao C; Zhou M; Yang X; Xiao F; Liu X; Guo Y; Li X; Cao H; Luo J, Novel nomogram with microvascular density in the surgical margins can accurately predict the risk for anastomotic leakage after anterior resection for rectal cancer. J Surg Oncol Dec 2019;120(8):1412-1419,  United States 2019 Dec.  <https://dx.doi.org/10.1002/jso.25730> | Timepoint infection not in prediction window |
| 528. | Xiao H; Zhou H; Liu K; Liao X; Yan S; Yin B; Ouyang Y, Development and validation of a prognostic nomogram for predicting post-operative pulmonary infection in gastric cancer patients following radical gastrectomy. Sci Rep Oct 2019;9(1):14587, 2019 Oct.  <https://dx.doi.org/10.1038/s41598-019-51227-4> | Timepoint infection not in prediction window |
| 529. | Xie H; Yuan G; Huang S; Kuang J; Yan L; Ruan G; Tang S; Gan J, The prognostic value of combined tumor markers and systemic immune-inflammation index in colorectal cancer patients. Langenbecks Arch Surg Dec 2020;405(8):1119-1130, Germany 2020 Dec.  <https://dx.doi.org/10.1007/s00423-020-01963-3> | Wrong outcomes |
| 530. | Xue, B.; Li, D.; Lu, C.; King, C. R.; Wildes, T.; Avidan, M. S.; Kannampallil, T.; Abraham, J., Use of Machine Learning to Develop and Evaluate Models Using Preoperative and Intraoperative Data to Identify Risks of Postoperative Complications, JAMA Netw Open Mar 1 2021;4(3):e212240, 2021 Mar 1.  <https://dx.doi.org/10.1001/jamanetworkopen.2021.2240> | Timepoint infection not specified |
| 531. | Yamana H; Tsuchiya A; Horiguchi H; Morita S; Kuroki T; Nakai K; Nishimura H; Jo T; Fushimi K; Yasunaga H, Validity of a model using routinely collected data for identifying infections following gastric, colon, and liver cancer surgeries. Pharmacoepidemiol Drug Saf Apr 2022;31(4):452-460, England 2022 Apr.  <https://dx.doi.org/10.1002/pds.5386> | Timepoint infection not specified |
| 532. | Yang M; Li Y; Huang F, A nomogram for predicting postoperative urosepsis following retrograde intrarenal surgery in upper urinary calculi patients with negative preoperative urine culture. Sci Rep Feb 2023;13(1):2123, England 2023 Feb.  <https://dx.doi.org/10.1038/s41598-023-29352-y> | Timepoint infection not in prediction window |
| 533. | Yang PS; Liu CP; Hsu YC; Chen CF; Lee CC; Cheng SP, A Novel Prediction Model for Bloodstream Infections in Hepatobiliary-Pancreatic Surgery Patients. World J Surg May 2019;43(5):1294-1302, United States 2019 May.  <https://dx.doi.org/10.1007/s00268-018-04903-x> | Timepoint infection not in prediction window |
| 534. | Yang Y; Lehman EB; Flohr TR; Radtka JF; Aziz F, Factors associated with symptomatic postoperative myocardial infarction after endovascular aneurysm repair. J Vasc Surg Mar 2020;71(3):806-814,  United States 2020 Mar.  <https://dx.doi.org/10.1016/j.jvs.2019.05.062> | Wrong outcomes |
| 535. | Yao HH; Shao F; Huang Q; Wu Y; Qiang Zhu Z; Liang W, Nomogram to predict anastomotic leakage after laparoscopic anterior resection with intracorporeal rectal transection and double-stapling technique anastomosis for rectal cancer. Hepatogastroenterology Jul-Aug 2014;61(133):1257-61, Greece 2014 Jul-Aug. | Article not available |
| 536. | Yarur, A.; Gondal, B.; Syed, N.; Christensen, B.; Hirsch, A.; Hyman, N.; Abreu, M.; Rubin, D., Validation of a risk score predicting post-surgical complications in patients with inflammatory bowel diseases,  Journal of Crohn's and Colitis February 2015;9():S156-S157, 2015 February  <https://dx.doi.org/10.1093/ecco-jcc/jju027> | Conference abstract |
| 537. | Yegge, J. A.; Gase, K.; Hohrein, M.; Xu, H.; Khoury, R.; Babcock, H., Validation of an electronic algorithm to identify candidates for colon surgical site infection review, American Journal of Infection Control June 2014;42(6):S58, 2014 June.  <https://dx.doi.org/10.1016/j.ajic.2014.03.143> | Conference abstract |
| 538. | Yeo, I.; Klemt, C.; Robinson, M. G.; Esposito, J. G.; Uzosike, A. C.; Kwon, Y. M., The Use of Artificial Neural Networks for the Prediction of Surgical Site Infection Following TKA, J Knee Surg Jan 11 2022;():  2022 Jan 11.  <https://dx.doi.org/10.1055/s-0041-1741396> | Timepoint infection not in prediction window |
| 539. | Yeo I; Klemt C; Robinson MG; Esposito JG; Uzosike AC; Kwon YM, The Use of Artificial Neural Networks for the Prediction of Surgical Site Infection Following TKA., J Knee Surg May 2023;36(6):637-643, Germany 2023 May.  <https://dx.doi.org/10.1055/s-0041-1741396> | Timepoint infection not in prediction window |
| 540. | Yepes-Temiño MJ; Monedero P; Pérez-Valdivieso JR, Risk prediction model for respiratory complications after lung resection: An observational multicentre study., Eur J Anaesthesiol May 2016;33(5):326-33, England 2016 May.  <https://dx.doi.org/10.1097/EJA.0000000000000354> | Wrong outcomes |
| 541. | Yin LX; Chen BM; Zhao GF; Yuan QF; Xue Q; Xu K, Scoring System to Predict the Risk of Surgical Site Infection in Patients with Esophageal Cancer after Esophagectomy with Cervical Anastomosis. Surg Infect (Larchmt) Oct 2018;19(7):696-703, United States 2018 Oct.  <https://dx.doi.org/10.1089/sur.2018.051> | Timepoint infection not in prediction window |
| 542. | Yokoo H; Miyata H; Konno H; Taketomi A; Kakisaka T; Hirahara N; Wakabayashi G; Gotoh M; Mori M, Models predicting the risks of six life-threatening morbidities and bile leakage in 14,970 hepatectomy patients registered in the National Clinical Database of Japan. Medicine (Baltimore) Dec 2016;95(49):e5466, 2016 Dec.  <https://dx.doi.org/10.1097/MD.0000000000005466> | Timepoint infection not specified |
| 543. | Yomayusa, N.; Gaitán, H.; Suárez, I.; Ibáñez, M.; Hernandez, P.; Alvarez, C.; Sossa, M.; Altahona, H.; Arango, A.; Susman, O., Validating prognostic surgical site infection indices from hospitals in Colombia, Rev Salud Publica (Bogota) Nov-Dec 2008;10(5):744-55, 2008 Nov-Dec.  <https://dx.doi.org/10.1590/s0124-00642008000500007> | Not in English |
| 544. | Young, A.; Kaufman, E.; Subramanian, M.; Scantling, D.; Reilly, P.; Sims, C., Predicting sepsis within four hours of admission to a surgical and trauma intensive care unit, Critical Care Medicine January 2021;49(1 SUPPL 1)():611, 2021 January.  <https://dx.doi.org/10.1097/01.ccm.0000730748.10120.55> | Does not report (internal) validation performance metric(s) |
| 545. | Yu, X. Q.; Zhao, B.; Zhou, W. P.; Han, L. Z.; Cai, G. H.; Fang, Z. W.; Peng, Y. H.; Yuan, B., Utility of colon leakage score in left-sided colorectal surgery, J Surg Res May 15 2016;202(2):398-402, 2016 May 15.  <https://dx.doi.org/10.1016/j.jss.2015.12.046> | Timepoint infection not specified |
| 546. | Zabara ML; Popescu I; Burlacu A; Geman O; Dabija RAC; Popa IV; Lupascu C, Machine Learning Model Validated to Predict Outcomes of Liver Transplantation Recipients with Hepatitis C: The Romanian National Transplant Agency Cohort Experience. Sensors (Basel) Feb 2023;23(4): Switzerland 2023 Feb.  <https://dx.doi.org/10.3390/s23042149> | Wrong outcomes |
| 547. | Zhang D; Zhuo H; Yang G; Huang H; Li C; Wang X; Zhao S; Moliterno J; Zhang Y, Postoperative pneumonia after craniotomy: incidence, risk factors and prediction with a nomogram. J Hosp Infect Jun 2020;105(2):167-175, England 2020 Jun.  <https://dx.doi.org/10.1016/j.jhin.2020.03.015> | Wrong outcomes |
| 548. | Zhang H; Meng F; Lu S, Risk factors of sepsis following pancreaticoduodenectomy based on inflammation markers and clinical characteristics., ANZ J Surg Jul 2020;90(7-8):1428-1433, Australia 2020 Jul.  <https://dx.doi.org/10.1111/ans.15791> | Timepoint infection not specified |
| 549. | Zhang H; Meng F; Lu S, Nomograms Predicting the Occurrence of Sepsis in Patients following Major Hepatobiliary and Pancreatic Surgery. Gastroenterol Res Pract 2020;2020():9761878, 2020.  <https://dx.doi.org/10.1155/2020/9761878> | Timepoint infection not specified |
| 550. | Zhang, H. T.; Han, X. K.; Wang, C. S.; Zhang, H.; Li, Z. S.; Chen, Z.; Pan, K.; Zhong, K.; Pan, T.; Wang, D. J., Diagnosis of infection after cardiovascular surgery (DICS): a study protocol for developing and validating a prediction model in prospective observational study, BMJ Open Sep 21 2021;11(9):e048310, 2021 Sep 21.  <https://dx.doi.org/10.1136/bmjopen-2020-048310> | Timepoint infection not in prediction window |
| 551. | Zhang J; Xue F; Liu SD; Liu D; Wu YH; Zhao D; Liu ZM; Ma WX; Han RL; Shan L; Duan XL, Risk factors and prediction model for inpatient surgical site infection after elective abdominal surgery. World J Gastrointest Surg Mar 2023;15(3):387-397, United States 2023 Mar.  <https://dx.doi.org/10.4240/wjgs.v15.i3.387> | Timepoint infection not specified |
| 552. | Zheng H; Wu Z; Wu Y; Mo S; Dai W; Liu F; Xu Y; Cai S, Laparoscopic surgery may decrease the risk of clinical anastomotic leakage and a nomogram to predict anastomotic leakage after anterior resection for rectal cancer. Int J Colorectal Dis Feb 2019;34(2):319-328, 2019 Feb.  <https://dx.doi.org/10.1007/s00384-018-3199-z> | Timepoint infection not specified |
| 553. | Zhong B; Lin ZY; Ma DD; Shang ZH; Shen YB; Zhang T; Zhang JX; Jin WD, A preoperative prediction model based on Lymphocyte-C-reactive protein ratio predicts postoperative anastomotic leakage in patients with colorectal carcinoma: a retrospective study. BMC Surg Jul 2022;22(1):283, England 2022 Jul.  <https://dx.doi.org/10.1186/s12893-022-01734-5> | Timepoint infection not specified |
| 554. | Zhou J; Wu D; Zheng Q; Wang T; Lin M; Lu T; Lin F, A Clinical Prediction Model for Postoperative Pneumonia After Lung Cancer Surgery. J Surg Res Apr 2023;284():62-69, United States 2023 Apr.  <https://dx.doi.org/10.1016/j.jss.2022.11.021> | Timepoint infection not specified |
| 555. | Zhuo Y; Cai D; Chen J; Zhang Q; Li X, Pre-surgical peripheral blood inflammation markers predict surgical site infection following mesh repair of groin hernia. Medicine (Baltimore) Mar 2021;100(9):e25007, United States 2021 Mar.  <https://dx.doi.org/10.1097/MD.0000000000025007> | Timepoint infection not in prediction window |
| 556. | Zivanovic, O.; Yan, J.; Usiak, S.; Lilavois, M.; Ogden, S.; Leitao, M. M.; Sonoda, Y.; Levine, D. A.; Chi, D. S.; Abu-Rustum, N. R., Risk prediction model for surgical site infections in patients undergoing gynecologic cancer surgery, Gynecologic Oncology June 2016;141():166, 2016 June.  <https://dx.doi.org/10.1016/j.ygyno.2016.04.432> | Conference abstract |
| 557. | J Bone Joint Surg Am Mar 17 2021;103(6):532-540, A Machine Learning Algorithm to Identify Patients with Tibial Shaft Fractures at Risk for Infection After Operative Treatment, 2021 Mar 17.  <https://dx.doi.org/10.2106/jbjs.20.00903> | Does not report (internal) validation performance metric(s) |
| 558. | Pietropaolo, A.; Geraghty, R. M.; Veeratterapillay, R.; Rogers, A.; Kallidonis, P.; Villa, L.; Boeri, L.; Montanari, E.; Atis, G.; Emiliani, E.; Sener, T. E.; Al Jaafari, F.; Fitzpatrick, J.; Shaw, M.; Harding, C.; Somani, B. K., A Machine Learning Predictive Model for Post-Ureteroscopy Urosepsis Needing Intensive Care Unit Admission: A Case-Control YAU Endourology Study from Nine European Centres, J Clin Med Aug 29 2021;10(17): 2021 Aug 29.  <https://dx.doi.org/10.3390/jcm10173888> | Timepoint infection not specified |
| 559. | Pedroso-Fernandez, Y.; Aguirre-Jaime, A.; Ramos, M. J.; Hernández, M.; Cuervo, M.; Bravo, A.; Carrillo, A., Prediction of surgical site infection after colorectal surgery, Am J Infect Control Apr 1 2016;44(4):450-4, 2016 Apr 1.  <https://dx.doi.org/10.1016/j.ajic.2015.10.024> | Does not report (internal) validation performance metric(s) |
| 560. | Passias, P. G.; Diebo, B. G.; Marascalchi, B. J.; Jalai, C. M.; Horn, S. R.; Zhou, P. L.; Paltoo, K.; Bono, O. J.; Worley, N.; Poorman, G. W.; Challier, V.; Dixit, A.; Paulino, C.; Lafage, V., A novel index for quantifying the risk of early complications for patients undergoing cervical spine surgeries, J Neurosurg Spine Nov 2017;27(5):501-507, 2017 Nov.  <https://dx.doi.org/10.3171/2017.3.Spine16887> | Wrong outcomes |
| 561. | Ricco, J. B.; Guetarni, F., Use of artificial intelligence and massive data (big data) to identify the risk factors of open surgery to treat infrarenal abdominal aortic aneurysms: A pilot study, Annals of Vascular Surgery October 2020;68():104, 2020 October.  <https://dx.doi.org/10.1016/j.avsg.2020.08.025> | Conference abstract |
| 562. | Reinersman JM; Allen MS; Deschamps C; Ferguson MK; Nichols FC; Shen KR; Wigle DA; Cassivi SD, External validation of the Ferguson pulmonary risk score for predicting major pulmonary complications after oesophagectomy†. Eur J Cardiothorac Surg Jan 2016;49(1):333 8, 2016 Jan.  <https://dx.doi.org/10.1093/ejcts/ezv021> | Wrong outcomes |
| 563. | Regenbogen SE; Read TE; Roberts PL; Marcello PW; Schoetz DJ; Ricciardi R, Urinary tract infection after colon and rectal resections: more common than predicted by risk-adjustment models. J Am Coll Surg Dec 2011;213(6):784-92, United States 2011 Dec.  <https://dx.doi.org/10.1016/j.jamcollsurg.2011.08.013> | Wrong patient population |
| 564. | Sandini, M.; Bernasconi, D. P.; Ippolito, D.; Nespoli, L.; Baini, M.; Barbaro, S.; Fior, D.; Gianotti, L., Preoperative Computed Tomography to Predict and Stratify the Risk of Severe Pancreatic Fistula After Pancreatoduodenectomy, Medicine Aug 2015;94(31):7  2015 Aug.  <https://dx.doi.org/10.1097/md.0000000000001152> | Wrong outcomes |
| 565. | Skube, S. J.; Hu, Z.; Simon, G. J.; Wick, E. C.; Arsoniadis, E. G.; Jensen, E. H.; Kwaan, M. R.; Rothenberger, D. A.; Ko, C. Y.; Melton, G. B., Accelerating American college of surgeons-national surgical quality improvement program surgical site infection abstraction with a semi-automated approach, Journal of the American College of Surgeons October 2017;225 (4 Supplement 1)():S110-S111, 2017 October. | Conference abstract |
| 566. | Siassi M; Riese J; Steffensen R; Meisner M; Thiel S; Hohenberger W; Schmidt J, Mannan-binding lectin and procalcitonin measurement for prediction of postoperative infection. Crit Care Oct 2005;9(5):R483-9, England 2005 Oct.  <https://dx.doi.org/10.1186/cc3768> | Does not report (internal) validation performance metric(s) |
| 567. | Shinkawa H; Takemura S; Uenishi T; Sakae M; Ohata K; Urata Y; Kaneda K; Nozawa A; Kubo S, Nutritional risk index as an independent predictive factor for the development of surgical site infection after pancreaticoduodenectomy. Surg Today Mar 2013;43(3):276-83, Japan 2013 Mar.  <https://dx.doi.org/10.1007/s00595-012-0350-2> | Does not report (internal) validation performance metric(s) |
| 568. | Shen J; Ni Z; Qian Y; Wang B; Zheng S, Clinical prediction score for superficial surgical site infections: Real-life data from a retrospective single-centre analysis of 812 hepatectomies. Int Wound J Feb 2020;17(1):16-20, England 2020 Feb.  <https://dx.doi.org/10.1111/iwj.13209> | Does not report (internal) validation performance metric(s) |
| 569. | Shah, A. A.; Devana, S. K.; Lee, C.; Kianian, R.; van der Schaar, M.; SooHoo, N. F., Development of a Novel, Potentially Universal Machine Learning Algorithm for Prediction of Complications After Total Hip Arthroplasty, Journal of Arthroplasty May 2021;36(5)():1655-1662.e1, 2021 May.  <https://dx.doi.org/10.1016/j.arth.2020.12.040> | Wrong outcomes |
| 570. | Segal CG; Waller DK; Tilley B; Piller L; Bilimoria K, An evaluation of differences in risk factors for individual types of surgical site infections after colon surgery. Surgery Nov 2014;156(5):1253-60, United States 2014 Nov.  <https://dx.doi.org/10.1016/j.surg.2014.05.010> | Does not report (internal) validation performance metric(s) |
| 571. | Subramaniam N; Balasubramanian D; Rka P; Murthy S; Rathod P; Vidhyadharan S; Thankappan K; Iyer S, ACSNSQIP Risk Calculator in Indian Patients Undergoing Surgery for Head and Neck Cancers: Is It Valid? Indian J Surg Oncol Jun 2018;9(2):122-125, 2018 Jun. <https://dx.doi.org/10.1007/s13193-018-0732-z> | Wrong outcomes |
| 572. | Suzuki H; Clore GS; Perencevich EN; Hockett-Sherlock SM; Goto M; Nair R; Branch-Elliman W; Richardson KK; Gupta K; Beck BF; Alexander B; Balkenende EC; Schweizer ML, Development of a fully automated surgical site infection detection algorithm for use in cardiac and orthopedic surgery research. Infect Control Hosp Epidemiol Feb 2021;():1-6, United States 2021 Feb.  <https://dx.doi.org/10.1017/ice.2020.1387> | Wrong outcomes |
| 573. | Suresh KV; Wang K; Sethi I; Zhang B; Margalit A; Puvanesarajah V; Jain A, Spine Surgery and Preoperative Hemoglobin, Hematocrit, and Hemoglobin A1c: A Systematic Review. Global Spine J Jan 2021;():2192568220979821, England 2021 Jan.  <https://dx.doi.org/10.1177/2192568220979821> | Does not report (internal) validation performance metric(s) |
| 574. | Adegboyega, T. O.; Borgert, A. J.; Lambert, P. J.; Jarman, B. T., Applying the National Surgical Quality Improvement Program risk calculator to patients undergoing colorectal surgery: theory vs reality, Am J Surg Jan 2017;213(1):30-35, 2017 Jan.  <https://dx.doi.org/10.1016/j.amjsurg.2016.04.011> | No machine learning model |
| 575. | Alzahrani, S. M.; Ko, C. S.; Yoo, M. W., Validation of the ACS NSQIP surgical risk calculator for patients with early gastric cancer treated with laparoscopic gastrectomy, Journal of Gastric Cancer September 2020;20(3):267-276, 2020 September.  <https://dx.doi.org/10.5230/jgc.2020.20.e27> | No machine learning model |
| 576. | Armstrong EA; Beal EW; Lopez-Aguiar AG; Poultsides G; Cannon JG; Rocha F; Crown A; Barrett J; Ronnkleiv-Kelly S; Fields RC; Krasnick BA; Idrees K; Smith PM; Nathan H; Beems MV; Maithel SK; Schmidt CR; Pawlik TM; Dillhoff M, Evaluating the ACS-NSQIP Risk Calculator in Primary GI Neuroendocrine Tumor: Results from the United States Neuroendocrine Tumor Study Group. Am Surg Dec 2019;85(12):1334-1340, United States 2019 Dec. | No machine learning model |
| 577. | Arozullah, A. M.; Khuri, S. F.; Henderson, W. G.; Daley, J., Development and validation of a multifactorial risk index for predicting postoperative pneumonia after major noncardiac surgery, Ann Intern Med Nov 20 2001;135(10):847-57, 2001 Nov 20.  <https://dx.doi.org/10.7326/0003-4819-135-10-200111200-00005> | No machine learning model |
| 578. | Basta MN; Bauder AR; Kovach SJ; Fischer JP, Assessing the predictive accuracy of the American College of Surgeons National Surgical Quality Improvement Project Surgical Risk Calculator in open ventral hernia repair. Am J Surg Aug 2016;212(2):272-81, United States 2016 Aug.  <https://dx.doi.org/10.1016/j.amjsurg.2016.01.034> | No machine learning model |
| 579. | Bekelis K; Desai A; Bakhoum SF; Missios S, A predictive model of complications after spine surgery: the National Surgical Quality Improvement Program (NSQIP) 2005-2010. Spine J Jul 2014;14(7):1247-55, United States 2014 Jul.  <https://dx.doi.org/10.1016/j.spinee.2013.08.009> | No machine learning model |
| 580. | Benk MS; Olcucuoğlu E; Kaya İO, Evaluation of complications after laparoscopic and open appendectomy by the American College of Surgeons National Surgical Quality Improvement Program surgical risk calculator. Ulus Travma Acil Cerrahi Derg Apr 2022;28(4):418 427, Turkey 2022 Apr.  <https://dx.doi.org/10.14744/tjtes.2020.45808> | No machine learning model |
| 581. | Berger, R. L.; Hicks, S. C.; Davila, J. A.; Li, L. T.; Clapp, M. L.; Berger, D. H.; Liang, M. K., Development and validation of a risk stratification score for identifying patients at increased risk of surgical site infection following open ventral hernia repair, Journal of Surgical Research. Conference: 8th Annual Academic Surgical Congress of the Association for Academic Surgery, AAS and the Society of University Surgeons, SUS. New Orleans, LA United States. Conference Publication: 2013;179(2): 2013. | Timepoint infection not in prediction window |
| 582. | Berger, R. L.; Li, L. T.; Hicks, S. C.; Davila, J. A.; Kao, L. S.; Liang, M. K., Development and validation of a risk-stratification score for surgical site occurrence and surgical site infection after open ventral hernia repair, J Am Coll Surg Dec 2013;217(6):974-82, 2013 Dec.  <https://dx.doi.org/10.1016/j.jamcollsurg.2013.08.003> | No machine learning model |
| 583. | Bergquist JR; Thiels CA; Etzioni DA; Habermann EB; Cima RR, Failure of Colorectal Surgical Site Infection Predictive Models Applied to an Independent Dataset: Do They Add Value or Just Confusion? J Am Coll Surg Apr 2016;222(4):431-8 United States 2016 Apr.  <https://dx.doi.org/10.1016/j.jamcollsurg.2015.12.034> | No machine learning model |
| 584. | Bronheim RS; Oermann EK; Bronheim DS; Caridi JM, Revised Cardiac Risk Index versus ASA Status as a Predictor for Noncardiac Events After Posterior Lumbar Decompression. World Neurosurg Dec 2018;120():e1175-e1184, United States 2018 Dec.  <https://dx.doi.org/10.1016/j.wneu.2018.09.028> | No machine learning model |
| 585. | Cabrera A; Bouterse A; Nelson M; Razzouk J; Ramos O; Chung D; Cheng W; Danisa O, Use of random forest machine learning algorithm to predict short term outcomes following posterior cervical decompression with instrumented fusion. J Clin Neurosci Jan 2023;107():167-171, Scotland 2023 Jan.  <https://dx.doi.org/10.1016/j.jocn.2022.10.029> | Wrong outcomes |
| 586. | Chen LF; Anderson DJ; Kaye KS; Sexton DJ, Validating a 3-point prediction rule for surgical site infection after coronary artery bypass surgery. Infect Control Hosp Epidemiol Jan 2010;31(1):64-8, United States 2010 Jan.  <https://dx.doi.org/10.1086/649019> | No machine learning model |
| 587. | Cheng L; Bai W; Song P; Zhou L; Li Z; Gao L; Zhou C; Cai Q, Development and Validation of a Nomograph Model for Post-Operative Central Nervous System Infection after Craniocerebral Surgery. Diagnostics (Basel) Jun 2023;13(13): Switzerland 2023 Jun.  <https://dx.doi.org/10.3390/diagnostics13132207> | Wrong outcomes |
| 588. | Cheng L; Liu J; Lian L; Duan W; Guan J; Wang K; Liu Z; Wang X; Wang Z; Wu H; Chen Z; Wang J; Jian F, Predicting deep surgical site infection in patients receiving open posterior instrumented thoracolumbar surgery--- A-DOUBLE-SSI risk score: a large retrospective multicenter cohort study in China. Int J Surg May 2023;(): United States 2023 May.  <https://dx.doi.org/10.1097/JS9.0000000000000461> | No machine learning model |
| 589. | Chudgar N; Yan S; Hsu M; Tan KS; Gray KD; Nobel T; Molena D; Sihag S; Bott M; Jones DR; Rusch VW; Rocco G; Isbell JM, External validation of surgical risk preoperative assessment system (SURPAS) in pulmonary resection. Ann Thorac Surg Oct 2020;(): Netherlands 2020 Oct.  <https://dx.doi.org/10.1016/j.athoracsur.2020.08.023> | No machine learning model |
| 590. | Chudgar NP; Yan S; Hsu M; Tan KS; Gray KD; Molena D; Nobel T; Adusumilli PS; Bains M; Downey RJ; Huang J; Park BJ; Rocco G; Rusch VW; Sihag S; Jones DR; Isbell JM, Performance Comparison Between SURPAS and ACS NSQIP Surgical Risk Calculator in Pulmonary Resection. Ann Thorac Surg Oct 2020;(): Netherlands 2020 Oct.  <https://dx.doi.org/10.1016/j.athoracsur.2020.08.021> | No machine learning model |
| 591. | Crispin A; Klinger C; Rieger A; Strahwald B; Lehmann K; Buhr HJ; Mansmann U, The DGAV risk calculator: development and validation of statistical models for a web-based instrument predicting complications of colorectal cancer surgery. Int J Colorectal Dis Oct 2017;32(10):1385-1397, Germany 2017 Oct.  <https://dx.doi.org/10.1007/s00384-017-2869-6> | No machine learning model |
| 592. | Daneman N; Simor AE; Redelmeier DA, Validation of a modified version of the national nosocomial infections surveillance system risk index for health services research. Infect Control Hosp Epidemiol Jun 2009;30(6):563-9, United States 2009 Jun.  <https://dx.doi.org/10.1086/597523> | No machine learning model |
| 593. | Dave A; Beal EW; Lopez-Aguiar AG; Poultsides G; Makris E; Rocha FG; Kanji Z; Ronnekleiv-Kelly S; Rendell VR; Fields RC; Krasnick BA; Idrees K; Smith PM; Nathan H; Beems M; Maithel SK; Pawlik TM; Schmidt CR; Dillhoff ME, Evaluating the ACS NSQIP Risk Calculator in Primary Pancreatic Neuroendocrine Tumor: Results from the US Neuroendocrine Tumor Study Group. J Gastrointest Surg Nov 2019;23(11):2225-2231, United States 2019 Nov.  <https://dx.doi.org/10.1007/s11605-019-04120-4> | No machine learning model |
| 594. | Deek, R. P.; Lee, I. O. K.; van Essen, P.; Crittenden, T.; Dean, N. R., Predicted versus actual complications in Australian women undergoing post-mastectomy breast reconstruction: a retrospective cohort study using the BRA Score tool, J Plast Reconstr Aesthet Surg Dec 2021;74(12):3324-3334, 2021 Dec.  <https://dx.doi.org/10.1016/j.bjps.2021.05.039> | No machine learning model |
| 595. | Donnally CJ; Henstenburg JM; Pezzulo JD; Farronato D; Patel PD; Sherman M; Canseco JA; Kepler CK; Vaccaro AR, Increased Surgical Site Subcutaneous Fat Thickness Is Associated with Infection after Posterior Cervical Fusion. Surg Infect (Larchmt) May 2022;23(4):364-371, United States 2022 May.  <https://dx.doi.org/10.1089/sur.2021.271> | No machine learning model |
| 596. | Edelstein AI; Kwasny MJ; Suleiman LI; Khakhkhar RH; Moore MA; Beal MD; Manning DW, Can the American College of Surgeons Risk Calculator Predict 30-Day Complications After Knee and Hip Arthroplasty? J Arthroplasty Sep 2015;30(9 Suppl):5-10, United States 2015 Sep.  <https://dx.doi.org/10.1016/j.arth.2015.01.057> | No machine learning model |
| 597. | El Asmar A; Hafez K; Fauconnier P; Moreau M; Dal Lago L; Pepersack T; Donckier V; Liberale G, The efficacy of the American College of Surgeons Surgical Risk Calculator in the prediction of postoperative complications in oncogeriatric patients after curative surgery for abdominal tumors., J Surg Oncol Dec 2022;126(7):1359-1366,  United States 2022 Dec.  <https://dx.doi.org/10.1002/jso.27046> | No machine learning model |
| 598. | El Moheb M; Gebran A; Maurer LR; Naar L; El Hechi M; Breen K; Dorken-Gallastegi A; Sinyard R; Bertsimas D; Velmahos G; Kaafarani HMA, Artificial Intelligence versus Surgeon Gestalt in Predicting Risk of Emergency General Surgery. J Trauma Acute Care Surg Jun 2023;(): United States 2023 Jun.  <https://dx.doi.org/10.1097/TA.0000000000004030> | Wrong outcomes |
| 599. | Figuerola-Tejerina, A.; Bustamante, E.; Tamayo, E.; Mestres, C. A.; Bustamante-Munguira, J., Ability to predict the development of surgical site infection in cardiac surgery using the Australian Clinical Risk Index versus the National Nosocomial Infections Surveillance-derived Risk Index, Eur J Clin Microbiol Infect Dis Jun 2017;36(6):1041-1046, 2017 Jun.  <https://dx.doi.org/10.1007/s10096-016-2889-0> | No machine learning model |
| 600. | Gervaz, P.; Bandiera-Clerc, C.; Buchs, N. C.; Eisenring, M. C.; Troillet, N.; Perneger, T.; Harbarth, S., Scoring system to predict the risk of surgical-site infection after colorectal resection, Br J Surg Apr 2012;99(4):589-95, 2012 Apr.  <https://dx.doi.org/10.1002/bjs.8656> | No machine learning model |
| 601. | Goulart A; Ferreira C; Estrada A; Nogueira F; Martins S; Mesquita-Rodrigues A; Sousa N; Leão P, Early Inflammatory Biomarkers as Predictive Factors for Freedom from Infection after Colorectal Cancer Surgery: A Prospective Cohort Study. Surg Infect (Larchmt) May/Jun 2018;19(4):446-450, United States 2018 May/Jun.  <https://dx.doi.org/10.1089/sur.2017.294> | No machine learning model |
| 602. | Grant R; Aupee M; Buchs NC; Cooper K; Eisenring MC; Lamagni T; Ris F; Tanguy J; Troillet N; Harbarth S; Abbas M, Performance of surgical site infection risk prediction models in colorectal surgery: external validity assessment from three European national surveillance networks. Infect Control Hosp Epidemiol Sep 2019;40(9):983-990, United States 2019 Sep.  <https://dx.doi.org/10.1017/ice.2019.163> | No machine learning model |
| 603. | Gupta H; Gupta PK; Schuller D; Fang X; Miller WJ; Modrykamien A; Wichman TO; Morrow LE, Development and validation of a risk calculator for predicting postoperative pneumonia. Mayo Clin Proc Nov 2013;88(11):1241-9, England 2013 Nov.  <https://dx.doi.org/10.1016/j.mayocp.2013.06.027> | No machine learning model |
| 604. | Hamade S; Alshiek J; Javadian P; Ahmed S; McLeod FN; Shobeiri SA, Evaluation of the American College of Surgeons National Surgical Quality Improvement Program Risk Calculator to predict outcomes after hysterectomies. Int J Gynaecol Obstet Sep 2022;158(3):714-721, United States 2022 Sep.  <https://dx.doi.org/10.1002/ijgo.14075> | No machine learning model |
| 605. | Han, K.; Lee, J. M.; Achanta, A.; Kongkaewpaisan, N.; Kongwibulwut, M.; Eid, A. I.; Kokoroskos, N.; van Wijck, S.; Meier, K.; Nordestgaard, A.; Rodriguez, G.; Jia, Z.; Lee, J.; King, D.; Fagenholz, P.; Saillant, N.; Mendoza, A.; Rosenthal, M.; Velmahos, G.; Kaafarani, H. M. A., Emergency Surgery Score Accurately Predicts the Risk of Post-Operative Infection in Emergency General Surgery, Surg Infect (Larchmt) Jan 2019;20(1):4-9, 2019 Jan.  <https://dx.doi.org/10.1089/sur.2018.101> | No machine learning model |
| 606. | Harris AHS; Trickey AW; Eddington HS; Seib CD; Kamal RN; Kuo AC; Ding Q; Giori NJ, A Tool to Estimate Risk of 30-day Mortality and Complications After Hip Fracture Surgery: Accurate Enough for Some but Not All Purposes? A Study From the ACS-NSQIP Database. Clin Orthop Relat Res Dec 2022;480(12):2335-2346, United States 2022 Dec.  <https://dx.doi.org/10.1097/CORR.0000000000002294> | Wrong outcomes |
| 607. | Hedrick TL; Sawyer RG; Friel CM; Stukenborg GJ, A method for estimating the risk of surgical site infection in patients with abdominal colorectal procedures. Dis Colon Rectum May 2013;56(5):627-37, United States 2013 May.  <https://dx.doi.org/10.1097/DCR.0b013e318279a93e> | No machine learning model |
| 608. | Henderson WG; Bronsert MR; Hammermeister KE; Lambert-Kerzner A; Meguid RA, Refining the predictive variables in the "Surgical Risk Preoperative Assessment System" (SURPAS): a descriptive analysis. Patient Saf Surg 2019;13():28, 2019.  <https://dx.doi.org/10.1186/s13037-019-0208-2> | No machine learning model |
| 609. | Hu JS; Huang CB; Mao SM; Fang KH; Wu ZY; Zhao YM, Development of a nomogram to predict surgical site infection after closed comminuted calcaneal fracture. BMC Surg Aug 2022;22(1):313, England 2022 Aug.  <https://dx.doi.org/10.1186/s12893-022-01735-4> | No machine learning model |
| 610. | Jonczyk MM; Fisher CS; Babbitt R; Paulus JK; Freund KM; Czerniecki B; Margenthaler JA; Losken A; Chatterjee A, Surgical Predictive Model for Breast Cancer Patients Assessing Acute Postoperative Complications: The Breast Cancer Surgery Risk Calculator. Ann Surg Oncol Feb 2021;(): United States 2021 Feb.  <https://dx.doi.org/10.1245/s10434-021-09710-8> | No machine learning model |
| 611. | Kawai K; Hirakawa S; Tachimori H; Oshikiri T; Miyata H; Kakeji Y; Kitagawa Y, Updating the predictive models for mortality and morbidity after low anterior resection based on the National Clinical Database. Dig Surg Jun 2023;(): Switzerland 2023 Jun.  <https://dx.doi.org/10.1159/000531370> | No machine learning model |
| 612. | Kikuchi H; Miyata H; Konno H; Kamiya K; Tomotaki A; Gotoh M; Wakabayashi G; Mori M, Development and external validation of preoperative risk models for operative morbidities after total gastrectomy using a Japanese web-based nationwide registry. Gastric Cancer Nov 2017;20(6):987-997, 2017 Nov.  <https://dx.doi.org/10.1007/s10120-017-0706-9> | No machine learning model |
| 613. | Kinlin, L. M.; Kirchner, C.; Zhang, H.; Daley, J.; Fisman, D. N., Derivation and validation of a clinical prediction rule for nosocomial pneumonia after coronary artery bypass graft surgery, Clinical Infectious Diseases 15 Feb 2010;50(4):493-501, 2010 15 Feb.  <https://dx.doi.org/10.1086/649925> | No machine learning model |
| 614. | Kirmani, B. H.; Mazhar, K.; Saleh, H. Z.; Ward, A. N.; Shaw, M.; Fabri, B. M.; Mark Pullan, D., External validity of the Society of Thoracic Surgeons risk stratification tool for deep sternal wound infection after cardiac surgery in a UK population, Interact Cardiovasc Thorac Surg Sep 2013;17(3):479-84, 2013 Sep.  <https://dx.doi.org/10.1093/icvts/ivt222> | No machine learning model |
| 615. | Klemencsics I; Lazary A; Szoverfi Z; Bozsodi A; Eltes P; Varga PP, Risk factors for surgical site infection in elective routine degenerative lumbar surgeries. Spine J Nov 2016;16(11):1377-1383, United States 2016 Nov.  <https://dx.doi.org/10.1016/j.spinee.2016.08.018> | No machine learning model |
| 616. | Lee T; Hwang EJ; Park CM; Goo JM, Deep Learning-Based Computer-Aided Detection System for Preoperative Chest Radiographs to Predict Postoperative Pneumonia. Acad Radiol Mar 2023;(): United States 2023 Mar.  <https://dx.doi.org/10.1016/j.acra.2023.02.016> | Wrong oucomes |
| 617. | Leekha S; Lahr BD; Thompson RL; Sampathkumar P; Duncan AA; Orenstein R, Preoperative risk prediction of surgical site infection requiring hospitalization or reoperation in patients undergoing vascular surgery. J Vasc Surg Jul 2016;64(1):177-84, United States 2016 Jul.  <https://dx.doi.org/10.1016/j.jvs.2016.01.029> | No machine learning model |
| 618. | Li L; Ding J; Han J; Wu H, A nomogram prediction of postoperative surgical site infections in patients with perihilar cholangiocarcinoma.  Medicine (Baltimore) Jun 2017;96(25):e7198, 2017 Jun.  <https://dx.doi.org/10.1097/MD.0000000000007198> | No machine learning model |
| 619. | Lian J; Wang Y; Yan X; Xu G; Jia M; Yang J; Ying J; Teng H, Development and validation of a nomogram to predict the risk of surgical site infection within 1 month after transforaminal lumbar interbody fusion. J Orthop Surg Res Feb 2023;18(1):105, England 2023 Feb.  <https://dx.doi.org/10.1186/s13018-023-03550-w> | No machine learning model |
| 620. | Liang MK; Goodenough CJ; Martindale RG; Roth JS; Kao LS, External validation of the ventral hernia risk score for prediction of surgical site infections. Surg Infect (Larchmt) Feb 2015;16(1):36-40, 2015 Feb.  <https://dx.doi.org/10.1089/sur.2014.115> | No machine learning model |
| 621. | Lone Z; Hall S; Terakawa T; Ahmed YE; Elsayed AS; Aldhaam N; May PR; Miller A; Jing Z; Bragayrac LN; Khan H; Cohen J; Cole A; Rana O; Kanapan R; Prechtl C; Hussein AA; Guru KA, Accuracy of American College of Surgeons National Surgical Quality Improvement Program Universal Surgical Risk Calculator in Predicting Complications Following Robot-Assisted Radical Cystectomy at a National Comprehensive Cancer Center. J Endourol May 2019;33(5):383-388, United States 2019 May.  <https://dx.doi.org/10.1089/end.2019.0093> | No machine learning model |
| 622. | Long AM; Hildreth AN; Davis PT; Ur R; Badger AT; Miller PR, Evaluation of the Performance of ACS NSQIP Surgical Risk Calculator in Emergency General Surgery Patients. Am Surg Feb 2020;86(2):83-89, United States 2020 Feb. | No machine learning model |
| 623. | Mannas MP; Lee T; Forbes CM; Hong T; Bisaillon A; Gleave ME; So AI; Mayson K; Black PC, Predicting complications following radical cystectomy with the ACS NSQIP universal surgical risk calculator. World J Urol May 2020;38(5):1215-1220, Germany 2020 May.  <https://dx.doi.org/10.1007/s00345-019-02915-3> | No machine learning model |
| 624. | Martin S; Turner E; Nguyen A; Thornton B; Nazerali RS, An Evaluation of the Utility of the Breast Reconstruction Risk Assessment Score Risk Model in Prepectoral Tissue Expander Breast Reconstruction. Ann Plast Surg May 2020;84(5S Suppl 4):S318-S322,  United States 2020 May.  <https://dx.doi.org/10.1097/SAP.0000000000002320> | No machine learning model |
| 625. | McCarthy MH; Singh P; Maslak J; Nayak R; Jenkins TJ; Hsu WK; Patel AA, Can the American College of Surgeons Risk Calculator Predict 30-Day Complications After Cervical Spine Surgery? Clin Spine Surg Nov 2019;32(9):357-362, United States 2019 Nov.  <https://dx.doi.org/10.1097/BSD.0000000000000890> | No machine learning model |
| 626. | McKenna NP; Bews KA; Cima RR; Crowson CS; Habermann EB, Development of a Risk Score to Predict Anastomotic Leak After Left-Sided Colectomy: Which Patients Warrant Diversion? J Gastrointest Surg Jan 2020;24(1):132-143, United States 2020 Jan.  <https://dx.doi.org/10.1007/s11605-019-04293-y> | No machine learning model |
| 627. | Meguid RA; Bronsert MR; Juarez-Colunga E; Hammermeister KE; Henderson WG, Surgical Risk Preoperative Assessment System (SURPAS): II. Parsimonious Risk Models for Postoperative Adverse Outcomes Addressing Need for Laboratory Variables and Surgeon Specialty-specific Models. Ann Surg Jul 2016;264(1):10-22, United States 2016 Jul.  <https://dx.doi.org/10.1097/SLA.0000000000001677> | No machine learning model |
| 628. | Menezes, A. S.; Fernandes, A.; Rodrigues, J. R.; Salome, C.; Machado, F.; Antunes, L.; Silva, J. C.; Monteiro, E.; Santos, L. L., European Archives of Oto-Rhino-Laryngology Jan 2021;278(1):191-202, 2021 Jan.  <https://dx.doi.org/10.1007/s00405-020-06133-1> | No machine learning model |
| 629. | Mitchell TO; Holihan JL; Askenasy EP; Greenberg JA; Keith JN; Martindale RG; Roth JS; Liang MK, Do risk calculators accurately predict surgical site occurrences? J Surg Res Jun 2016;203(1):56-63,  United States 2016 Jun.  <https://dx.doi.org/10.1016/j.jss.2016.03.040> | No machine learning model |
| 630. | Miyakita H; Sadahiro S; Saito G; Okada K; Tanaka A; Suzuki T, Risk scores as useful predictors of perioperative complications in patients with rectal cancer who received radical surgery. Int J Clin Oncol Apr 2017;22(2):324-331, 2017 Apr.  <https://dx.doi.org/10.1007/s10147-016-1054-1> | No machine learning model |
| 631. | Narain AS; Kitto AZ; Braun B; Poorman MJ; Curtin P; Slavin J; Whalen G; DiPaola CP; Connolly PJ; Stauff MP, Does the ACS NSQIP Surgical Risk Calculator Accurately Predict Complications Rates After Anterior Lumbar Interbody Fusion Procedures? Spine (Phila Pa 1976) Dec 2020;Publish Ahead of Print(): United States 2020 Dec.  <https://dx.doi.org/10.1097/BRS.0000000000003893> | No machine learning model |
| 632. | Neumayer L; Hosokawa P; Itani K; El-Tamer M; Henderson WG; Khuri SF, Multivariable predictors of postoperative surgical site infection after general and vascular surgery: results from the patient safety in surgery study. J Am Coll Surg Jun 2007;204(6):1178-87, United States 2007 Jun.  <https://dx.doi.org/10.1016/j.jamcollsurg.2007.03.022> | No machine learning model |
| 633. | Ohkura Y; Miyata H; Konno H; Udagawa H; Ueno M; Shindoh J; Kumamaru H; Wakabayashi G; Gotoh M; Mori M, Development of a model predicting the risk of eight major postoperative complications after esophagectomy based on 10 826 cases in the Japan National Clinical Database. J Surg Oncol Dec 2019;(): United States 2019 Dec.  <https://dx.doi.org/10.1002/jso.25800> | No machine learning model |
| 634. | Orlandi BMM; Mejia OAV; Sorio JL; de Barros E Silva P; Oliveira MAP; Nakazone MA; Tiveron MG; Campagnucci VP; Lisboa LAF; Zubelli J; Normand SL; Jatene FB, Performance of a novel risk model for deep sternal wound infection after coronary artery bypass grafting. Sci Rep Sep 2022;12(1):15177, England 2022 Sep.  <https://dx.doi.org/10.1038/s41598-022-19473-1> | Timepoint infection not in prediction window |
| 635. | Poruk, K. E.; Hicks, C. W.; Trent Magruder, J.; Rodriguez-Unda, N.; Burce, K. K.; Azoury, S. C.; Cornell, P.; Cooney, C. M.; Eckhauser, F. E., Creation of a novel risk score for surgical site infection and occurrence after ventral hernia repair, Hernia 2016;():1‐9, 2016.  <https://dx.doi.org/10.1007/s10029-016-1547-x> | No machine learning model |
| 636. | Poruk KE; Lin JA; Cooper MA; He J; Makary MA; Hirose K; Cameron JL; Pawlik TM; Wolfgang CL; Eckhauser F; Weiss MJ, A novel, validated risk score to predict surgical site infection after pancreaticoduodenectomy. HPB (Oxford) Nov 2016;18(11):893-899,  2016 Nov.  <https://dx.doi.org/10.1016/j.hpb.2016.07.011> | No machine learning model |
| 637. | Prasad KG; Nelson BG; Deig CR; Schneider AL; Moore MG, ACS NSQIP Risk Calculator: An Accurate Predictor of Complications in Major Head and Neck Surgery? Otolaryngol Head Neck Surg Nov 2016;155(5):740-742, England 2016 Nov.  <https://dx.doi.org/10.1177/0194599816655976> | No machine learning model |
| 638. | Ravindran, K.; Escobar, D.; Gautam, S.; Puri, R.; Awad, Z., Assessment of the American College of Surgeons National Surgical Quality Improvement Program Calculator in Predicting Outcomes and Length of Stay After Ivor Lewis Esophagectomy: A Single-Center Experience, Journal of Surgical Research November 2020;255():355-360, 2020 November.  <https://dx.doi.org/10.1016/j.jss.2020.05.080> | No machine learning model |
| 639. | Ren Y; Loftus TJ; Datta S; Ruppert MM; Guan Z; Miao S; Shickel B; Feng Z; Giordano C; Upchurch GR Jr; Rashidi P; Ozrazgat-Baslanti T; Bihorac A, Performance of a Machine Learning Algorithm Using Electronic Health Record Data to Predict Postoperative Complications and Report on a Mobile Platform. JAMA Netw Open May 2022;5(5):e2211973, United States 2022 May.  <https://dx.doi.org/10.1001/jamanetworkopen.2022.11973> | Timepoint infection not in prediction window |
| 640. | Rencuzogullari, A.; Benlice, C.; Valente, M.; Abbas, M. A.; Remzi, F. H.; Gorgun, E., Predictors of Anastomotic Leak in Elderly Patients After Colectomy: Nomogram-Based Assessment From the American College of Surgeons National Surgical Quality Program Procedure-Targeted Cohort, Dis Colon Rectum May 2017;60(5):527-536, 2017 May.  <https://dx.doi.org/10.1097/dcr.0000000000000789> | No machine learning model |
| 641. | Rivard C; Nahum R; Slagle E; Duininck M; Isaksson Vogel R; Teoh D, Evaluation of the performance of the ACS NSQIP surgical risk calculator in gynecologic oncology patients undergoing laparotomy. Gynecol Oncol May 2016;141(2):281-286, 2016 May.  <https://dx.doi.org/10.1016/j.ygyno.2016.02.015> | No machine learning model |
| 642. | Sangsuwan T; Jamulitrat S; Watcharasin P, Risk adjustment performance between NNIS index and NHSN model for postoperative colorectal surgical site infection: A retrospective cohort study. Ann Med Surg (Lond) May 2022;77():103715, England 2022 May.  <https://dx.doi.org/10.1016/j.amsu.2022.103715> | No machine learning model |
| 643. | Scepanovic MS; Kovacevic B; Cijan V; Antic A; Petrovic Z; Asceric R; Krdzic I; Cuk V, C-reactive protein as an early predictor for anastomotic leakage in elective abdominal surgery. Tech Coloproctol Oct 2013;17(5):541-7, Italy 2013 Oct.  <https://dx.doi.org/10.1007/s10151-013-1013-z> | No machine learning model |
| 644. | Schneider, A. L.; Deig, C. R.; Prasad, K. G.; Nelson, B. G.; Mantravadi, A. V.; Brigance, J. S.; Langer, M. P.; McDonald, M. W.; Johnstone, P. A.; Moore, M. G., Ability of the National Surgical Quality Improvement Program Risk Calculator to Predict Complications Following Total Laryngectomy, JAMA Otolaryngol Head Neck Surg Oct 1 2016;142(10):972-979, 2016 Oct 1.  <https://dx.doi.org/10.1001/jamaoto.2016.1809> | No machine learning model |
| 645. | Scotton G; Del Zotto G; Bernardi L; Zucca A; Terranova S; Fracon S; Paiano L; Cosola D; Biloslavo A; de Manzini N, Is the ACS-NSQIP Risk Calculator Accurate in Predicting Adverse Postoperative Outcomes in the Emergency Setting? An Italian Single-center Preliminary Study. World J Surg Nov 2020;44(11):3710-3719, 2020 Nov.  <https://dx.doi.org/10.1007/s00268-020-05705-w> | No machine learning model |
| 646. | Scotton G; La Greca A; Lirusso C; Mariani D; Zago M; Chiarugi M; Tartaglia D; de Manzini N; Biloslavo A, Can the American College of Surgeons NSQIP Surgical Risk Calculator Accurately Predict Adverse Postoperative Outcomes in Emergency Abdominal Surgery? An Italian Multicenter Analysis. J Am Coll Surg Feb 2023;236(2):387-398, United States 2023 Feb.  <https://dx.doi.org/10.1097/XCS.0000000000000445> | No machine learning model |
| 647. | Stidham K; Naftchi AF; Spirollari E; Vaserman G; Vazquez S; Das A; Colasacco C; Culbertson S; Ng C; Graifman G; Beaudreault C; Lui AK; Dominguez JF; Kazim SF; Schmidt M; Bowers CA, Frailty Is Superior to Age for Predicting Readmission, Prolonged Length of Stay, and Wound Infection in Elective Otology Procedures. Otol Neurotol Sep 2022;43(8):937-943, United States 2022 Sep.  <https://dx.doi.org/10.1097/MAO.0000000000003636> | No machine learning model |
| 648. | Stidham RW; Waljee AK; Day NM; Bergmans CL; Zahn KM; Higgins PD; Wang SC; Su GL, Body fat composition assessment using analytic morphomics predicts infectious complications after bowel resection in Crohn's disease. Inflamm Bowel Dis Jun 2015;21(6):1306-13, 2015 Jun.  <https://dx.doi.org/10.1097/MIB.0000000000000360> | No machine learning model |
| 649. | Szender JB; Frederick PJ; Eng KH; Akers SN; Lele SB; Odunsi K, Evaluation of the National Surgical Quality Improvement Program Universal Surgical Risk Calculator for a gynecologic oncology service. Int J Gynecol Cancer Mar 2015;25(3):512-20, 2015 Mar.  <https://dx.doi.org/10.1097/IGC.0000000000000378> | No machine learning model |
| 650. | Tam S; Dong W; Adelman DM; Weber RS; Lewis CM, Risk-adjustment models in patients undergoing head and neck surgery with reconstruction. Oral Oncol Dec 2020;111():104917, England 2020 Dec.  <https://dx.doi.org/10.1016/j.oraloncology.2020.104917> | No machine learning model |
| 651. | Tierney W; Shah J; Clancy K; Lee MY; Ciolek PJ; Fritz MA; Lamarre ED, Predictive value of the ACS NSQIP calculator for head and neck reconstruction free tissue transfer. Laryngoscope Mar 2020;130(3):679-684, United States 2020 Mar.  <https://dx.doi.org/10.1002/lary.28195> | No machine learning model |
| 652. | Tourani R; Murphree DH; Melton-Meaux G; Wick E; Kor DJ; Simon GJ, The Value of Aggregated High-Resolution Intraoperative Data for Predicting Post-Surgical Infectious Complications at Two Independent Sites. Stud Health Technol Inform Aug 2019;264():398-402, 2019 Aug.  <https://dx.doi.org/10.3233/SHTI190251> | No machine learning model |
| 653. | Trickey AW; Ding Q; Harris AHS, How Accurate Are the Surgical Risk Preoperative Assessment System (SURPAS) Universal Calculators in Total Joint Arthroplasty? Clin Orthop Relat Res Feb 2020;478(2):241-251, 2020 Feb.  <https://dx.doi.org/10.1097/CORR.0000000000001078> | No machine learning model |
| 654. | van der Hulst HC; Dekker JWT; Bastiaannet E; van der Bol JM; van den Bos F; Hamaker ME; Schiphorst A; Sonneveld DJA; Schuijtemaker JS; de Jong RJ; Portielje JEA; Souwer ETD, Validation of the ACS NSQIP surgical risk calculator in older patients with colorectal cancer undergoing elective surgery. J Geriatr Oncol Jul 2022;13(6):788-795, Netherlands 2022 Jul.  <https://dx.doi.org/10.1016/j.jgo.2022.04.004> | No machine learning model |
| 655. | Varetto G; Castagno C; Trucco A; Frola E; Bert F; Scozzari G; Rispoli P, Serum Procalcitonin as a Valuable Diagnostic Tool in the Early Detection of Infectious Complications after Open Abdominal Aortic Repair. Ann Vasc Surg Jul 2016;34():111-8, Netherlands 2016 Jul.  <https://dx.doi.org/10.1016/j.avsg.2016.01.012> | No machine learning model |
| 656. | Vaziri S; Wilson J; Abbatematteo J; Kubilis P; Chakraborty S; Kshitij K; Hoh DJ, Predictive performance of the American College of Surgeons universal risk calculator in neurosurgical patients. J Neurosurg Mar 2018;128(3):942-947, United States 2018 Mar.  <https://dx.doi.org/10.3171/2016.11.JNS161377> | No machine learning model |
| 657. | Vosler, P. S.; Orsini, M.; Enepekides, D. J.; Higgins, K. M., Predicting complications of major head and neck oncological surgery: an evaluation of the ACS NSQIP surgical risk calculator, J Otolaryngol Head Neck Surg Mar 22 2018;47(1):21, 2018 Mar 22.  <https://dx.doi.org/10.1186/s40463-018-0269-8> | No machine learning model |
| 658. | Vu MM; Ellis MF; Blough JT; Gutowski KA; Kim JYS, Development and Internal Validation of the Abdominoplasty Risk Calculator. Plast Reconstr Surg Jan 2018;141(1):34e-45e, United States 2018 Jan.  <https://dx.doi.org/10.1097/PRS.0000000000003922> | No machine learning model |
| 659. | Wherley, S. D.; Chapman, G. C.; Mahajan, S. T.; Hijaz, A. K.; Slopnick, E. A.; Roberts, K.; El-Nashar, S., Evaluation of the ACS NSQIP surgical risk calculator in patients undergoing pelvic organ prolapse surgery, International Urogynecology Journal. 2020;(): 2020.  <https://dx.doi.org/10.1007/s00192-020-04364-8> | No machine learning model |
| 660. | Wingert NC; Gotoff J; Parrilla E; Gotoff R; Hou L; Ghanem E, The ACS NSQIP Risk Calculator Is a Fair Predictor of Acute Periprosthetic Joint Infection. Clin Orthop Relat Res Jul 2016;474(7):1643-8, 2016 Jul.  <https://dx.doi.org/10.1007/s11999-016-4717-3> | No machine learning model |
| 661. | Winoker JS; Paulucci DJ; Anastos H; Waingankar N; Abaza R; Eun DD; Bhandari A; Hemal AK; Sfakianos JP; Badani KK, Predicting Complications Following Robot-Assisted Partial Nephrectomy with the ACS NSQIP(®) Universal Surgical Risk Calculator. J Urol Oct 2017;198(4):803-809, United States 2017 Oct.  <https://dx.doi.org/10.1016/j.juro.2017.04.021> | No machine learning model |
| 662. | Xun Y; Yang Y; Yu X; Li C; Lu J; Wang S, A preoperative nomogram for sepsis in percutaneous nephrolithotomy treating solitary, unilateral and proximal ureteral stones. PeerJ 2020;8():e9435, 2020.  <https://dx.doi.org/10.7717/peerj.9435> | No machine learning model |
| 663. | Yap MKC; Ang KF; Gonzales-Porciuncula LA; Esposo E, Validation of the American College of Surgeons Risk Calculator for preoperative risk stratification. Heart Asia 2018;10(2):e010993, 2018.  <https://dx.doi.org/10.1136/heartasia-2017-010993> | No machine learning model |
| 664. | Yoshida T; Miyata H; Konno H; Kumamaru H; Tangoku A; Furukita Y; Hirahara N; Wakabayashi G; Gotoh M; Mori M, Risk assessment of morbidities after right hemicolectomy based on the National Clinical Database in Japan. Ann Gastroenterol Surg May 2018;2(3):220-230,  2018 May.  <https://dx.doi.org/10.1002/ags3.12067> | No machine learning model |
| 665. | Zhang D; Ren J; Arafeh MO; Sawyer RG; Hu Q; Wu X; Wang G; Gu G; Hu J; Li M, The Significance of Interleukin-6 in the Early Detection of Surgical Site Infections after Definitive Operation for Gastrointestinal Fistulae. Surg Infect (Larchmt) Jul 2018;19(5):523-528, United States 2018 Jul.  <https://dx.doi.org/10.1089/sur.2017.271> | No machine learning model |
| 666. | Ren Y; Loftus TJ; Datta S; Ruppert MM; Guan Z; Miao S; Shickel B; Feng Z; Giordano C; Upchurch GR Jr; Rashidi P; Ozrazgat-Baslanti T; Bihorac A, Performance of a Machine Learning Algorithm Using Electronic Health Record Data to Predict Postoperative Complications and Report on a Mobile Platform. JAMA Netw Open May 2022;5(5):e2211973, United States 2022 May.  <https://dx.doi.org/10.1001/jamanetworkopen.2022.11973> | Timepoint infection not in prediction window |
| 667. | Chen Z; Zhong M; Xu Z; Ye Q; Xie W; Gao S; Chen L; Qiu L; Jiang J; Wu H; Li X; Wang H, Development and Validation of a Nomogram Based on Geriatric Nutritional Risk Index to Predict Surgical Site Infection Among Gynecologic Oncology Patients. Front Nutr 2022;9():864761, Switzerland 2022.  <https://dx.doi.org/10.3389/fnut.2022.864761> | No machine learning model |
| 668. | Cheng X; Liu Y; Wang W; Yan J; Lei X; Wu H; Zhang Y; Zhu Y, Preoperative Risk Factor Analysis and Dynamic Online Nomogram Development for Early Infections Following Primary Hip Arthroplasty in Geriatric Patients with Hip Fracture, Clin Interv Aging 2022;17():1873-1883, New Zealand 2022.  <https://dx.doi.org/10.2147/CIA.S392393> | Timepoint infection not in prediction window |
| 669. | Cheng Y; Chen Y; Hou X; Yu J; Wen H; Dai J; Zheng Y, Development of a Nomogram for Predicting Surgical Site Infection in Patients with Resected Lung Neoplasm Undergoing Minimally Invasive Surgery. Surg Infect (Larchmt) Oct 2022;23(8):754-762, United States 2022 Oct.  <https://dx.doi.org/10.1089/sur.2022.166> | Timepoint infection not in prediction window |
| 670. | Gowd AK; O'Neill CN; Barghi A; O'Gara TJ; Carmouche JJ, Feasibility of Machine Learning in the Prediction of Short-Term Outcomes Following Anterior Cervical Discectomy and Fusion. World Neurosurg Dec 2022;168():e223-e232, United States 2022 Dec.  <https://dx.doi.org/10.1016/j.wneu.2022.09.090> | Timepoint infection not in prediction window |
| 671. | Shi J; Wu Z; Wu X; Shan F; Zhang Y; Ying X; Li Z; Ji J, Early diagnosis of anastomotic leakage after colorectal cancer surgery using an inflammatory factors-based score system. BJS Open May 2022;6(3):  England 2022 May.  <https://dx.doi.org/10.1093/bjsopen/zrac069> | No machine learning model |
| 672. | Shimizu T; Endo Y; Tabata T; Mori T; Hanasawa K; Tsuchiya M; Tani T, Diagnostic and predictive value of the silkworm larvae plasma test for postoperative infection following gastrointestinal surgery. Crit Care Med Jun 2005;33(6):1288-95, United States 2005 Jun.  <https://dx.doi.org/10.1097/01.ccm.0000165810.97971.dd> | Wrong outcomes |
| 673. | van Kooten RT; Bahadoer RR; Ter Buurkes de Vries B; Wouters MWJM; Tollenaar RAEM; Hartgrink HH; Putter H; Dikken JL, Conventional regression analysis and machine learning in prediction of anastomotic leakage and pulmonary complications after esophagogastric cancer surgery. J Surg Oncol Sep 2022;126(3):490-501, United States 2022 Sep. | Timepoint infection not in prediction window |
| 674. | Zhou Y; Wang L; Cao A; Luo W; Xu Z; Sheng Z; Wang J; Zhu B, Modified Frailty Index Combined with a Prognostic Nutritional Index for Predicting Postoperative Complications of Hip Fracture Surgery in Elderly. J Invest Surg Oct 2022;35(10):1739-1746, United States 2022 Oct.  <https://dx.doi.org/10.1080/08941939.2022.2101166> | No machine learning model |
