# Supplementary material for: Systematic evaluation of machine learning models for postoperative surgical site infection prediction
Source: PLoS One. 2024 Dec 12;19(12):e0312968. doi: 10.1371/journal.pone.0312968 (PMC11637340; doi:10.1371/journal.pone.0312968)
Supplement: S2 Table — (DOCX) [file pone.0312968.s004.docx]

# S2 Table. Extracted parameters from the data

| Article information | First author, last author, title article, year of publication |
| --- | --- |
| Model information | Name of prediction tool, type of prediction tool (machine learning vs non-machine learning), type of infection predicted, exact definition infection, number of predictors used, predictors used and their weighting in the model, moment of prediction, window of prediction |
| Population | Surgical specialty, number of included patients, age of patients |
| Outcomes | Type of validation, sensitivity, specificity, positive predictive value, negative predictive value, net reclassification impact, area under the receiver operating curve, calibration, brier score, other performance measures |
