# Supplementary material for: Systematic evaluation of machine learning models for postoperative surgical site infection prediction
Source: PLoS One. 2024 Dec 12;19(12):e0312968. doi: 10.1371/journal.pone.0312968 (PMC11637340; doi:10.1371/journal.pone.0312968)
Supplement: S3 Table — (DOCX) [file pone.0312968.s005.docx]

# S3 Table. Studies with both ML and regression-based models.

| Author & year | Prediction tool | Specialty | Infection predicted | Sample size (n) | C-statistic | Validation |
| --- | --- | --- | --- | --- | --- | --- |
| Bonde  2020 (21) | ACS-NSQIP risk calculator | General surgery | SSI | 5,881,881 | 0.87 | External |
|  | ANN model 1 | General surgery | SSI; superficial SSI; deep SSI; organ space SSI |  | 0.85; 0.82; 0.78; 0.85 | External |
|  |  | Emergency surgery | Superficial SSI; deep SSI; organ space SSI |  | 0.75; 0.87; 0.78 | External |
|  | ANN model 2 | General surgery | SSI; superficial SSI; deep SSI; organ space SSI |  | 0.85; 0.82; 0.78; 0.85 | External |
|  |  | Emergency surgery | Superficial SSI; deep SSI; organ space SSI |  | 0.75; 0.85; 0.81 | External |
|  | ANN model 3 | General surgery | SSI; superficial SSI; deep SSI; organ space SSI |  | 0.86; 0.83; 0.80;  0.87 | External |
|  |  | Emergency surgery | Superficial SSI; deep SSI; organ space SSI |  | 0.76; 0.82; 0.86 | External |
|  | OCT POTTER | Emergency surgery | Superficial SSI; deep SSI; organ space SSI |  | 0.68; 0.75; 0.79 | External |
| Gowd  2019 (24) | ASA classification  Frailty index  Logistic regression | Orthopedic surgery | SSI | 17,119 | 0.58  0.41  0.65 | External |
|  | KNN |  |  |  | 0.5 | Internal |
|  | RF |  |  |  | 0.53 | Internal |
|  | NB |  |  |  | 0.45 | Internal |
|  | Decision tree |  |  |  | 0.5 | Internal |
|  | Gradient boosting trees |  |  |  | 0.61 | Internal |
| Grass  2021 (25) | GLM | Abdominal surgery | SSI | 2,376 | 0.71 | Internal |
|  | BPMI |  |  | 182,907 | 0.74 | External |
|  | BPMI – mayo clinic |  |  | 2,376 | 0.78 | Internal |
| Liu 2022 (27) | Logistic regression | Neurosurgery | SSI | 288 | 0.71 | Internal |
|  | Decision tree |  |  |  | 0.78 | Internal |
|  | Multilayer perception |  |  |  | 0.76 | Internal |
|  | RF |  |  |  | 0.89 | Internal |
|  | Gradient boosting machine |  |  |  | 0.92 | Internal |
|  | Extreme gradient boosting machine |  |  |  | 0.92 | Internal |
| Liu 2022 (28) | Logistic regression | Abdominal surgery | Organ space SSI | 297 | 0.88 | Internal |
|  | GBDT |  |  |  | 0.83 | Internal |
|  | KNN |  |  |  | 0.71 | Internal |
|  | RF |  |  |  | 0.87 | Internal |
|  | SVM |  |  |  | 0.89 | Internal |
| Mamlook 2023 (29) | Logistic regression | General surgery | SSI | 2,882,526 | 0.82 | Internal |
|  | Naïve bayes |  |  |  | 0.71 | Internal |
|  | RF |  |  |  | 0.84 | Internal |
|  | Decision tree |  |  |  | 0.81 | Internal |
|  | SVM |  |  |  | 0.82 | Internal |
|  | ANN |  |  |  | 0.82 | Internal |
|  | DNN |  |  |  | 0.85 | Internal |
| Nudel  2020 (33) | Logistic regression | Abdominal  surgery | Organ space SSI | 436,807 | 0.63 | Internal |
|  | XGB |  |  |  | 0.70 | Internal |
|  | ANN |  |  |  | 0.75 | Internal |
| Van Esbroeck2014 (37) | CPT  CPT range  RVU  Multivariate model | General surgery | SSI | 361,481 | 0.78  0.65  0.67  0.77 | External |
|  | SVM – short description |  |  |  | 0.79 | External |
|  | SVM – medium description |  |  |  | 0.79 | External |
|  | SVM – large description |  |  |  | 0.79 | External |
|  | SVM – CPT |  |  |  | 0.74 | External |
|  | SVM – multivariate model |  |  |  | 0.80 | External |
| Van Kooten 2022 (38) | Lasso logistic regression  Logistic regression | Abdominal surgery | Organ space SSI | 6,427 | 0.62  0.62 | Internal |
|  | Adaboost |  |  |  | 0.61 | Internal |
|  | Adalearner |  |  |  | 0.62 | Internal |
|  | KNN |  |  |  | 0.57 | Internal |
|  | Neural network |  |  |  | 0.62 | Internal |
|  | RF |  |  |  | 0.59 | Internal |
|  | SVM |  |  |  | 0.59 | Internal |
| Velmahos 2023 (39) | L1-L2-RFE  Logistic regression | Abdominal surgery | Superficial SSI; organ space SSI | 94,530 | 0.64; 0.73  0.64; 0.73 | Internal |
|  | RF |  |  |  | 0.63; 0.72 | Internal |
|  | XGB |  |  |  | 0.64; 0.72 | Internal |
| Weller 2018 (41) | Lasso logistic regression | Abdominal surgery | SSI | 9,598 | 0.49^a^, 0.55^b^, 0.56^c^, 0.56^d^ | Internal |
|  | RF |  |  |  | 0.44^a^, 0.47^b^, 0.50^c^, 0.55^d^ | Internal |
|  | SVM |  |  |  | 0.55^a^, 0.51^b^, 0.47^c^, 0.49^d^ | Internal |
|  | Adaboost |  |  |  | 0.44^a^, 0.47^b^, 0.51^c^, 0.51^d^ | Internal |
|  | Naïve bayes |  |  |  | 0.48^a^, 0.45^b^, 0.45^c^, 0.78^d^ | Internal |
| Ying 2023 (42) | Logistic regression | Orthopedic surgery | SSI | 351 | 0.74 | External |
|  | Extra trees classifier |  |  |  | 0.87 | External |
|  | RF |  |  |  | 0.82 | External |
| Zhang 2023 (43) | Logistic regression | Cardiothoracic surgery | SSI | 1,223 | 0.95 | Internal |
|  | RF |  |  |  | 0.83 | Internal |
|  | SVM |  |  |  | 0.91 | Internal |
|  | XGB |  |  |  | 0.99 | Internal |
|  | GBDT |  |  |  | 0.99 | Internal |
|  | Adaboost |  |  |  | 0.81 | Internal |
|  | NN |  |  |  | 0.99 | Internal |

ACS-NSQIP, American College of Surgeons National Surgical Quality Improvement Program; AdaBoost, Adaptive boosting; ANN, Artificial neural network; ASA, American Society of Anesthesiology; BPMI, Bayesian-probit regression model with multiple imputation; CPT, Current procedural terminology; DNN, Deep neural network; GBDT, Gradient boosted decision trees; GLM, Generalized linear model; KNN, K-nearest neighbours, LASSO, Least absolute shrinkage and selection operator; NB, Naive bayes; OCT, Optimal Classification Trees; POD, Post operative day; POTTER, Predictive OpTimal Trees in Emergency surgery Risk; RF, Random forest; RFE, recursive feature elimination; RVU, Relative Value Unit; SVM, Support vector machine; XGB, Gradient Boosting machine**.** ^a^preoperative, ^b^POD0, ^c^POD1, ^d^POD2
