# Supplementary material for: Systematic evaluation of machine learning models for postoperative surgical site infection prediction
Source: PLoS One. 2024 Dec 12;19(12):e0312968. doi: 10.1371/journal.pone.0312968 (PMC11637340; doi:10.1371/journal.pone.0312968)
Supplement: S4 Table — (DOCX) [file pone.0312968.s006.docx]

# S4 Table. Risk of bias assessment with the use of the PROBAST score

| **Study** | **Model** | **Participants** | **Predictors** | **Outcome** | **Analysis** | **Overall** |
| --- | --- | --- | --- | --- | --- | --- |
| Bertismas, 2018 (20) | POTTER Calculator - OCT 1 without ASA classification | ? | ? | ? | - | - |
|  | POTTER Calculator – OCT 2 with ASA classification | ? | ? | ? | - | - |
| Bonde, 2021 (21) | Neural network model 1 | + | ? | ? | - | - |
|  | Neural network model 2 | + | ? | ? | - | - |
|  | Neural network model 3 | + | ? | - | - | - |
|  | ACS NSQIP Surgical Risk Calculator | + | ? | ? | ? | ? |
|  | POTTER Calculator | + | ? | - | ? | - |
| Chang, 2020 (22) | DLPM | + | ? | ? | - | - |
| El Hechi, 2021 (23) | POTTER | + | ? | ? | - | - |
| Gowd, 2019 (24) | K-Nearest neighbours | + | ? | ? | - | - |
|  | Logistic regression | + | ? | ? | - | - |
|  | Random Forest | + | ? | ? | - | - |
|  | Naive bayes | + | ? | ? | - | - |
|  | Decision tree | + | ? | ? | - | - |
|  | Gradient boosting trees | + | ? | ? | - | - |
|  | ASA Classification | + | ? | ? | - | - |
|  | Frailty index | + | ? | ? | - | - |
| Grass, 2020 (25) | BPMI (Mayo clinic developed) | + | ? | ? | - | - |
|  | BPMI (ACS-NSQIP developed) | + | ? | ? | - | - |
|  | GLM | + | ? | ? | - | - |
| Ke, 2017 (26) | Bilinear model | + | + | - | - | - |
| Liu, W.C., 2022 (27) | Logistic regression | + | - | ? | - | - |
|  | Decision tree | + | - | ? | - | - |
|  | Multilayer perceptron | + | - | ? | - | - |
|  | Random Forrest | + | - | ? | - | - |
|  | Gradient boosting machine | + | - | ? | - | - |
|  | Extreme gradient boosting | + | - | ? | - | - |
| Liu, X, 2022 (28) | Gradient boosting trees | + | + | ? | - | - |
|  | K-nearest neighbours | + | + | ? | - | - |
|  | Random Forest | + | + | ? | - | - |
|  | SVM | + | + | ? | - | - |
|  | WBC | + | + | ? | - | - |
|  | Logistic regression | + | + | ? | - | - |
| Mamlook, 2023 (29) | Logistic regression | + | + | ? | - | - |
|  | Naïve Bayesian | + | + | ? | - | - |
|  | Random Forest | + | + | ? | - | - |
|  | Decision tree | + | + | ? | - | - |
|  | Support Vector Machine | + | + | ? | - | - |
|  | Artificial neural network | + | + | ? | - | - |
|  | Deep Neural Network | + | + | ? | - | - |
| Maurer, 2020 (56) | POTTER Calculator | + | ? | ? | - | - |
| Mazaki, 2021 (31) | Artificial neural network | - | ? | ? | - | - |
| Merath, 2020 (32) | the Machine Learning-Based Decision-Tree Learning Algorithm | + | ? | ? | - | - |
| Nudel, 2021 (33) | Artificial neural network | + | ? | - | - | - |
|  | Gradient boosting machine | + | ? | - | - | - |
|  | Logistic regression | + | ? | - | - | - |
| Ohno, 2020 (34) | SVM | + | + | - | - | - |
| Sanger, 2016 (35) | Prognostic Model of Surgical Site Infection Using Daily Clinical Wound Assessment - Baseline features | + | + | ? | - | - |
|  | Prognostic Model of Surgical Site Infection Using Daily Clinical Wound Assessment - Serial features | + | + | ? | - | - |
|  | Prognostic Model of Surgical Site Infection Using Daily Clinical Wound Assessment - Simplified serial features | + | + | ? | - | - |
|  | Prognostic Model of Surgical Site Infection Using Daily Clinical Wound Assessment - Combined baseline and serial features | + | + | ? | - | - |
| Taylor, 2019 (36) | Generalize additive models | + | ? | ? | - | - |
|  | LASSO | + | ? | ? | - | - |
|  | Random forest classifier | + | ? | ? | - | - |
|  | Feed-forward neural network with logistic activation function and no weight decay | + | ? | ? | - | - |
| Van Esbroeck, 2014 (37) | SVM - Short description lengths | + | ? | ? | - | - |
|  | Current procedural terminology range | + | ? | ? | - | - |
|  | Current procedural terminology | + | ? | ? | - | - |
|  | Multivariate model | + | ? | ? | - | - |
|  | Multivariate model + SVM | + | ? | ? | - | - |
|  | Relative value unit | + | ? | ? | - | - |
|  | SVM - current procedural terminology (CPT) | + | ? | ? | - | - |
|  | SVM - Large description lengths | + | ? | ? | - | - |
|  | SVM - Medium description lengths | + | ? | ? | - | - |
| van Kooten, 2022 (38) | Adaboost | + | + | ? | - | - |
|  | Adalearner | + | + | ? | - | - |
|  | K-Nearest neighbours | + | + | ? | - | - |
|  | Neural network | + | + | ? | - | - |
|  | Random Forrest | + | + | ? | - | - |
|  | SVM | + | + | ? | - | - |
|  | Lasso logistic regression | + | + | ? | - | - |
|  | Logistic regression | + | + | ? | - | - |
| Velmahos, 2023 (39) | Random forest | + | + | ? | - | - |
|  | Extreme gradient boosting | + | + | ? | - | - |
|  | L1-L2-RFE | + | + | ? | - | - |
|  | Logistic regression | + | + | ? | - | - |
| Walczak, 2019 (40) | Prophylactic antibiotic bundle compliance - all variables | + | ? | ? | - | - |
|  | Prophylactic antibiotic bundle compliance - excluding NSQIP compliance variable | + | ? | ? | - | - |
|  | Prophylactic antibiotic bundle compliance - excluding NSQIP compliance and sex variable | + | ? | ? | - | - |
| Weller, 2018 (41) | Random Forrest preoperative | + | - | - | - | - |
|  | Random Forrest POD0 | + | - | - | - | - |
|  | Random Forrest POD1 | + | - | - | - | - |
|  | Random Forrest POD2 | + | - | - | - | - |
|  | SVM preoperative | + | - | - | - | - |
|  | SVM POD0 | + | - | - | - | - |
|  | SVM POD1 | + | - | - | - | - |
|  | SVM POD2 | + | - | - | - | - |
|  | AdaBoost preoperative | + | - | - | - | - |
|  | AdaBoost POD0 | + | - | - | - | - |
|  | AdaBoost POD1 | + | - | - | - | - |
|  | AdaBoost POD2 | + | - | - | - | - |
|  | Naive bayes preoperative | + | - | - | - | - |
|  | Naive bayes POD0 | + | - | - | - | - |
|  | Naive bayes POD1 | + | - | - | - | - |
|  | Naive bayes POD2 | + | - | - | - | - |
|  | LASSO logistic regression preoperative | + | - | - | - | - |
|  | LASSO logistic regression POD0 | + | - | - | - | - |
|  | LASSO logistic regression POD1 | + | - | - | - | - |
|  | LASSO logistic regression POD2 | + | - | - | - | - |
| Ying, 2023 (42) | Extra Trees Classifier | + | - | - | - | - |
|  | Logistic regression | + | - | - | - | - |
|  | Random forest | + | - | - | - | - |
| Zhang, 2023 (43) | Random Forest | + | - | - | - | - |
|  | SVM | + | - | - | - | - |
|  | Extreme gradient boosting | + | - | - | - | - |
|  | Gradient boosting decision tree | + | - | - | - | - |
|  | Adaboost | + | - | - | - | - |
|  | Logistic regression | + | - | - | - | - |
|  | Neural network | + | - | - | - | - |

ACS-NSQIP, American College of Surgeons National Surgical Quality Improvement Program; AdaBoost, Adaptive boosting; ASA, American Society of Anesthesiology; LASSO, Least absolute shrinkage and selection operator; OCT, Optimal Classification Trees; POTTER, Predictive OpTimal Trees in Emergency Surgery Risk; SVM, Support vector machine; WBC, White blood cell count.

+ indicates low risk of bias; - indicates high risk of bias; ? indicates unclear risk of bias
